# Synthesis and mechanistic investigations of pH-responsive cationic poly(aminoester)s†

**DOI:** 10.1039/c9sc05267d

**Published:** 2020-02-20

**Authors:** Timothy R. Blake, Wilson C. Ho, Christopher R. Turlington, Xiaoyu Zang, Melanie A. Huttner, Paul A. Wender, Robert M. Waymouth

**Affiliations:** Department of Chemistry, Stanford University Stanford CA 94305 USA waymouth@stanford.edu; Department of Chemical and Systems Biology, Stanford University Stanford CA 94305 USA

## Abstract

The synthesis and degradation mechanisms of a class of pH-sensitive, rapidly degrading cationic poly(α-aminoester)s are described. These reactive, cationic polymers are stable at low pH in water, but undergo a fast and selective degradation at higher pH to liberate neutral diketopiperazines. Related materials incorporating oligo(α-amino ester)s have been shown to be effective gene delivery agents, as the charge-altering degradative behavior facilitates the delivery and release of mRNA and other nucleic acids *in vitro* and *in vivo*. Herein, we report detailed studies of the structural and environmental factors that lead to these rapid and selective degradation processes in aqueous buffers. At neutral pH, poly(α-aminoester)s derived from *N*-hydroxyethylglycine degrade selectively by a mechanism involving sequential 1,5- and 1,6-O→N acyl shifts to generate bis(*N*-hydroxyethyl) diketopiperazine. A family of structurally related cationic poly(aminoester)s was generated to study the structural influences on the degradation mechanism, product distribution, and pH dependence of the rate of degradation. The kinetics and mechanism of the pH-induced degradations were investigated by ^1^H NMR, model reactions, and kinetic simulations. These results indicate that polyesters bearing α-ammonium groups and appropriately positioned *N*-hydroxyethyl substituents are readily cleaved (by intramolecular attack) or hydrolyzed, representing dynamic “dual function” materials that are initially polycationic and transform with changing environment to neutral products.

## Abstract 2

The synthesis and degradation mechanisms of a class of pH-sensitive, rapidly degrading cationic poly(α-aminoester)s are described.

## Graphical abstract

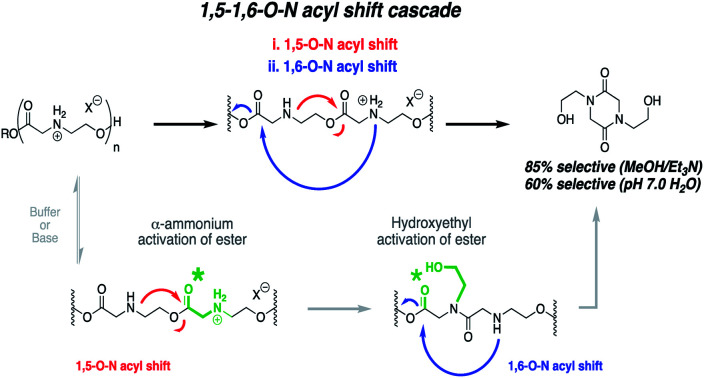

## Introduction

Self-degrading polymers are important dynamic synthetic materials that undergo a rapid degradation in response to environmental stimuli.^1–3^ These stimuli-responsive degradations are now being leveraged in biomedical materials as a means to initiate complexation and then release therapeutic cargos under specific biological conditions/environments.^1–9^ These features provide an attractive platform for the delivery of compounds, such as drugs, proteins or polynucleotides, which must be protected until they reach their therapeutic destination. Several classes of stimuli-responsive degrading materials have been reported in the literature: end-capped systems that upon cleavage of a terminal group undergo an end-to-end (or self-immolative) degradation, and systems in which the polymer backbone is cleaved by appropriately positioned nucleophiles. Self-immolative polymers that undergo quinone (or azaquinone) methide elimination upon removal of an endcap have been investigated in detail by Shabat,^10^ Gillies,^7,11^ Phillips,^12–14^ and others.^1,3^ Various types of stimuli have been explored to trigger the removal of the terminal end-group, including pH changes, enzymatic activities, or oxidative cleavage with H_2_O_2_.

The potential toxicity of the (aza)quinone methide byproducts^15^ has stimulated research into alternative chemistries for stimuli-sensitive degradation processes,^16^ including disulfide-based self-immolative systems that degrade upon reduction,^17,18^ acid-labile polymers,^19–22^ or amine-functionalized polyesters and polycarbonates that degrade either through intramolecular nucleophilic attack or by hydrolysis.^11,23–31^

Cationic poly(aminoester)s are an attractive class of materials due to the tunable lability of the ester linkage^32^ and the functional cationic ammonium repeat units that can bind appropriate polyanionic cargoes and then become neutral, enabling cargo release. Several classes of cationic poly(aminoester)s have been shown to undergo pH-sensitive degradation resulting from deprotonation of embedded ammonium groups, which leads to nucleophilic attack on the ester backbone.^23–25,30,33^ Cationic poly(β-aminoester)s have demonstrated good biocompatibility and are used in a wide range of biomedical applications,^34,35^ highlighted by recent advances in gene delivery vehicles for a variety of therapeutic indications.^36–43^

**Fig. 1:**
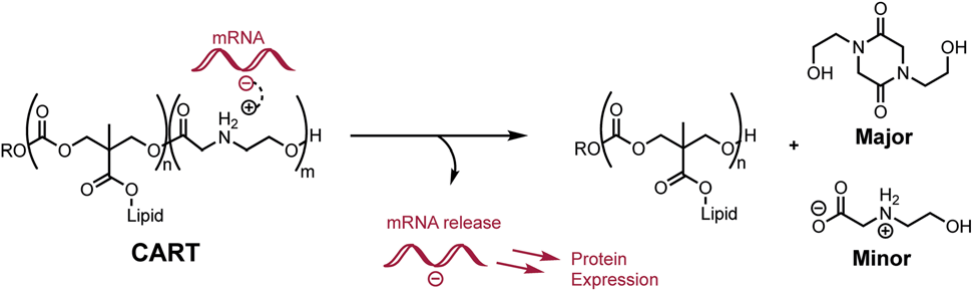
Proposed mRNA binding and release by Charge-Altering Releasable Transporters (CARTs). Selective degradation of the polycationic poly(α-aminoester) block generates neutral diketopiperazines and hydrolyzed *N*-hydroxyethyl glycines.

We recently reported a novel class of materials based on cationic poly(α-aminoester) backbones that undergo a pH-sensitive rapid and selective degradation to neutral amide and amino acid byproducts in water.^44^ We have leveraged this behavior in the design of novel cationic amphiphilic oligomers, charge-altering releasable transporters (CARTs),^33,45–47^ that bind, complex, and deliver oligonucleotides of wide ranging size (mRNA, pDNA) into cells, *in vitro* and *in vivo*.^33,45–49^ At low pH, these oligomeric amphiphiles are cationic and associate in water with polyanionic oligonucleotides to form polyelectrolyte complexes. At elevated pH these polyelectrolyte complexes release the oligonucleotides, which was attributed to the charge-altering degradative behavior of the cationic oligo(α-amino ester)s (Fig. 1). These charge-altering releasable transporters (CARTs)^33,45–47^ were shown to be effective for mRNA and pDNA delivery to a variety of cell types in cell culture and live animals, enabling multiple therapeutic vaccination strategies for eradication of established tumors in mice.^48,49^

As the pH-induced charge-canceling degradation of the cationic poly(α-aminoester) backbone was implicated in the intracellular release of mRNA,^33^ herein we describe a detailed mechanistic study directed at elucidation of the structural and environmental factors that lead to the selective degradation processes for the cationic poly(ammonium ester) homopolymers in aqueous buffers. While not directly comparable to the polyelectrolyte environments in which the amphiphilic CARTs degrade,^33,45–47^ these studies provide fundamental insights on the structural factors that contribute to the degradation behavior of poly(amino ester)s in aqueous buffers. We report the synthesis of a family of structurally related cationic polyaminoester homopolymers and investigate how their structural variations influence the mechanism, product distribution, and pH dependence of their degradation.

## Results

**Fig. 2:**
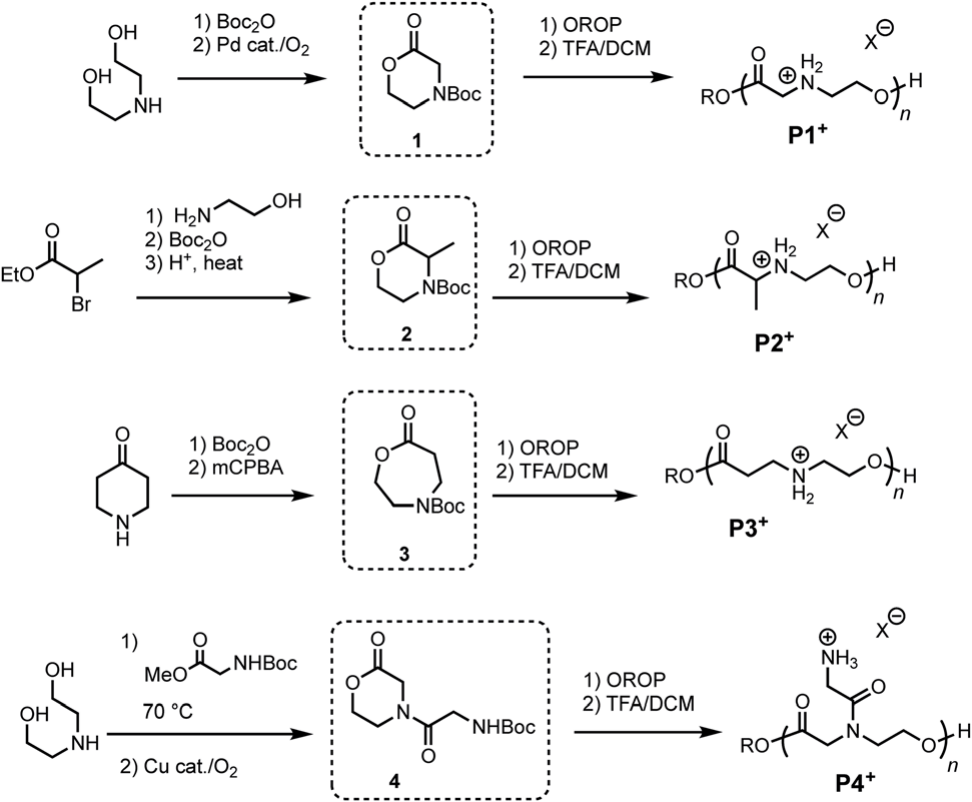
Synthesis of azalactone monomers and polymers.

To investigate the influence of poly(amino ester) structure on the rate and mechanism of their degradation, a series of monomers **1–4** were synthesized in 1–3 steps from commercially available reagents (Fig. 2 and Table 1). The morpholin-2-one **1** was generated by a Pd-catalyzed oxidative lactonization, as previously reported.^44^ The α-methyl substituted morpholinone **2** was prepared by a 3-step procedure in one flask;^50^ this approach provides a general strategy for introducing a variety of substituents at the α- and γ-positions. Azacaprolactone **3** was synthesized *via* the ring-expansion Baeyer–Villager oxidation of the Boc-protected piperidone (1–2 steps), while *N*-glycyl morpholinone **4** was made *via* Cu-catalyzed aerobic oxidative lactonization (2 steps).^51^ As both of these latter reactions are compatible with amides and carbamates, these pathways provide access to a wide variety of *N*-substituents including the proteinogenic amino acids.

**Table 1:** Synthesis and OROP of new monomers^a^

Monomer synthesis		Polymerizations						
Monomer	Yield^b^ (%)	Solvent	[M] (M)	[I] (M)	[C] (M)	DP	*M*_n_ (kDa)	*Đ*
**1**	60	PhMe	1.0	0.015	0.05	65	14.5	1.12
**2**	29	PhMe	4.7	0.05	0.26	49	6.9	1.36
**3**	73	PhMe	2.3	0.024	0.8	75	18.9	1.10
**4**	76	DCM	1.0	0.05	0.025	74	2.0	1.22

a M = monomer, I = initiator (1-pyrenebutanol), and C = catalyst (1 : 1 TU/DBU). DP was determined by ^1^H NMR end-group analysis. *M*_n_ and *Đ* were determined by PS-calibrated gel permeation chromatography.

b Based on the first starting material shown in each scheme of Fig. 2.

Monomers **1–4** undergo controlled organocatalytic ring-opening polymerization (OROP) with the thiourea/DBU catalyst system at room temperature (Table 1). With initial monomer concentrations of [M]_0_ = 1.0 M in toluene, **1** and **3** reached equilibrium conversions of 87% and >95% (see ESI†), respectively, as determined by ^1^H NMR. α-Me morpholinone **2** required substantially higher initial monomer concentration for polymerization (at [M]_0_ = 4.0 M conversion reached 52%), which we attribute to the less favorable thermodynamics of ring-opening.^44,52^ The *N*-glycyl amide functionalized lactone **4** exhibited poor solubility in toluene and was thus polymerized in dichloromethane (DCM). Although prior studies had shown^44^ that the ring-opening thermodynamics were less favorable in DCM than in toluene, the polymerization of **4** in this solvent proceeded to 63% conversion when [M]_0_ = 1.0 M. The polymers generated from **4** represent a new subclass of functionalized polyesters derived from amino acids (Fig. 2, **P4+**). Deprotection of each polymer **1–4** with trifluoroacetic acid (TFA) in DCM afforded cationic poly(ammonium ester)s that are water-soluble and stable in unbuffered D_2_O for >24 hours.

### Degradation of cationic poly(aminoester) **P1+**

**Fig. 3:**
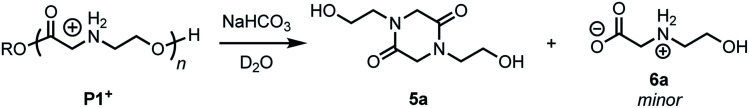
Degradation products and base-triggered degradation of **P1+** with saturated NaHCO_3_ in H_2_O.

Treatment of the cationic poly(α-aminoester) **P1+** (*M*_n_ = 14.5 kDa, *Đ* = 1.12) with saturated aqueous NaHCO_3_ (pH ∼ 9) for 30 minutes resulted in the rapid degradation of the cationic polyester; the major product at the end of this degradation was the diketopiperazine **5a**, isolated in 72% yield. *N*-Hydroxyethylglycine (**6a**) formed by hydrolysis was also identified as one of the minor products (Fig. 3).

**Fig. 4:**
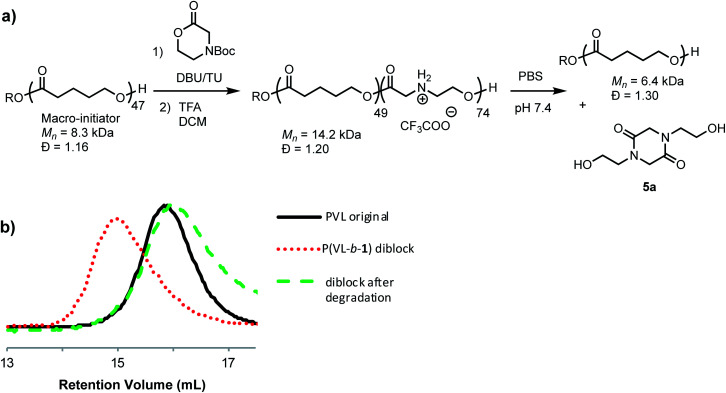
Degradation of P(VL-*b*-**1+**) diblock copolymer. (a) Synthesis and reaction scheme. (b) GPC traces of polymers. Poly(valerolactone) macroinitiator (black line); protected P(VL-*b*-**1**) diblock (dotted red line); products after degradation of deprotected P(VL-*b*-**1+**) (dashed green line).

To assess the relative rate of degradation of the cationic poly(α-aminoester) **P1+** relative to an analogous unfunctionalized aliphatic polyester, we investigated the degradation of a diblock copolymer of poly(valerolactone) (pVL) and **P1+** in aqueous solution (Fig. 4). The diblock copolymer P(VL-*b*-**1**) was prepared from a pVL macroinitiator (*M*_n_ = 8.3 kDa, *Đ* = 1.16) and monomer **1** (Fig. 4a). After deprotection, the water-soluble copolymer P(VL-*b*-**1+**) was dissolved in phosphate-buffered saline (PBS, pH 7.4). After 1 hour, analysis of the resulting sample by gel-permeation chromatography (GPC) and ^1^H NMR revealed the predominant products to be **5a** and poly(valerolactone) with an *M*_n_ of 6.4 kDa (*Đ* = 1.30). These results showed that the aqueous degradation of **P1+** was much faster than that of the unfunctionalized polyester pVL. While the cationic segment **P1+** had degraded completely, the valerolactone segment had only been partially hydrolyzed, as evidenced by the slight decrease in the molecular weight and slight broadening of the molecular weight distribution (dashed green *vs.* solid black trace, Fig. 4b). Previous studies showed that **P1+** also degrades much more quickly than a polycarbonate block in an analogous copolymer.^33^

### Degradation kinetics of cationic poly(aminoester) **P1+**

**Fig. 5:**
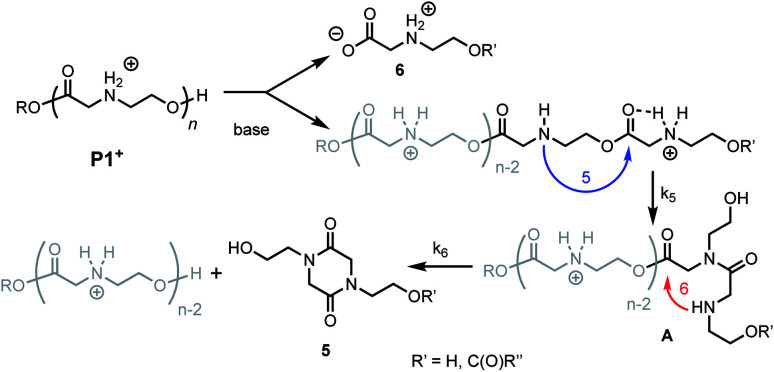
Proposed mechanism of degradation of **P1+**. Blue arrow, *k*_5_ = 5-membered cyclization. Red arrow, *k*_6_ = 6-membered cyclization.

Prior studies^33^ of the highly selective degradation of **P1+** implicated a mechanism that involved an alternating 1,5- and 1,6-cyclization cascade that liberated neutral diketopiperazines (*e.g.***5**, Fig. 5). In this proposed mechanism, as the pH increases, some fraction of the ammonium groups are deprotonated to nucleophilic amines which then undergo an intramolecular rearrangement (1,5-O→N acyl shift)^53^ to generate hydroxyethyl amides (**A**, Fig. 5). This rearrangement contracts the backbone, allowing a subsequent 6-membered cyclization (1,6-O→N acyl shift) to liberate the neutral diketopiperazine. This 1,5-1,6 rearrangement sequence repeats until much of the polymer is consumed, producing **5** as the predominant final product.

The kinetics of degradation of the cationic poly(aminoester) **P1+** (*M*_n_ = 14.5 kDa, *Đ* = 1.12) were investigated in aqueous buffers as a function of pH. To facilitate kinetic modeling, we employed an experimental approach^11,27,29^ that monitored the decay of the *N*-hydroxyethylglycine ester repeat units in the chain, as well as the appearance of identifiable products (see below) as a function of time by ^1^H NMR.

**Fig. 6:**
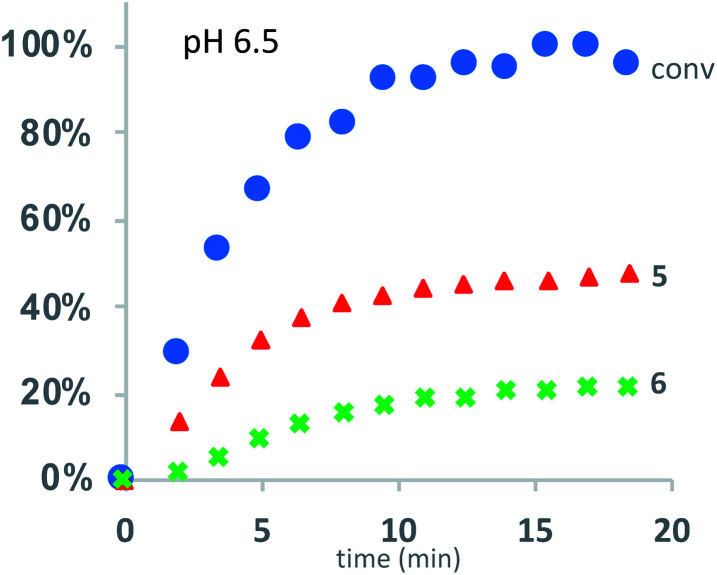
Kinetics for the degradation of **P1+** in D_2_O at pH 6.5: conversion of starting material (blue dots); yield of diketopiperazines **5** (red triangles); yield of acids **6** (green crosses).

Under buffered aqueous conditions, two major product types could be quantified by ^1^H NMR: diketopiperazines **5** and α-amino acids **6** (R′ = H, C(O)R″), the latter arising from hydrolysis (Fig. 5). These products were identified by comparison to the ^1^H NMR spectra of **5a** and **6a** (R′ = H) prepared independently. Minor amounts of linear *N*-hydroxyethylamides were visible in the spectra but not readily quantified. The reaction kinetics at pH 6.5 were monitored for approx. 3 hours, at which point the polymer had degraded to diketopiperazines **5** (from 54% of the starting hydroxyethyl glycine units) and **6** (from 28% of the starting units). Under these conditions, the disappearance of repeat units in the chain occurred with an empirical half-life of *t*_1/2_ = 3.8 min (*t*_1/2_ = time at which 50% conversion is reached). A representative time course for the degradation of **P1+** at pH 6.5 is given in Fig. 6 and data for all experiments are summarized in Table 2.

**Table 2:** Polymer degradation kinetics^a^

Entry	Polymer^b^	pH^c^	*t*_1/2_ (min)	Time^d^ (h)	Conv.^e^ (%)	Pdt. 1^f^ (%)		Pdt. 2^f^ (%)	
1	**P1+**	5.1	29	3.0	87	**5**	49	**6**	29
2	**P(1+Cl−)**	5.1	33	3.0	84	**5**	48	**6**	34
3	**P1+**	6.5	3.8	1.5	100	**5**	54	**6**	28
4	**P1+**	7.0	<3	1.0	96	**5**	59	**6**	15
5	**P1+**	Et_3_N^g^	6.4	3.0	86	**5**	85	**6**	0
6	**P2+**	5.1	77	3.0	87	—	—	**8**	85
7	**P2+**	6.5	7.6	0.4	94	—	—	**8**	97
8	**P3+**	7.0	201	10.0	69	**9**	35	**10**	33
9	**P3+**	7.5	42	3.0	69	**9**	52	**10**	18
10	**P3+**	∼9^h^	13	16.8	100	**9**	85	**10**	<5
11	**P4+**	5.1	443	10.4	56	**11**	45	**12**	9
12	**P4+**	6.5	15	3.0	100	**11**	87	**12**	12
13	**P4+**	7.0	<3	1.0	100	**11**	95	**12**	5

a Reactions done in duplicate at 25 °C, [polymer]_0_ = 40–60 mM (calculated with respect to repeat units) in a 1 : 1 mixture of buffer and D_2_O and quantified by ^1^H NMR with an internal standard. Dashed entries unknown or not accurately quantified.

b Default counteranion trifluoroacetate; typical polymer lengths were **P165+**, **P248+**, **P375+**, and **P448+**.

c All pH degradation experiments were conducted in deuterated NMR buffers. The pH indicated were measured upon dilution of these buffers with distilled H_2_O. The pH 5.1 buffer was made with acetic acid-*d*_4_/NaOH, and the pH 6.5, 7.0, and 7.5 buffers with KH_2_PO_4_/K_2_HPO_4_ in D_2_O (see ESI for details).

d Time at which the next three data columns are measured.

e Percentage of starting esters that have disappeared.

f Percentage of starting esters incorporated into this product.

g Degradation carried out with 2.5 eq. Et_3_N in CD_3_OD.

h Saturated NaHCO_3_ in D_2_O, used without dilution.

At the more acidic pH 5.1, the cationic polyester **P1+** degrades more slowly (*t*_1/2_ = 29 min) over the course of 3 hours. By this time, 87% of the *N*-hydroxyethylglycine esters have reacted to afford **5** (49% yield), **6** (29% yield), and other products that are not readily identified. At higher pH, the rate of degradation and the selectivity for the diketopiperazines increase (Table 2).

These data reveal the strong influence of pH on the rate of degradation of the poly(α-aminoester) **P1+** in aqueous solution. At pH 7.0, 50% of the hydroxyethyl esters of **P1+** have rearranged or degraded in less than 3 minutes, whereas at pH 5.1 this takes 29 min. At all pH values, diketopiperazines are the major product. Control experiments indicate that the rates and selectivities of the degradation are not strongly influenced by the nature of the counteranion (CF_3_COO^−^*vs.* Cl^−^, Table 2 entry 1 *vs.* 2).

**Fig. 7:**
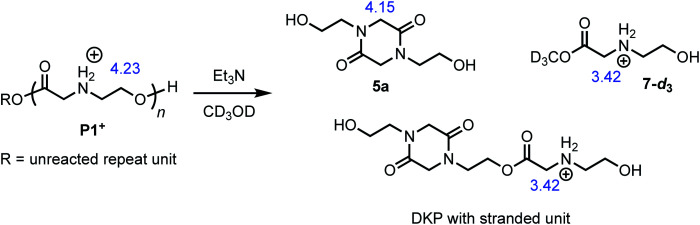
Degradation of **P1+** in CD_3_OD/Et_3_N to liberate diketopiperazine **5a** and *N*-hydroxyethyl glycinate esters (R = CD_3_ (**7-d3**) or diketopiperazyl). The blue numbers indicate the ^1^H NMR shifts of representative proton resonances.

The degradation of **P1+** can also be observed in methanol-*d*_3_ in the presence of Et_3_N (Fig. 7); under these conditions, two major products could be identified: those derived from diketopiperazines (R′ = H (**5a**) or C(O)R″) and those derived from *N*-hydroxyethyl glycinate esters (R = CD_3_ (**7-d3**) or diketopiperazyl).

The degradation of the poly(α-aminoester) **P1+** (*M*_n_ = 10.6 kDa, *Đ* = 1.42) in CD_3_OD in the presence of 2.5 eq. Et_3_N (125 mM) was studied by ^1^H NMR by monitoring the disappearance of *N*-hydroxyethylglycine ester units as a function of time. The reaction kinetics were monitored for approximately 3 h, at which point 86% of the hydroxyethyl glycine repeating units had been converted, predominantly to diketopiperazines (**5**, 85% yield, Table 2, entry 5). Under these conditions, the disappearance of the repeating units in the chain occurred with an empirical half-life of *t*_1/2_ = 6.4 min (empirical *t*_1/2_, the time at which half of the monomer units had been converted, was used to simplify comparing mixed order degradation kinetics).

As only 85% of the repeat units transformed to diketopiperazines and conversion would halt after this point, we hypothesized that the structural requirements of the mechanism (namely, that two unreacted ester units must be adjacent to each other) were responsible for this limit on conversion. We modeled the degradation kinetics using stochastic simulations^54,55^ averaged over 1000 trials (see ESI†) under the assumption that only two processes had contributed to the degradation in CD_3_OD: a 1,5-O→N acyl shift characterized by the rate constant *k*_5_ and a subsequent 1,6-O→N acyl shift characterized by the rate constant *k*_6_ (Fig. 5).

Three kinetic models were evaluated with two of the models (S1 and S3) assuming that the initial 1,5-cyclization occurred with equal probability at any hydroxyethyl unit in the polymer chain (random simulations S1 and S3, DP = 65) while the other model constrained the rearrangements to occur only at the hydroxyl chain terminus (end-to-end simulation S2, DP = 64). For simulations S1 and S2, we made the simplifying assumption that the rate constant *k*_6_ was much larger than *k*_5_ (*k*_6_/*k*_5_ = 10^6^); for S3 the rate constants *k*_5_ and *k*_6_ were set to be equal (*k*_6_/*k*_5_ = 1). The fit to experimental data was carried out by adjusting the magnitude of *k*_5_ so that the simulated and experimental conversions would intersect at 50%.

**Fig. 8:**
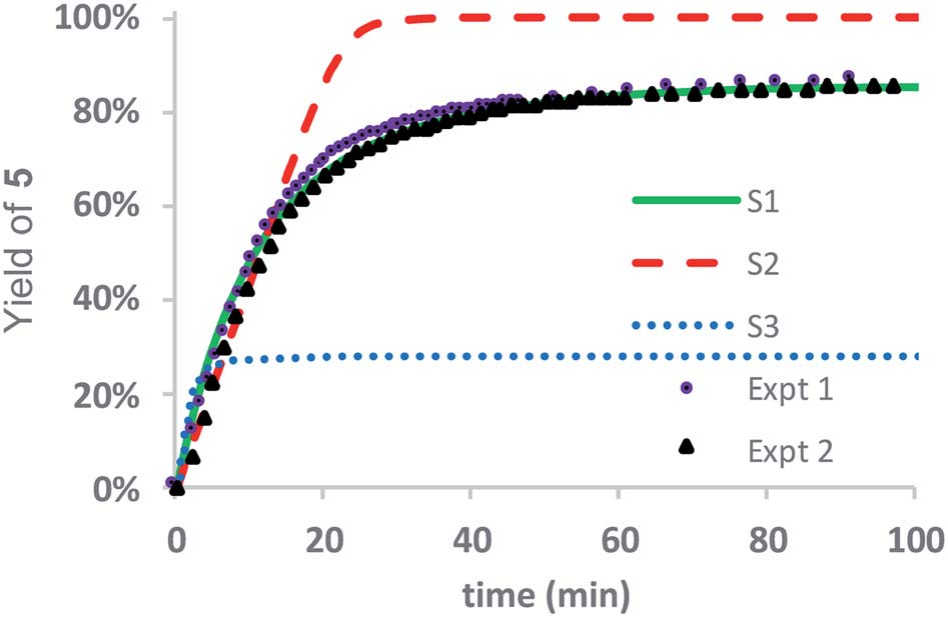
Stochastic kinetic simulations for the yield of diketopiperazine **5**. S1 (green line, *k*_6_ ≫ *k*_5_ = 0.0412 min^−1^); S2 (dashed red line, *k*_6_ ≫ *k*_5_ = 1.44 min^−1^); S3 (dotted blue line, *k*_5_ = *k*_6_ = 0.483 min^−1^); Expt 1 (purple circles); Expt 2 (black triangles).

As shown in Fig. 8, the random model S1 (*k*_6_/*k*_5_ = 10^6^) provides an excellent fit to both sets of experimental data, matching not only the yield but also the production of diketopiperazines as a function of time. In contrast, the end-to-end model S2 (*k*_6_/*k*_5_ = 10^6^) predicts that all of the *N*-hydroxyethyl glycine units of the chain should be converted to diketopiperazines and the random model S3 with *k*_5_ = *k*_6_ predicts that the yield of diketopiperazines should not exceed 28%.

These simulations suggest that the degradation of **P1+** is initiated by random 1,5-O→N acyl shifts that occur at any point along the polymer chains and that the rate of the 1,6-O→N acyl shift is faster than that of the preceding 1,5-O→N acyl shift. As the generation of **5** consumes two adjacent hydroxyethyl glycine repeat units and creates a break in the chain, these cyclization events will randomly leave some of the *N*-hydroxyethyl glycine units in the chain “stranded” and incapable of generating a diketopiperazine (Fig. 7). This is analogous to the situation calculated by Flory for intramolecular reactions of adjacent reactive units in polymer chains.^56,57^ The faster rate of the 1,6-O→N acyl shift (relative to the 1,5-O→N acyl shift) suggested by the simulations is needed to reproduce the high selectivity for cyclization to the diketopiperazine **5** in competition with consecutive 1,5-O→N acyl shifts to yield the linear amides, which are observed experimentally only in trace amounts.

### Influence of structure on degradation of poly(aminoester)s

**Fig. 9:**
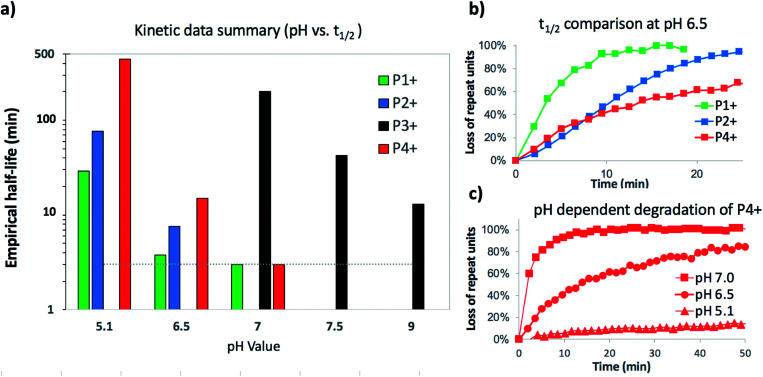
Summarized kinetic data (a) histogram of empirical half-lifes of polymers **P1+–P4+** at different pH (*dashed line represents detection limit for ^1^H NMR experiment). (b) Comparison of **P1+**, **P2+**, and **P4+** at a single pH 6.5. (c) Data comparing degradation of **P4+** at pH 5.1, 6.5 and 7.0.

To assess the structural features that contribute to the rapid and selective base-induced degradation of poly(aminoester)s, we carried out a series of comparative experiments on the degradation behavior of the poly(α-aminoester) **P1+** with the α-methyl substituted poly(α-aminoester) **P2+**, the poly(β-aminoester) **P3+**, and the poly(α-amidoester) **P4+** bearing a pendant glycine (Fig. 1). All four cationic polymers **P1+–P4+** degrade readily in aqueous buffer, but the rates and products differed significantly as a function of the polymer structure (Table 2 and Fig. 9).

### α-Me substituted poly(α-aminoester) **P2+**

The degradation of **P2+** is considerably slower than that of **P1+** at pH 5.1 (*t*_1/2_ = 77 min *vs.* 29 min), but the difference is smaller at pH 6.5 (7.6 min *vs.* 3.8 min). In stark contrast to **P1+**, the final product observed for **P2+** at this pH was *N*-hydroxyethylalanine (**8**) formed by hydrolysis in 97% yield; no diketopiperazine was observed (eqn (1)). This observation reveals that the presence of an α-methyl group (**P2+***vs.***P1+**) significantly influences not only the rate of degradation but also the mechanism; for **P2+** hydrolysis of the esters in the backbone is the predominant mechanism of degradation.1
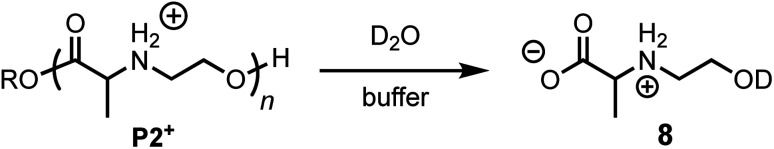


**Fig. 10:**
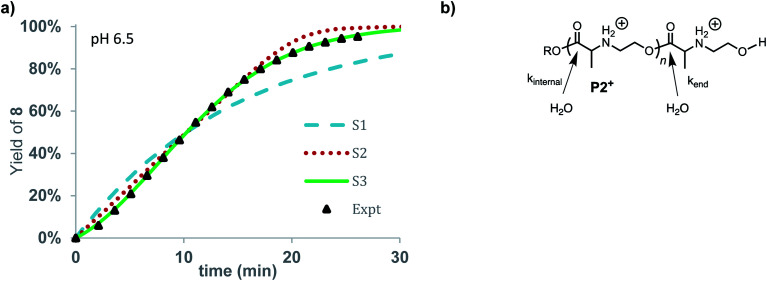
Degradation simulation of **P2+**. (a) Yield of **8** vs. time. Expt. data (black triangles), stochastic simulations (S1 = dashed blue, S2 = dotted red, S3 = solid green line). (b) Model for hydrolysis of **P2+**, at chain-end vs. internal esters.

The rate of formation of hydroxyethyl alanine units (**8**) from **P2+** differs significantly from the formation of hydroxyethyl glycine units (**6**) from **P1+** (Fig. 10a*vs.*Fig. 6 and 8). At short times, the rate of formation of **8** increases and then occurs at a constant rate until approx. 75% conversion (Fig. 10a).

The rate of formation of **8** could be simulated using stochastic simulations where hydrolysis of the esters in the chain is assumed to be the only degradation process. Three simulations were carried out: the first (S1) with a single rate constant for hydrolysis at any ester in the chain (*k*_h_), the second (S2) where hydrolysis occurs exclusively at the chain-end (*k*_end_), and the third (S3) where hydrolysis processes of different rate constants occur both at the chain-ends (*k*_end_) and at internal esters (*k*_int_). The kinetic data and simulated fits are shown in Fig. 10. As shown in Fig. 10, random simulation S1 with a single rate constant for hydrolysis yields a poor fit to the experimental data as this mechanism would predict a first-order appearance of hydroxyethyl alanine **8**. The end-to-end simulation S2 yields a better fit to the data but does not capture the slight acceleration at early times or the decay in rate after 80% conversion. The best fit to the experimental data was obtained with simulation S3 when the hydrolysis rate of the esters at the chain-end were faster (*k*_end_ = 0.602 min^−1^) than those of internal esters in the polymer chain (*k*_int_ = 0.0144 min^−1^).

The results of these simulations imply that esters at the chain-end are more reactive to hydrolysis than internal esters of the polymer chain, as the simulations based on either exclusive (S2) or accelerated hydrolysis at the chain-end (S3) yield much better fits than the simulation (S1) based on uniform hydrolysis at any ester in the chain. While S3 yields a slightly better fit to the data than S2, the difference is not large enough to justify a specific conclusion about how much larger *k*_end_ is relative to *k*_int_.

### Poly(β-aminoester) **P3+**

The aqueous degradation of **P3+** is significantly slower than that of the poly(α-aminoester) **P1+** (*t*_1/2_ (pH 7.0) = 201 min *vs.* <3 min) yielding a mixture of linear oligo(*N*-hydroxyethylamide)s (**9**) and products from hydrolysis (**10**, eqn (2), Table 2). At higher pH, the selectivity for the formation of linear amides increases; in saturated sodium bicarbonate (NaHCO_3_), the linear amides account for 85% of the products. Unlike the other 3 polymers, the degradation products of **P3+** are primarily oligoamides instead of small molecules.2
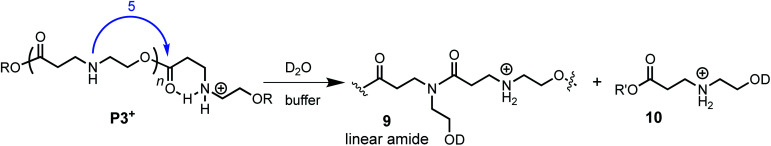


The formation of linear amides is consistent with a proposed mechanism where 1,5-O→N acyl shifts occur along the backbone. For **P3+**, the liberation of a cyclic dipeptide is not observed, as a subsequent O→N acyl shift in the opposite direction would require the formation of an 8-membered ring. The much slower rate of **P3+** degradation compared to **P1+** can be attributed to the diminished activating effect of the β-ammonium on the ester in **P3+** relative to the α-ammonium in **P1+**.^58^

**Fig. 11:**
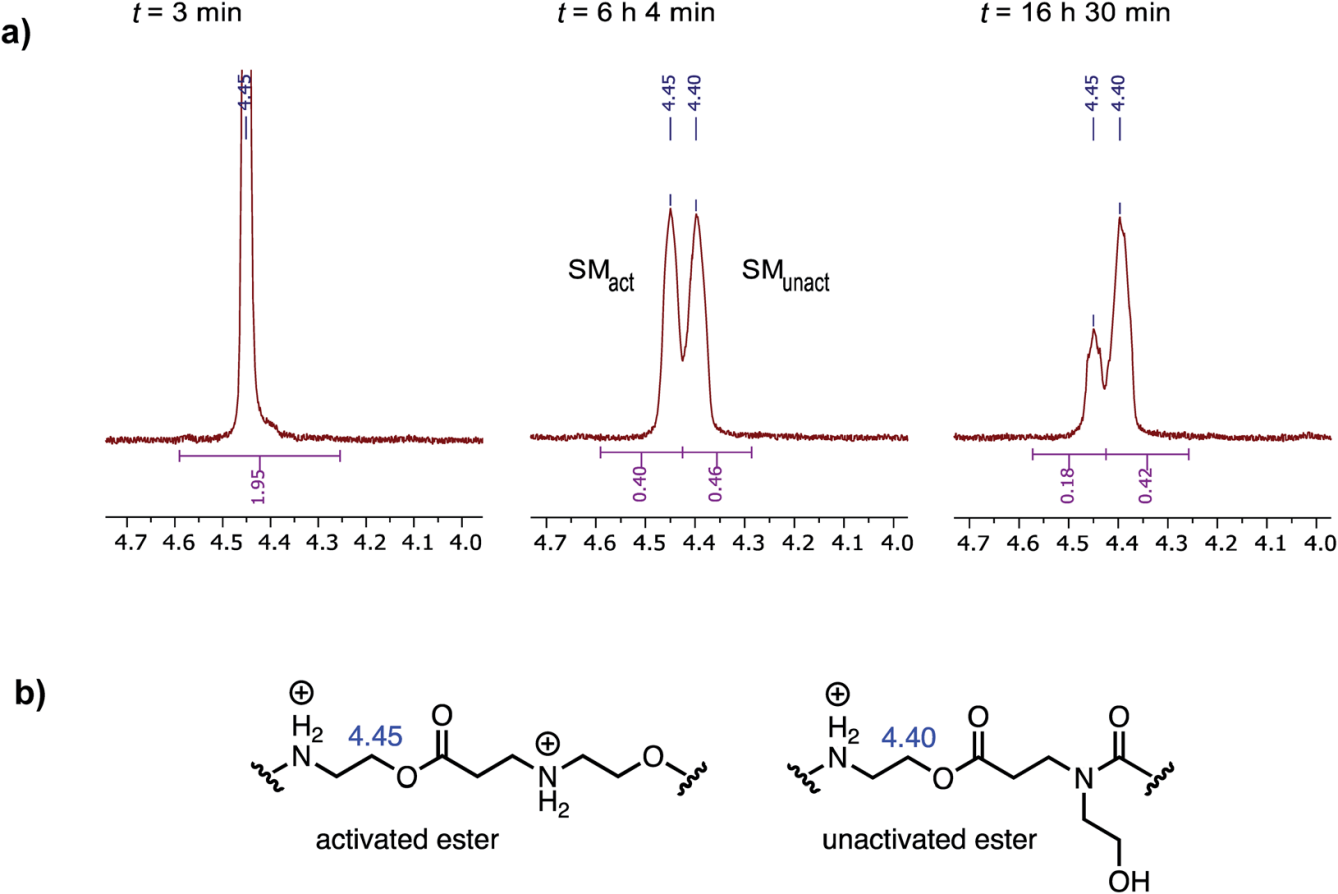
(a) ^1^H NMR resonances corresponding to *N*-hydroxyethyl repeat units in polymer **P3+** (D_2_O, pH 7.0) from three time points (activated ester = SM_act_, unactivated ester = SM_unact_). (b) Structural assignments for ^1^H NMR resonances at *δ* = 4.45 and 4.40 ppm.

When the degradation of **P3+** was monitored by ^1^H NMR in D_2_O at pH 7.0, resonances corresponding to the hydroxyethyl β-ammonium esters at *δ* = 4.45 ppm decreased with the appearance of a new resonance at *δ* = 4.40 ppm, which subsequently decreased but more slowly, as shown in Fig. 11a. Upon a detailed comparison of the independently prepared model complexes, the resonance at *δ* = 4.40 ppm is assigned to an *N*-hydroxylethyl ammonium ester flanked by an *N*-hydroxyethyl amide (Fig. 11b). The faster decay of the resonances at *δ* = 4.45 ppm relative to those at *δ* = 4.40 ppm suggests that esters containing β-ammonium substituents react more rapidly than those containing β-amide substituents.

**Fig. 12:**
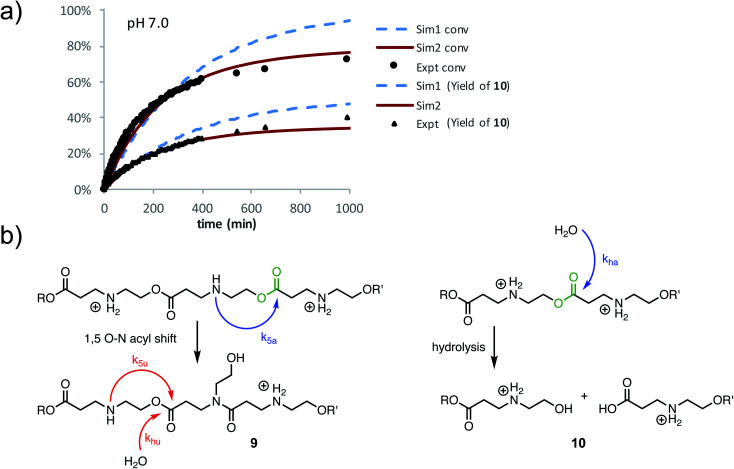
(a) Plot of the percent conversion of hydroxyethyl ester repeat units of **P3+** (black circles) and percent yield of **10** (black triangles). Simulations with optimized rate constants are also plotted: S1 (blue dashed lines, *k*_5_ = *k*_h_ = 1.44 × 10^−3^ min^−1^); S2 (red solid lines, *k*_5a_ = 2.02 × 10^−3^ min^−1^, *k*_5u_ = 6.06 × 10^−5^ min^−1^, *k*_ha_ = 1.62 × 10^−3^ min^−1^, *k*_hu_ = 2.46 × 10^−5^ min^−1^). (b) Scheme of degradation processes and rate constants used in simulations.

At pH 7.0 and 7.5, the total conversion of both types of hydroxyethyl repeat units of **P3+** plateaus at approximately 70% over the course of several hours (Fig. 12a, black circles, Table 2); the appearance of β-amino acids could also be observed (Fig. 12a, black triangles). The complete conversion of the hydroxyethyl units of **P3+** required several days (data not shown).

To simulate these kinetics, two models were evaluated. For the first model (S1), two competitive processes were assumed, one involving a 1,5-O→N acyl shift to generate the linear amide (with rate constant *k*_5_) and a second process involving the hydrolysis of esters (with rate constant *k*_h_, Fig. 12b, dashed blue lines in Fig. 12a). The second model (S2, Fig. 12a) assumed a mechanism incorporating the observation that β-ammonium esters are more reactive both for the 1,5-O→N acyl shift and hydrolysis. For simulation S2, four rate constants were optimized to fit the data (Fig. 12a): *k*_5a_ and *k*_5u_ for the rates of the 1,5-O→N acyl shift when the ester is activated or unactivated by the β-substituent (ammonium or amide respectively), and *k*_ha_ and *k*_hu_ for the similarly defined rates of hydrolysis. Optimization over these parameters was conducted to simultaneously fit the experimental curves of conversion and hydrolysis *vs.* time for pH 7.0 (Fig. 12a, solid red lines).

As shown in Fig. 12a, simulation S2, which accounts for different reactivities for the two types of esters, yielded better fits to the data. Simulation S1, with a single rate constant each for the O→N acyl shift and hydrolysis, would lead to >90% conversion of the polymer repeat units after 1000 minutes, whereas S2 captures both the shape of the kinetics curves throughout and the plateau in conversion at the end. Overall, S2 is consistent with the suggestion by ^1^H NMR (Fig. 11a) that the esters containing β-ammonium groups are more reactive than those containing β-amide groups.

**Fig. 13:**
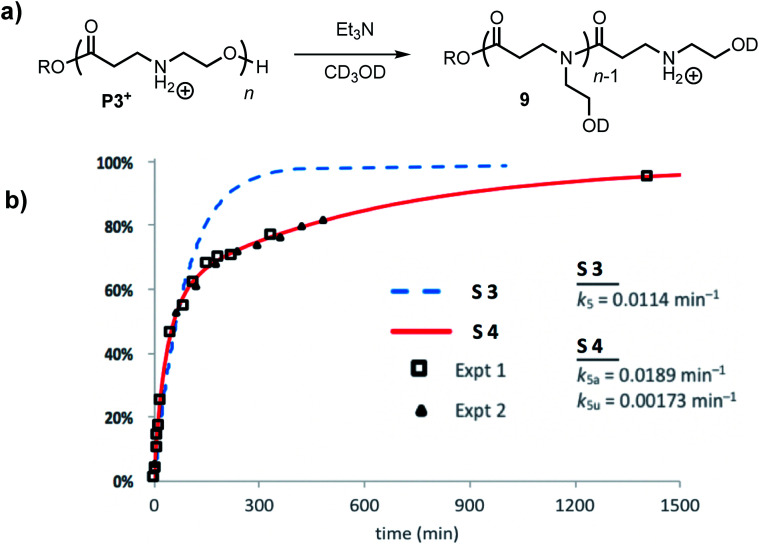
(a) Scheme of degradation processes of **P3+** in Et_3_N/CD_3_OD. (b) Experimental (triangle and squares are experimental replicates) and simulated data (S1 = dashed blue, S2 = solid red) for the percent conversion of repeat units of **P3+** in CD_3_OD with 2.5 equiv. of Et_3_N.

The degradation of **P3+** in CD_3_OD in the presence of 2.5 eq. Et_3_N selectively yields the linear polyamides **9** without competing hydrolysis (Fig. 13a). The conversion of hydroxyethyl repeat units in **P3+** is shown in Fig. 13b. Attempts to model the disappearance of the hydroxyethyl repeat units with a single rate constant *k*_5_ for the O→N acyl shift (model S3, blue dashed line, Fig. 13b) did not reproduce the data well, but the simulated fits incorporating two rate constants *k*_5a_ and *k*_5u_ (defined as above, Fig. 12b) provide excellent agreement with the experimental data for two separate sets of experiments (model S4 red line; exptl. data, black squares and triangles, Fig. 13b). These data provide additional evidence that esters containing β-ammonium groups are more reactive than those containing β-amide groups.

### *N*-Glycyl substituted poly(α-amidoester) **P4+**

The aqueous degradation of **P4+** affords a final mixture of the diketopiperazine **11** and the *N*-hydroxyethyl diglycine **12** from hydrolysis (eqn (3), Table 2). At pH 5.1, **P4+** degrades at a much slower rate than **P1+** (*t*_1/2_ = 443 min), but the degradation rate increases dramatically with increasing pH. At pH 7.0 **P4+** degrades with a rate comparable to that of **P1+** (Table 2; both have *t*_1/2_ < 3 min). Compared to **P1+**, **P4+** degrades with a higher selectivity for the diketopiperazine; at pH 7.0, **11** accounts for 95% of the products, at pH 6.5, for 87% of the products (Table 2 and Fig. S5a, ESI†). For **P4+**, only one 1,6-O→N acyl shift is required to cleave the chain and liberate diketopiperazine **11**, which likely contributes to the high selectivity for **11**.3
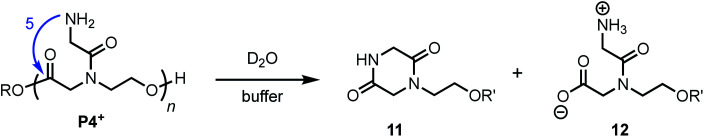


### Controlling the rate of degradation: copolymers

As the rate of degradation of the poly(α-aminoester) **P1+** is considerably faster than that of **P2+** at pH 5.1 (Table 2), we investigated whether the rate of degradation could be tuned by generating random copolymers from aminoester repeat units that exhibit different rates of degradation. To that end, monomers **1** and **2** were copolymerized to generate a series of random copolymers (*M*_n_ = 5.5–10.4 kDa, *Đ* = 1.22–1.31, see ESI†), which upon deprotection, afforded the cationic α-aminoester copolymers with 35%, 54%, and 80% **P2+** content by mole fraction. Because of the fast degradation rates of **P1+** and **P2+** at pH 6.5 (*t*_1/2_ = 3.8 min and 7.6 min, respectively), the kinetic studies were carried out at pH 5.1. The conversion values were calculated relative to the sum of both components, and the empirical half-life as a function of % **P2+** is plotted in Fig. S6 (ESI† p. S17).

As shown in Table 2, the empirical half-life of *N*-hydroxyethylglycine **P1+** homopolymer degradation is approximately 30 minutes at pH 5.1, whereas the empirical half-life for *N*-hydroxyethylalanine homopolymer **P2+** is approximately 80 minutes. By incorporating increasing amounts of **P2+** into the copolymers, the rate of degradation of the copolymers could be tuned as a function of composition, as indicated by the increase of the empirical half-lives (*t*_1/2_) as the mol% of hydroxyethyl alanine repeat units (**P2+**) in the PαAE copolymers increased (Fig. S6†).

### Model systems to assess influence of structure on rate and mechanism of degradation

**Fig. 14:**
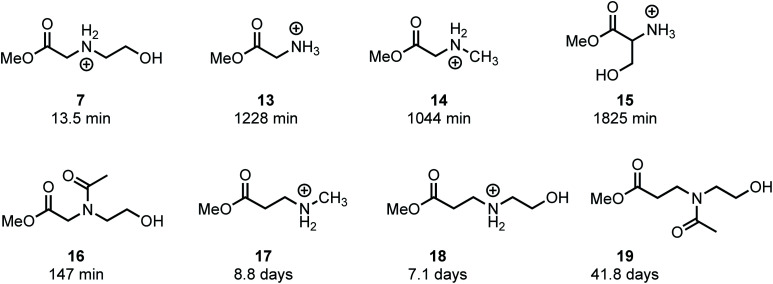
Model methyl esters and their first-order half-lives of hydrolysis at pH 7.0 in D_2_O.

In an effort to provide further insight on the role of polymer structure on the rate of degradation, model studies were carried out with a series of methyl esters that were chosen to mimic the structures of polymer repeat units or intermediates that were implicated in the degradation mechanism. The role of appropriately positioned ammonium and hydroxyethyl groups on the rates of hydrolysis for a series of methyl esters were carried out in buffered D_2_O at pH 7.0. Under these conditions, hydrolysis of the methyl esters afforded the corresponding acids with high selectivity following first order kinetics; thus, the first order half-lives provide a convenient measure for assessing the role of structure on ester reactivity toward hydrolysis (Fig. 14).

As shown in Fig. 14, *N*-hydroxyethylglycinate **7** hydrolyzes most rapidly with a half-life *t*_1/2_ = 13.5 min at pH 7.0 in buffered D_2_O. Methyl glycinate **13**, methyl sarcosinate **14**, and methyl serinate **15** hydrolyze more slowly (*t*_1/2_ = 1228, 1044, and 1825 min, respectively), but considerably faster than the *N*-methyl β-aminopropionate **17** (*t*_1/2_ = 8.8 days).

The faster hydrolysis rate for α- *vs.* β-ammonium esters (**7***vs.***18**, **13–15***vs.***18**, Fig. 14) is consistent with prior studies on the hydrolysis rates of α-amino esters^59–64^ and esters with quaternary ammonium substituents.^65,66^ Esters containing alkyl sulfides in the alpha position also hydrolyze more rapidly than those substituted in the beta position.^67^ Nevertheless, the faster rate of hydrolysis of the β-ammonium esters **17** and **18** (*t*_1/2_ = 7–9 days) relative to the β-amido ester **19** (*t*_1/2_ = 42 days) reveals that β-ammonium substituents exhibit a stronger activating effect on ester hydrolysis than β-amides. Several mechanisms have been proposed to rationalize these effects, including inductive H-bonding and electrostatic activation of the ester by the α-ammonium group.^61,64^ Our results provide a clear rationale for the much faster rate of degradation of the poly(α-ammonium ester)s **P1+** and **P2+** relative to the poly(β-ammonium ester) **P3+** (Table 2).

The much faster rate of hydrolysis of *N*-hydroxyethyl glycinate **7** (*t*_1/2_ = 13.5 min) relative to glycinate **13**, sarcosinate **14**, and serinate **15** (*t*_1/2_ > 1000 min) illustrates the *N*-hydroxyethyl substituent's powerful activating effect on the ester toward hydrolysis. This effect is less pronounced if the *N*-hydroxyethyl group is in the β-position (**17***vs.***18**, *t*_1/2_ = 8.8 *vs.* 7.1 days). The rate of hydrolysis of *N*-acetyl, *N*-hydroxyethyl methyl glycinate **16** (*t*_1/2_ = 147 min) is considerably slower than that for **7**, providing further support for the activating effect of alpha ammonium substituents in activating the ester. Moreover, **16** hydrolyzes much more rapidly than **13** or **14** (*t*_1/2_ = 147 min *vs. t*_1/2_ > 1000 min), again indicating that an appropriately positioned *N*-hydroxyethyl group could activate the adjacent ester for hydrolysis. These phenomena may be a consequence of the neighboring group effect of the pendant hydroxyl group,^68^ as the rate of hydrolysis of **7** is considerably faster than that of **18**.

### Cyclodimerization model system studies

**Fig. 15:**
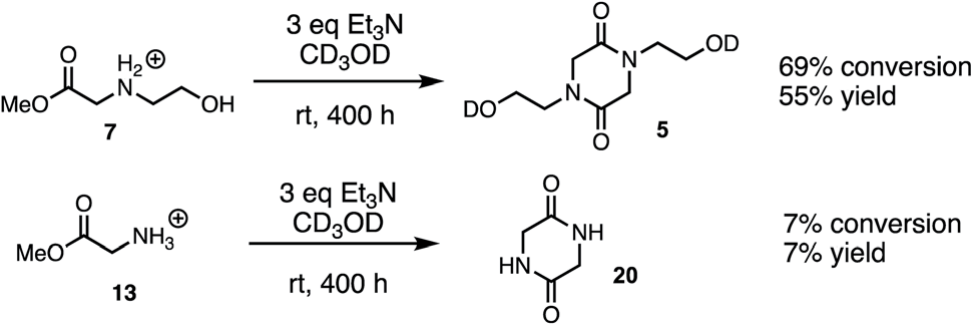
Scheme of cyclodimerization experiments with **7** and **13** in CD_3_OD.

The above studies clearly demonstrate that *N*-hydroxyethyl substituted methyl glycinate **7** is considerably more reactive to hydrolysis than the unsubstituted methyl glycinate **13**. To assess if this relative reactivity was also observed with amine nucleophiles, the methyl glycinates **7** and **13** were each treated with base (Et_3_N) in methanol-*d*_3_ at room temperature. When monitored by ^1^H NMR under these conditions, the only new species observed was the diketopiperazine dimerization product (Fig. 15, see ESI† for other conditions). Both the conversion of the methyl ester **7** and the yield of diketopiperazine **5** were much greater than the analogous quantities for unsubstituted glycinate **13**. These experiments provide additional evidence for the activating effect of the *N*-hydroxyethyl substituent on the reactivity of α-ammonium esters.

## Discussion

We recently discovered a class of environmentally-sensitive novel cationic amphiphilic oligomers (charge altering releasable transporters, CARTs, Fig. 1) that are effective for the delivery of mRNA and pDNA to a variety of cell types in cell culture and live animals.^33,45–49^ These cationic amphiphilic oligomers, when complexed with negatively-charged oligonucleotides, form polyelectrolyte complexes that exhibit dynamic, charge-altering behavior that facilitates the intracellular release of the oligonucleotides.^33^ This dynamic charge-altering behavior was attributed to the unique structural features of the cationic α-aminoester sequences^44^ of these amphiphilic oligomers which exhibit a pH-dependent selective degradation to neutral diketopiperazines.^33^ The studies reported herein reveal that the rate and mechanism of degradation of a series of cationic poly(aminoester) homopolymers in buffered water differ significantly as a function of the structure of the aminoester repeating unit and the solution pH (Fig. 9). These fundamental investigations provide new insights on the factors that influence the degradative charge-neutralizing cationic amine-to-amide/lactam rearrangement which contributes to the charge-altering behavior of the more complex polyelectrolyte assemblies that form when the amphiphilic CART oligomers are complexed with oligonucleotides.

**Fig. 16:**
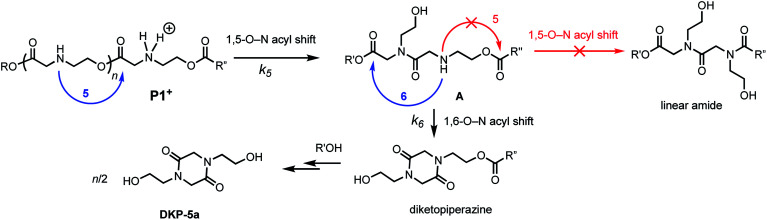
Proposed mechanism of DKP formation; selectivity of 1,6- *vs.* 1,5-O→N acyl shift from intermediate **A**.

Kinetics and mechanistic studies reveal that the poly(α-ammonium ester) **P1+** degrades rapidly and selectively to the diketopiperazine **5** with rates that depend on pH. The base-induced degradation of the cationic **P1+** selectively generates the neutral diketopiperazine **5** as the major product. The cationic **P1+** degrades much more rapidly than polyesters bearing β-ammonium groups (**P3+**) or those lacking an ammonium group (poly(valerolactone)). We had previously proposed that the rapid degradation of the α-ammonium polyester **P1+** was due in part to the activation of the ester carbonyl by inductive and hydrogen-bonding interactions from the vicinal ammonium (Fig. 16).^33^ The proposed 1,5-O→N acyl shift has been reported for related processes in poly(aminoesters) (PAEs) containing side-chain amines.^25,69,70^ The proposed 1,6-O→N acyl shift is analogous to that reported by Almutairi and coworkers for 6- and 7-membered acyl shifts in PAEs featuring side chain nucleophiles.^23,26,27,30^ However, the rapid and highly selective sequence of alternating 1,5- and 1,6-cyclizations to generate diketopiperazine **5** is a novel and characteristic feature for the degradation of the poly(α-amino esters) such as **P1+**. As diketopiperazines are common elimination products in the degradation of peptides,^71^ the structure–activity relationships described herein for O→N acyl shifts may provide insights on related chemistries of biopolymers (*i.e.* O→N or O→S acyl shifts).

The selective degradation of the cationic poly(*N*-hydroxyethyl glycine) polymer **P1+** to liberate the neutral diketopiperazine can be attributed to the propitious positioning of the amine following contraction of the chain by the first 1,5-O→N acyl shift (Fig. 16). Modeling of the kinetics suggests that this selective cyclization cascade occurs randomly along the chain, leaving some of the *N*-hydroxyethyl glycines “stranded”; this is consistent with observations that the degradation of **P1+** yields the diketopiperazine as the major product (≤85%) along with the stranded hydroxyethyl glycinates (≥15%), imposing a stochastic limit (∼85%) on the yield of DKP even under non-aqueous conditions. Model studies (Fig. 14) and literature precedent^59–64^ also indicate that the rapid rate at **P1+** degradation can be in part attributed to the activating effect of the α-ammonium substituent, which predisposes the adjacent ester for nucleophilic attack.^33^ As the pH increases, more of the ammonium groups are deprotonated, facilitating cyclization by a 1,5-O→N acyl shift^25,53,62,64,72,73^ to generate an *N*-hydroxyethylamide intermediate **A** (Fig. 16). The rapid rate of degradation of **P1+** and the faster hydrolysis rate for α- *vs.* β-amino ester model compounds (Fig. 14) are consistent with prior studies on the hydrolysis rates of α-amino esters^59–64^ and esters with quaternary ammonium substituents.^65,66^ The activating effect of the α-ammonium group is a critical factor for the much faster rates of degradation for the poly(α-aminoester)s **P1+** and **P2+** relative to the poly(β-amino ester) **P3+** (Table 2).

As suggested by the kinetic modeling (Fig. 8), the selective degradation of **P1+** to diketopiperazine requires that after the initial 1,5-O→N acyl shift to form **A** (Fig. 16), the subsequent 1,6-O→N acyl shift to yield the diketopiperazine must happen at a rate much faster than the 1,5-O→N acyl shifts that yields linear amides. Literature precedent suggests that for unsubstituted 1,4- and 1,5-aminoesters, the 6-membered cyclization should be slightly favored by 4–7 fold over the 5-membered cyclization.^73^ For the proposed *N*-hydroxyethylamide intermediate **A** (Fig. 16), the planarity of the amide linkage is likely to provide a further bias for 6-membered cyclization, leading to a high selectivity for the diketopiperazine. The structural factors that favor the rapid 6-membered cyclization of intermediate **A** (Fig. 16) can also explain the rapid and selective degradation of **P4+**, which bears a structural motif similar to that of intermediate **A**, as its repeating monomer unit (eqn (3)).

**Fig. 17:**
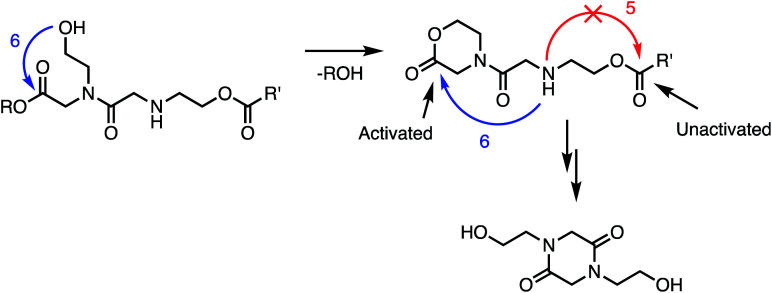
Proposed role of *N*-hydroxyethyl substituents in facilitating the 1,6-O→N acyl shift: neighboring group participation by intramolecular lactonization.

In addition to the activating effect of the α-ammonium substituent, the observation from model studies that *N*-hydroxyethylglycine esters hydrolyze and dimerize much more rapidly than the corresponding glycinates or sarcosinates (Fig. 14 and 15) indicates that an appropriately-positioned *N*-hydroxylethyl group is a key structural element that influences both the rate and selectivity of the degradation of these polymers. The *N*-hydroxyethyl substituent may exert its effect through neighboring group participation (Fig. 17), as has been suggested for analogous reactions of peptides.^74–79^

The significant activating effect of the α-ammonium group is in part responsible for the rapid rate of degradation of the poly(α-ammonium ester)s **P1+** and **P2+**; this effect likely contributes to the rapid rate of degradation of the related poly(serine esters)^25,47,69,80–83^ and poly(hydroxyproline esters).^24^ In contrast to **P1+**, the aqueous degradation of **P2+** occurs exclusively by hydrolysis to give the *N*-hydroxyethylalanine as a zwitterion. Both the faster degradation rate of **P1+** and the different degradation products than that of the *N*-hydroxyethylalanine polymer **P2+** can be explained by the steric effect of the α-methyl substituent that disfavors the 1,5-O→N acyl shift. The kinetics of degradation are also different; kinetic modeling of the evolution of the *N*-hydroxyethylalanine as a function of time (Fig. 10a) indicates that hydrolysis occurs preferentially at the *N*-hydroxylethyl chain-end (Fig. 10b), which correlates well with the activating effect implicated for the *N*-hydroxyethyl substituents on the reactivity of esters bearing that functional group.

The degradation of the β-ammonium polyester **P3+** is both slower and yields different products than either of the α-ammonium polyesters **P1+** and **P2+**. The aqueous degradation of the β-ammonium polyester **P3+** yields the linear amide poly-β-peptoids as the major products along with minor amounts of the *N*-hydroxyethyl esters (eqn (2)). The kinetics, simulations, and model studies (Fig. 14) suggest that esters bearing α-ammonium substituents are more reactive than those bearing β-ammonium substituents, and that esters containing β-ammonium groups are more reactive than those containing β-amide groups. The latter observation could be incorporated into a kinetic model to rationalize the marked deceleration in the rate of degradation of **P3+** over time (Fig. 12a); as the β-ammonium esters are converted to β-amido esters by 1,5-O→N acyl shifts, the resulting β-amido esters react more slowly.

The aqueous degradation of **P4+** liberates the unsubstituted diketopiperazine **11** with higher selectivity (95%, pH 7.0) than **P1+**, due to the positioning of the pendant amine that requires only one cyclization event to cleave the chain. In contrast, the α-methyl substituted polymer **P2+** degrades selectively by hydrolysis, likely as a consequence of steric constraints that disfavor a 1,5-O→N acyl shift.

## Conclusions

This work demonstrates that water-soluble cationic poly(aminoester)s bearing appropriately positioned α-ammonium groups and *N*-hydroxyethyl substituents degrade rapidly and selectively in aqueous solution as functions of structure and pH. The cationic poly(α-amino ester) **P1+** is stable in acidic aqueous solutions, but at a pH where some of the ammonium groups are deprotonated, this polymer degrades rapidly and selectively to the diketopiperazine **5** as a consequence of a facile cyclization cascade mediated by successive 1,5- and 1,6-O→N acyl shifts. Mechanistic and comparative studies with related poly(aminoester)s reveal the key role of both the α-ammonium and *N*-hydroxyethyl substituents on both the rate and mechanism of these selective degradation reactions. These insights have provided a series of cationic water-soluble polyesters whose rates of degradation can be tuned from minutes to hours, depending on the pH.

In related studies, we have demonstrated that this pH-induced degradative transformation of cationic polymers to neutral small molecules provides a strategy to electrostatically bind and release polyanions, such as mRNA and DNA. Oligomers containing short repeat segments of degradable α-amino esters and lipophilic domains constitute a class of charge-altering releasable transporters (CARTs) for the delivery and release of polyanionic genes in cells and live animals.^33,45–49^ The studies reported herein provide key structural and mechanistic understandings on how current CARTs function and inform the criteria to design new pH sensitive polymers for diagnostic and therapeutic delivery. More generally, these studies provide a structural and mechanistic basis for the design of self-immolative polymers or linkers for applications where appropriate environmental triggers can lead to selective degradation, degradation product amplification, or payload release.^1–3,5,8,16^

## Abbreviations

mRNA: Messenger RNA
pDNA: Plasmid DNA
CARTs: Charge-altering releasable transporters
OROP: Organocatalytic ring-opening polymerization
GPC: Gel-permeation chromatography
PAEs: Poly(aminoester)s

## Conflicts of interest

There are no conflicts to declare.

## Supplementary Material

SC-011-C9SC05267D-s001

SC-011-C9SC05267D-s002
