# Supplementary material for: Synthesis and mechanistic investigations of pH-responsive cationic poly(aminoester)s
Source: Chem Sci. 2020 Feb 20;11(11):2951–66. doi: 10.1039/c9sc05267d (PMC8157522; doi:10.1039/c9sc05267d)
Supplement: SC-011-C9SC05267D-s001 [file SC-011-C9SC05267D-s001.pdf]

Electronic Supplementary Material (ESI) for Chemical Science.
This journal is © The Royal Society of Chemistry 2020

Synthesis and Mechanistic Investigations of pH-Responsive Cationic Poly(aminoester)s

Timothy R. Blake†, Wilson C. Ho†, Christopher R. Turlington, Xiaoyu Zang, Melanie A. Huttner, Paul A. Wender, and Robert M. Waymouth*

Department of Chemistry, Stanford University, 450 Serra Mall, Stanford, CA 94305, USA

Materials and methods **S2**
Synthesis of monomers **S2**
Synthesis of homopolymers **S4**
Synthesis of copolymers **S5**
Block copolymers **S5**
Random copolymers of 1 and 2 .... **S5**
Synthesis of cationic polymers **S6**
Synthesis of model compounds for hydrolysis **S7**
Synthesis of polymer degradation products **S8**
Homodimerization experiments to form DKPs **S10**
Degradation kinetics **S11**
Measuring buffer pH **S11**
Representative procedures **S12**
Details of analysis **S12**
Characterization of the P3⁺ polymer rearrangement product **S17**
Stochastic modeling **S20**
General information **S20**
NMR spectra of selected compounds **S23**
Monomers and their synthetic intermediates **S23**
Homopolymers **S26**
Copolymers **S32**
Other compounds **S37**
X-Ray Data For DKP **S48**
References **S52**

Materials and methods

Deuterated solvents (D_2O, CD_3OD, $CDCl_3$) were purchased from Cambridge Isotope Laboratories and dried over activated 3 Å molecular sieves and used without further purification. Dialysis bags were purchased from Spectra/Por (3.5 or 1.0 kDa molecular weight cutoff). Solvents used for polymerization experiments (toluene, CH_2Cl_2, and *p*-xylene), were dried over activated 3 Å molecular sieves and used without further purification. Acetonitrile, acetone, hexanes, and ethyl acetate used for the synthesis and purification of diols and morpholinones were used as received. The Pd catalyst [(neoc)Pd(OAc)(OTf)]$_2$ was prepared as previously reported.[1] Thiourea (TU) was prepared as previously reported.[2] DBU was purchased from Sigma-Aldrich and distilled over activated 3 Å molecular sieves. 1-pyrenebutanol was purchased from Sigma-Aldrich and dried under vacuum before use. Trifluoroacetic acid (TFA) was purchased from Sigma-Aldrich and used without purification. The ring-opening polymerization reactions were performed in a glovebox under nitrogen atmosphere and then quenched outside of the glovebox. All other substrates were purchased from Sigma-Aldrich and used without further purification.

^{1}H NMR and ^{13}C NMR spectra were recorded on either an Inova 300 MHz, Mercury 400 MHz, Inova 500 MHz, or Oxford 600 MHz spectrometer. ^{13}C NMR was conducted with ^{1}H-decoupling by default. In $CDCl_3$, CD_3CN, and CD_3OD, the chemical shifts were referenced to the solvent residuals at δ 7.26, 1.94, and 3.31, respectively. In D_2O, if MeCN or sodium *p*-toluenesulfonate (NaOTs) were used as internal standards, their methyl peaks were set to δ 2.06 and 2.38, respectively.

Gel permeation chromatography (GPC) was performed in tetrahydrofuran (THF) at a flow rate of 1.0 mL/min on a Waters chromatograph equipped with three Waters columns (300 mm x 7.8 mm) connected in series. A Viscotek VE 3580 refractive index detector, Viscotek VE3210 UV/vis detector, and Viscotek GPCmax autosampler were employed. The system was calibrated using monodisperse polystyrene standards from Polymer Laboratories. The pH electrode used was a Hanna Instruments HI-2215 calibrated with NIST pH buffers at 4.01, 7.00, and 10.01.

Synthesis of monomers

N-Boc-morpholin-2-one (**1**)[3] was prepared according to literature procedures. Sometimes compounds have two sets of ^{1}H NMR peaks due to amide or carbamate rotamers. When there is ambiguity over whether the second set of peaks arises from an impurity, either an elevated temperature (70 °C) ^{1}H NMR can be acquired to cause the rotamer peaks to coalesce, or the ^{1}H NMR can be acquired again in a different solvent, which usually causes the equilibrium ratio of rotamers to shift.

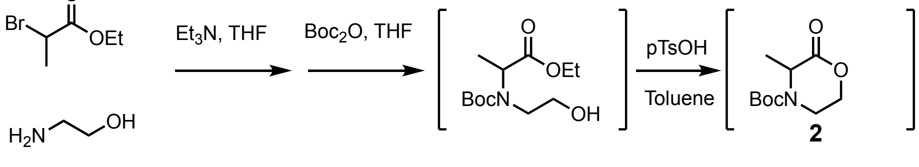

***N*-Boc-3-methyl-morpholin-2-one (2).** Into a flame-dried round-bottom flask cooled in an ice-water bath was added THF, followed by ethanolamine (1.39 mL, 23.0 mmol, 1 eq), and triethylamine (4.8 mL, 34.4 mmol). Ethyl 3-propionate (3.0 mL, 23.1 mmol) was added dropwise into the stirring reaction. The reaction was stirred for 7 h under a nitrogen atmosphere and

warmed up to room temperature. The reaction mixture was then cooled in an ice-water bath to crash out a white precipitate, which was filtered off with a cotton plug. Boc_2O was then added to the filtrate. The Boc_2O dissolved completely upon heating to 60 ˚C in a water bath. After stirring for 20 min, the THF was slowly removed under reduced pressure. The concentrated crude material was redissolved in toluene and mixed with tosylic acid to a concentration of 0.05 M. The toluene solution was then heated to 120-130 ˚C. The toluene and ethanol byproduct were distilled off during heating. The remaining crude material was diluted in toluene, washed three times with saturated aqueous $NaHCO_3$, and dried with $MgSO_4$. After filtering through a fritted glass funnel, the solution was rotoevaporated and purified by silica gel chromatography (40-60% EtOAc/pentane). The purified material was recrystallized in Et_2O (29% yield) and characterized by NMR. ^{1}H NMR (500 MHz, $CDCl_3$): δ 4.67 (s, 1H), 4.37 (m, 2H), 3.86 (m, 1H), 3.40 (m, 1H), 1.48 (d, 3H), 1.49 (s, 9H). ^{13}C NMR (500 MHz, $CDCl_3$): δ 169.65, 153.21, 81.13, 67.73, 52.25, 37.86, 28.26, 18.51.HRMS (ESI in 0.1% formic acid in acetonitrile) *m*/*z*: $[M + H]^+$ Calcd for $C_{10}H_{18}NO_4^+$ 216.1226; Found 216.1230.

***N*-Boc-4-azacaprolactone (3).** At 0 °C, a solution of 3-chloroperoxybenzoic acid (mCPBA; 1.6 g, 7.0 mmol) in 40 mL dichloromethane was added to a solution of *N*-Boc-4-piperidone (1.0 g, 5.0 mmol) in 10 mL dichloromethane. This was warmed to room temperature and stirred for 6.5 h. The reaction was cooled to 0 °C and a second portion of 3-chloroperoxybenzoic acid (0.70 g, 3.1 mmol) was added as a solid. The reaction was warmed to room temperature and stirred overnight. The next day, the reaction mixture was washed three times with saturated aqueous sodium bicarbonate (150 mL total), and then the organic layer was stirred over 600 mg diethylaminomethyl-polystyrene beads (1.9 mmol of base) and 600 mg magnesium sulfate for 1 h. The mixture was filtered and then concentrated *in vacuo*. The resulting white solid was washed on a frit with 100 mL pentane and dried under air, yielding 791 mg (3.7 mmol, 73% yield). ^{1}H NMR (400 MHz, $CDCl_3$): δ 4.24 (m, 2H), 3.76 (m, 2H), 3.65 (m, 2H), 2.80 (m, 2H), 1.47 (s, 9H). ^{13}C NMR (101 MHz, CD_3Cl): δ 173.9, 154.4, 81.1, 69.5, 47.3, 41.3, 37.6, 28.4. HRMS (ESI in 0.1% formic acid in acetonitrile) *m*/*z*: $[M + H]^+$ Calcd for $C_{10}H_{18}NO_4^+$ 216.1226; Found 216.1230.

(*N*-Boc-glycyl) diethanolamine. A flame-dried flask was charged with diethanolamine (225 mg, 2.15 mmol) and methyl (*tert*-butoxycarbonyl)glycinate (370 mg, 1.95 mmol) and then stirred in a heat bath at 75 °C. After 18 hours the reaction was passed through a plug of silica, eluting with acetone. Solvent was removed under reduced pressure yielding 505.1 mg of a pale yellow oil (1.93 mmol, 99% yield). ^{1}H NMR (500 MHz, $CDCl_3$): δ 5.7-5.6 (br, 1H), 3.99 (d, 2H), 3.78-3.65 (m, 4H), 3.53-3.35 (m, 4H), 3.05 (br, 1H). ^{13}C NMR (101 MHz, $CDCl_3$): δ 170.4, 156.3, 79.9, 60.0, 51.1, 42.4, 28.3.

***N*-(*N*-Boc-glycyl) morpholin-2-one (4)**. A solution of *N*-(*N*-Boc-glycyl) diethanolamine (129 mg, 0.492 mmol), 2,2′-bipyridine (3.9 mg, 0.025 mmol), and *N*-methylimidazole (4.1 mg, 0.05 mmol) in MeCN (2.5 mL) was added to a 20 mL vial charged with 9-azabicyclo[3.3.1]nonane *N*-oxyl (ABNO; 2.1 mg, 0.015 mmol), $(MeCN)_4Cu(OTf)$ (9.4 mg, 0.025 mmol), and a stir bar. The reaction solution was stirred under air (vial uncapped) at room temperature for 90 min, during which the solution changed color from red to blue/green. After rotoevaporation, silica gel chromatography was performed (80% EtOAc/hexanes). Concentration

of the relevant fractions yielded a solid white foam (89.7 mg, 0.347 mmol, 71% yield). ^{1}H NMR (500 MHz, $CDCl_3$, rotamers): δ 5.39, 5.35 (br s, 1H), 4.47 (q, J = 4.7 Hz, 2H), 4.40, 4.28 (s, 2H), 3.96 (dd, J = 11.2, 5.0 Hz, 2H), 3.83, 3.70 (t, J = 5.2 Hz, 2H), 1.45 (s, 9H). ^{13}C NMR (125 MHz, $CDCl_3$): δ 167.7, 166.1, 164.8, 155.8, 80.2, 66.9, 65.9, 45.8, 44.1, 42.4, 41.2, 39.3, 28.3, 15.316. HRMS (ESI in 0.1% formic acid in acetonitrile) *m/z*: $[M + H]^+$ Calcd for $C_{11}H_{19}N_2O_5^+$ 259.1285; Found 259.1288.

Synthesis of homopolymers

The degree of polymerization determined by NMR (DP_{NMR}) was calculated by end-group analysis. The 1-pyrenebutyl group has 9 protons in the δ 8.27-7.83 region in $CDCl_3$.

poly(*N*-hydroxyethylglycine) (P1). To a one-dram vial in a glovebox was added 2.1 mg 1-pyrenebutanol (7.7 µmol), 100.6 mg morpholinone **1** (500 µmol), and 400 µL toluene. This monomer/initiator mixture was stirred for 10 minutes to dissolve all solids. To a separate one-dram vial in a glovebox was added 3.8 mg DBU (25 µmol, 5 mol%), 9.3 mg TU (25 µmol, 5 mol%), and 100 µL toluene. This catalyst solution was transferred to the monomer solution via syringe. The reaction mixture was stirred for 6 h in the glovebox at room temperature. The vial was removed from glovebox and the reaction was quenched by adding 2 drops of acetic acid and stirring for 5 min. The crude mixture was dialyzed in methanol overnight (3.5 kDa cutoff). The solvent of the sample in the dialysis bag was removed *in vacuo* the next day, and 71.3 mg of a white powder was isolated (69% yield). ^{1}H NMR (400 MHz; $CDCl_3$): δ 4.30-4.16 (m, 2H), 4.02-3.84 (m, 2H), 3.58-3.44 (m, 2H), 1.50-1.35 (m, 9H). DP_{NMR} = 72. GPC (THF): M_w = 16.2 kDa, M_n = 14.5 kDa, Đ = 1.12.

poly(*N*-hydroxyethyl-DL-alanine) (P2). To a one-dram vial in a glovebox was added 1.4 mg 1-pyrenebutanol (5.1 µmol), 100.8 mg α-Me monomer **2** (469 µmol), and 100 µL toluene. This monomer/initiator mixture was stirred for 10 minutes to dissolve all solids. To a separate one-dram vial in a glovebox was added 4.0 mg DBU (26.3 µmol, 5.6 mol%), 8.9 mg TU (24.1 µmol, 5.1 mol%), and 60 µL toluene. This catalyst solution was transferred to the monomer solution via syringe. The reaction mixture was stirred for 5 h in the glovebox at room temperature. The vial was removed from glovebox and the reaction was quenched by adding 2 drops of acetic acid and stirring for 5 min. The crude mixture was dialyzed in methanol overnight (1.0 kDa cutoff). The solvent of the sample in the dialysis bag was removed *in vacuo* the next day, and 46 mg of a white powder was isolated (46% yield). ^{1}H NMR (500 MHz; $CDCl_3$): δ 4.50-3.31 (m, 8H), 1.50-1.35 (m, 9H). DP_{NMR} = 48. GPC (THF): M_w = 9.4 kDa, M_n = 6.9 kDa, Đ = 1.36.

poly(*N*-hydroxyethyl-β-DL-alanine) (P3). To a one-dram vial under nitrogen were added 1.3 mg 1-pyrenebutanol (4.7 µmol), 100 mg azacaprolactone **3** (460 µmol), 6.0 mg TU (16 µmol), 2.4 µL DBU (16 µmol), and 200 µL toluene. The reaction was stirred overnight. A drop of acetic acid was added the next day to quench the reaction and the solvent was removed. The sample was redissolved in dichloromethane (0.5 mL) and then dialyzed against methanol overnight (3.5 kDa cutoff). The contents of the dialysis bag were concentrated *in vacuo* and 85 mg of a white powder was isolated (84% yield). DP_{NMR} = 75. GPC (THF): M_w = 20.8 kDa, M_n = 18.9 kDa, Đ = 1.10. ^{1}H NMR ($CDCl_3$, 300 MHz): 4.13 (br, 2H), 3.45 (br, 4H), 2.56 (br, 2H), 1.42 (s, 9H). ^{13}C NMR ($CDCl_3$, 500 MHz): 171.9, 171.6, 155.2, 155.0, 80.2, 62.9, 62.6, 46.9, 46.5, 44.3, 44.1, 33.9, 33.4, 28.4.

poly(*N*-glycyl-*N*-hydroxyethylglycine) (P4). To 25.8 mg (0.1 mmol) *N*-glycyl monomer **4** in 50 µl DCM were added together: 0.38 mg (0.0025 mmol) DBU, 0.93 mg TU (0.0025 mmol), and 1.37 mg (0.005 mmol) 1-pyrenebutanol in 50 µl DCM (1.0 M monomer solution). The reaction was stirred for 18 h, then removed from the glovebox and quenched with one drop of acetic acid. The reaction mixture was concentrated *in vacuo* and dialyzed (1.0 kDa cutoff) in DCM against MeOH overnight. Concentration afforded 6.9 mg of a foamy white residue (26% yield). ^{1}H NMR (500 MHz, $CDCl_3$): δ 5.75-5.48 (br, 1H), 4.32-3.43 (m, 8H), 1.45 (s, 9H). DP_{NMR} = 74. GPC (THF): M_w = 2.45 kDa, M_n = 2.0 kDa, Đ = 1.22.

Synthesis of copolymers

In this section, the proton counts in the NMR peak lists are scaled to be per polymer chain (based on end group analysis of the initiator) rather than per repeat unit.

Block copolymers

P(VL$_{49}$-*b*-1$_{74}$). To 100 µL toluene in a one-dram vial, 2.0 mg 1-pyrenebutanol (7.3 µmol), 6.0 mg TU, 2.4 µL DBU (16 µmol, 4 mol%), and 40 µL δ-valerolactone (VL, 430 µmol) were added. The reaction was stirred overnight. The next day, a 50 µL aliquot was removed and quenched with a drop of acetic acid. The solvent was removed *in vacuo* and end group analysis of the sample using ^{1}H NMR ($CDCl_3$, 500 MHz) indicated a degree of polymerization of 47 for poly(valerolactone). The NMR solution was dialyzed in methanol (1 kDa cutoff) for 5 h, and then the contents of the dialysis bag were *in vacuo*. GPC (THF): M_w = 9.7 kDa, M_n = 8.3 kDa, Đ = 1.16.

To the remaining reaction mixture (50 µL), 74 mg of morpholinone **1** (370 µmol) was added to synthesize a valerolactone-morpholinone diblock copolymer. After stirring for 4 h, a drop of acetic acid was added to quench the reaction and chloroform was added until the mixture was fully dissolved. The sample was dialyzed overnight in methanol (3.5 kDa cutoff). The contents of the dialysis bag were *in vacuo* the next day, yielding 75 mg of polymer product (78% yield). ^{1}H NMR (300 MHz, $CDCl_3$) analysis indicated a diblock copolymer with a valerolactone:morpholinone block length of 49:74. GPC (THF): M_w = 17.1 kDa, M_n = 14.2 kDa, Đ = 1.20.

Random copolymers of 1 and 2

P(1$_9$-*r*-12$_{36}$), 80% α-Me. To a one-dram vial in a glovebox were added 0.38 µL benzyl alcohol (3.8 µmol), 7.7 mg α-Me monomer **2** (38 µmol), 50 mg monomer **1** (230 µmol), and 30 µL toluene. The mixture stirred for 10 min. To a separate one-dram vial in the glovebox was added 1.75 mg DBU (11.5 µmol, 5 mol%), 4.3 mg TU (11.5 µmol, 5 mol%), and 10 µL toluene. The catalysts in toluene were transferred to the monomers via syringe. The reaction mixture was stirred for 4 h in the glovebox. ~10 mg benzoic acid was then added to quench the reaction. The sample was dialyzed in methanol overnight (1.0 kDa cutoff). The contents of the dialysis bag were concentrated *in vacuo* the next day, and 32.9 mg of a white powder was isolated (57% yield). GPC (THF): M_w = 7.2 kDa, M_n = 5.5 kDa, Đ = 1.31. ^{1}H NMR (400 MHz, $CDCl_3$): δ 7.34-7.32 (m, 5H), 5.15-5.12 (m, 2H), 4.53-3.27 (m, 234H), 1.60-1.325 (m, 513H). The polymer was determined to contain 80% α-Me content by assigning the multiplet at 1.60-1.325 ppm to 12H x (units of **2**) + 9H x (units of **1**) and assigning the multiplet between 4.53-3.27 ppm to 5H x (units of **2**) + 6H x (units of **1**), and solving for the number of units of **1** and **2** (36 and 9, respectively).

P(1$_{51}$-*r*-2$_{60}$), 54% α-Me. To a one-dram vial in a glovebox were added 2.0 mg 1-pyrenebutanol (7.5 µmol), 45 mg monomer **1** (224 µmol), 120.3 mg α-Me monomer **2** (559

μmol), and 200 μL toluene. To a separate one-dram vial in a glovebox was added 4.3 mg DBU (28 μmol, 5 mol%), 10.3 mg TU (28 μmol, 5 mol%), and 30 μL toluene. The catalysts in toluene were transferred to the monomers via a syringe. The reaction mixture was stirred for 4 h in the glovebox. ~10 mg benzoic acid was then added to quench the reaction. The sample was dialyzed in methanol overnight (1.0 kDa cutoff). The contents of the dialysis bag were concentrated *in vacuo* the next day, and 97.9 mg of a white powder was isolated (59% yield). GPC (THF): M_w = 10.4 kDa, M_n = 8.4 kDa, Đ = 1.24. ^{1}H NMR (400 MHz, $CDCl_3$): δ 8.11-8.01 (m, 9H), 4.54-3.25 (m, 607H), 1.60-1.35 (m, 1180H). The polymer was determined to contain 54% α-Me content by the same method as above (51 units of **1**, 60 units of **2**).

P(1_{44}-*r*-12_{23}), 35% α-Me. To a one-dram vial in a glovebox were added 1.5 mg 1-pyrenebutanol (5.5 μmol), 55.4 mg monomer **1** (276 μmol), 60.1 mg α-Me monomer **2** (279 μmol), and 200 μL toluene. The monomer mixture was stirred for 10 minutes to dissolve. To a separate one-dram vial in a glovebox was added 2.2 mg DBU (14.4 μmol, 5.3 mol%), 5.7 mg TU (15.4 μmol, 5.6 mol%), and 75 μL toluene. The catalysts in toluene were transferred to the monomers via a syringe in one shot. The reaction mixture was stirred for 4 h in the glovebox at room temperature. The reaction vial was removed from glovebox and the reaction was quenched by adding 2 drops of acetic acid. The crude mixture was dialyzed in methanol overnight (3.0 kDa cutoff). The contents of the dialysis bag were concentrated *in vacuo* the next day, and 72.2 mg of a white powder was isolated (50% yield). GPC (THF): M_w = 12.7 kDa, M_n = 10.4 kDa, Đ = 1.22. ^{1}H NMR (400 MHz, $CDCl_3$): δ 8.16-7.85 (m, 9H), 4.50-3.31 (m, 385H), 1.50-1.35 (m, 684H). The polymer was determined to contain 35% α-Me content by the same method as above (44 units of **1**, 23 units of **2**).

Synthesis of cationic polymers

Representative Procedure. Under N_2, the *N*-Boc protected polymer was stirred in 9:1 TFA/DCM (10 mg polymer/mL). After 4-6 h the reaction was concentrated by N_2 stream followed by reduced pressure to afford the cationic polymer as an off-white residue. The cationic polymers may be handled under air at room temperature, but for storage it is recommended that they be stored under N_2 at room temperature, under vacuum (<50 mTorr) at room temperature, or under air at –16 °C. We use the terms deprotected and cationic polymer interchangeably.

Below are the ^{1}H NMR shifts for the cationic polymers in CD_3OD and D_2O. The D_2O is unbuffered, so those solutions are at pH 1-2. Some of the chemical shifts change as the pH is increased from there to pH 5, but further increasing the pH to 7 causes negligible additional change.

P1$^+$

500 MHz, CD_3OD: δ 4.57 (br, 2H), 4.15 (s, 2H), 3.49 (br, 2H)
400 MHz, D_2O: δ 4.59 (m, 2H), 4.19 (s, 2H), 3.56 (m, 2H)

P2$^+$

600 MHz, CD_3OD: δ 4.63 (m, 1H), 4.53 (m, 1H), 4.32 (q, *J* = 7.1 Hz, 1H), 3.53 (br s, 2H), 1.68 (d, *J* = 7.2 Hz, 3H)
600 MHz, D_2O: δ 4.58 (s, 2H), 4.35 (q, *J* = 7.0 Hz, 1H), 3.56 (br, 2H), 1.67 (d, *J* = 6.5 Hz, 3H)

P3$^+$

500 MHz, CD_3OD: δ 4.45 (m, 2H), 3.47-3.36 (m, 4H), 2.92 (t, *J* = 7.0 Hz, 2H)

600 MHz, D_2O: δ 4.46 (m, 2H), 3.46 (m, 2H), 3.43 (t, J = 6.7 Hz, 2H), 2.95 (t, J = 6.7 Hz, 2H)

P4$^+$

400 MHz, CD_3OD: δ 4.42-4.04 (m, 5H), 4.04-3.90 (m, 1H), 3.82-3.62 (m, 2H)
600 MHz, D_2O: δ 4.42-3.93 (m, 6H), 3.80-3.69 (m, 2H)

Synthesis of model compounds for hydrolysis

Methyl 3-(methylamino)propanoate (**16**) was prepared according to literature procedures.[4] For ease of handling and storage, it was then converted to its hydrochloride salt using HCl/Et_2O followed by concentration *in vacuo*.

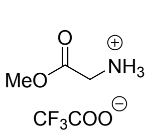

glycine methyl ester (13), trifluoracetic acid adduct. Glycine methyl ester hydrochloride (251 mg, 2.0 mmol) was stirred with diethylaminomethyl-polystyrene beads (1.25 g, 4.0 mmol base) in DCM (15 mL) for 2 h to generate the free base. After filtering and washing the filter cake with DCM (~30 mL), trifluoroacetic acid (230 μL, 3.0 mmol) was added to the filtrate. After stirring for a few minutes, the solution was rotoevaporated, 1:1 Et_2O/THF (~10 mL) was added to the residue, this mixture was filtered, the filter cake was washed with Et_2O (~10 mL), and the filtrate was concentrated *in vacuo*. Very light pink crystals were obtained (190 mg, 0.94 mmol, 47% yield). ^1H NMR (400 MHz, D_2O): δ 3.93 (s, 2H), 3.84 (s, 3H). ^{19}F NMR (400 MHz, D_2O): δ 75.65.

The TFA adducts of the sarcosine and serine methyl esters were made from the commercially available hydrochloride adducts in the same way.

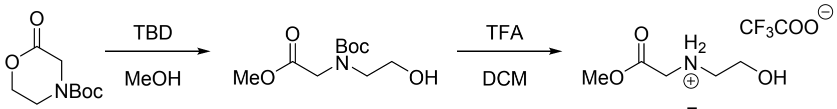

7

***N*-Boc-*N*-(2-hydroxyethyl)glycine methyl ester.** Morpholinone **1** (151 mg, 0.75 mmol) and 1,5,7-triazabicyclo[4.4.0]dec-5-ene (TBD; 3.1 mg, 0.022 mmol) was stirred in dry methanol (5 mL) for 3 h. The reaction was quenched by adding two drops of acetic acid and then rotoevaporated. The residue was purified by silica gel chromatography (50% EtOAc/hexanes). Concentration of the relevant fractions yielded a clear viscous liquid (165 mg, 0.71 mmol, 94% yield). ^1H NMR (400 MHz, $CDCl_3$, rotamers): δ 3.98 (s, 1H), 3.94 (s, 1H), 3.77 (s, 3H), 3.75 (m, 1H), 3.70 (m, 1H), 3.46 (t, J = 4.7 Hz, 1H), 3.43 (t, J = 4.9 Hz, 1H), 3.40, 3.30 (br s, 1H, –O*H*), 1.47, 1.42 (s, 9H).

***N*-(2-hydroxyethyl)glycine methyl ester (7), trifluoracetic acid adduct.** The previous compound (23.1 mg, 0.1 mmol) was stirred in 9:1 DCM/TFA (2 mL; dry, degassed) under N_2 for 2.75 h. Then the solvent was removed by a N_2 stream and the residue was purified by silica gel chromatography (10% MeOH/DCM). Concentration of the relevant fractions yielded a clear viscous liquid (10.4 mg, 0.042 mmol, 42% yield). ^1H NMR (400 MHz, CD_3OD): δ 4.01 (s, 2H), 3.84 (s, 3H), 3.82 (m, 2H), 3.20 (m, 2H). ^{13}C NMR (150 MHz, CD_3OD): 168.12, 57.80, 53.42, 50.60, 48.03. HRMS (ESI in methanol) m/z: $[M + H]^+$ Calcd for $C_5H_{12}NO3^+$ 134.0812; Found 134.0806.

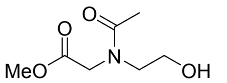

***N*-acetyl-*N*-(2-hydroxyethyl)glycine methyl ester (17).** *N*-acetyl-diethanolamine was oxidized to the morpholin-2-one via the Cu/ABNO method described for **4** (62% yield after SGC with 5% MeOH/DCM). ^{1}H NMR (400 MHz, $CDCl_3$, 1:1 rotamers): δ 4.45 (apparent q, *J* = 5.6 Hz, 2H), 4.39 (s, 1H), 4.32 (s, 1H), 3.81 (m, 1H), 3.73 (m, 1H), 2.13 (apparent d, *J* = 8.0 Hz, 3H).

The morpholin-2-one (108 mg, 0.75 mmol) was ring-opened with methanol and catalytic TBD as described above to give **17** as a viscous yellow oil (119 mg, 90% yield after SGC with 5% MeOH/DCM). ^{1}H NMR (400 MHz, $CDCl_3$, *4*:1 rotamers): δ 4.13, *4.02* (s, 2H), 3.80, *3.78* (s, 3H), 3.77, *3.70* (m, 2H), 3.57, *3.52* (m, 2H), *2.21*, 2.06 (s, 3H). HRMS (ESI in methanol) *m*/*z*: $[M + H]^+$ Calcd for $C_7H_{14}NO_4^+$ 176.0917; Found 176.0912.

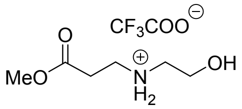

methyl 3-(2-hydroxyethylamino)propanoate (18), trifluoracetic acid adduct. The azacaprolactone monomer **3** was ring-opened with methanol and catalytic TBD (85% yield after SGC with 65% EtOAc/hexanes) and then deprotected with TFA/DCM as described above to obtain a viscous yellow oil. ^{1}H NMR (600 MHz, D_2O): δ 3.83 (m, 2H), 3.73 (s, 3H), 3.37 (br m, 2H), 3.21 (br m, 2H), 2.85 (m, 2H). HRMS (ESI in methanol) of the Boc-protected intermediate *m*/*z*: $[M + H]^+$ Calcd for $C_{11}H_{22}NO_5^+$ 248.1492; Found 248.1485.

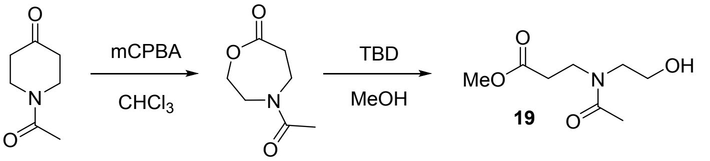

methyl *N*-acetyl-3-(2-hydroxyethylamino)propanoate (19). A solution of *N*-acetyl-4-piperidone (494 mg, 3.50 mmol, 1 eq) in $CHCl_3$ (7 mL) was cooled to 0 °C, to which was added a solution of mCPBA (1.21 g, 4.90 mmol, 1.4 eq) in $CHCl_3$ (28 mL) dropwise over 50 min. The ice bath was removed while stirring continued. After 5 h, more mCPBA (0.6 eq, 0.52 g) was added and the temperature was increased to 45 °C (remaining at room temperature may have been preferable). After an additional 9.5 h, the reaction mixture was cooled to room temperature, concentrated by rotoevaporation, and then stirred with 20 mL of aqueous 1 M $Na_2S_2O_3$ to reduce the unreacted mCPBA. The heterogeneous mixture was extracted with $CHCl_3$ (3 x 25 mL), washed with saturated aqueous $NaHCO_3$ (3 x 25 mL), dried over $MgSO_4$, filtered, and concentrated *in vacuo*. *N*-Acetyl-4-azacaprolactone was obtained as an off-white waxy solid (158 mg, 29% yield). This sample has significant impurities and the ^{1}H NMR spectrum is complex.

The crude azacaprolactone (53.0 mg, 0.34 mmol) was ring-opened with methanol and catalytic TBD as described above to obtain **19** as a light brown oil (44.3 mg, 85% yield after SGC with 2.5-5% MeOH/DCM). ^{1}H NMR (400 MHz, $CDCl_3$, rotamers): δ 3.77 (apparent br q, *J* = 4.9 Hz, 2H), 3.71, 3.67 (s, 3H), 3.66, 3.62 (t, *J* = 6.8 Hz, 2H), 3.50 (m, 2H), 2.64 (m, 2H), 2.16, 2.13 (s, 3H). HRMS (ESI in methanol) *m*/*z*: $[M + H]^+$ Calcd for $C_8H_{16}NO_4^+$ 190.1074; Found 190.1068.

Synthesis of polymer degradation products

Many of the following compounds were generated *in situ* from the hydrolysis or polymer kinetics studies and were not isolated.

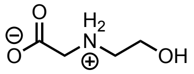

***N*-(2-hydroxyethyl)glycine (6a).** Generated from methyl ester **7** in a hydrolysis study at pH 7.0. Its ^{1}H NMR spectrum matches the literature.[5] ^{1}H NMR (600 MHz, D_2O, pH 7.0 buffered): δ 3.84 (m, 2H), 3.64 (s, 2H), 3.19 (m, 2H).

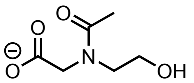

***N*-acetyl-*N*-(2-hydroxyethyl)glycine.** Generated from methyl ester **17** in a hydrolysis study at pH 7.0. ^{1}H NMR (600 MHz, D_2O, pH 7.0 buffered, 1:1 rotamers): δ 4.02, 3.91 (s, 2H), 3.74, 3.69 (t, J = 5.5 Hz, 2H), 3.55, 3.50 (t, J = 5.4 Hz, 2H), 2.19, 2.04 (s, 3H). ^{13}C NMR (150 MHz): 177.24, 177.03, 175.58, 175.07, 59.21, 54.09, 52.36, 50.81, 49.85, 49.46, 21.45, 21.21.

1,4-bis(2-hydroxyethyl)piperazine-2,5-dione (5a). P1+ (215 mg, 1 mmol with respect to monomer units) was suspended in 2 mL DCM. Triethylamine (200uL, 1.44 mmol) was added to the suspension. Within 15 minutes the suspension had dissolved resulting in a homogenous light amber solution. This solution was stirred overnight at which point a white solid had precipitated to coat the inside ove the vial. The supernatant was filtered off, washing with DCM, to provide 72 mg (0.36 mmol dimer, 72 % yield). The crystals were dissolved in 0.5 mL MeOH and vapor diffusion recrystallization was performed with acetonitrile to provide 60 mg XRD quality clear crystals. (XRD data/analysis at end of document).

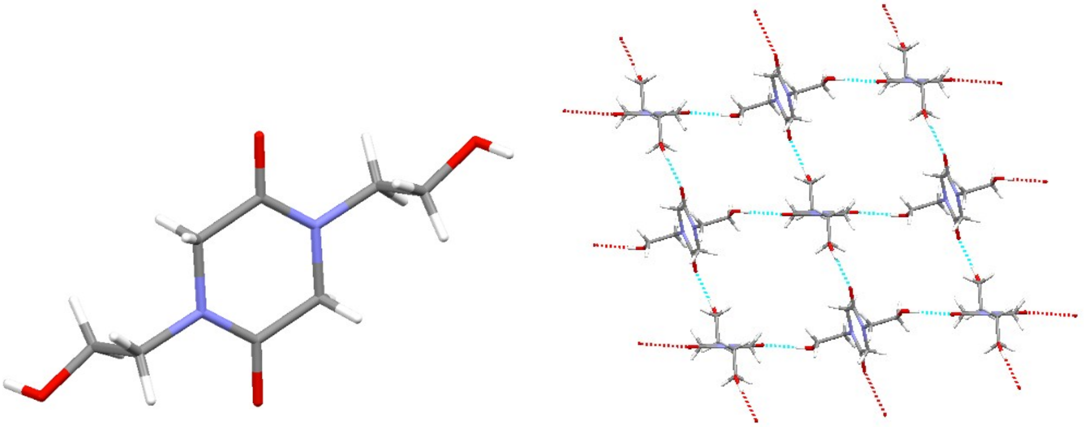

The solid-state structure of **5a** was determined by X-ray crystallography (Figure 3b), which showed a planar diketopiperazine ring. The packing diagram (see Supporting information) reveals significant hydrogen bonding between the hydroxyl protons and amide oxygens, which likely contributes to its high melting point (175 °C) and low solubility in aprotic solvents such as DCM, THF, MeCN, and acetone; **5a** is readily soluble in MeOH and partially soluble in water.

Figure S1. Solid-state **structure of 5a**

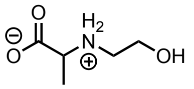

***N*-(2-hydroxyethyl)-DL-alanine (8a).** The α-Me monomer **2** (11.6 mg, 0.054 mmol) was stirred with 1 eq NaOH (2.2 mg) in 1 mL H_2O for 12 h. The solvent was removed *in vacuo* via a MeCN-azeotrope. After thoroughly drying the sample, 1 mL of 9:1 TFA/DCM (dry,

degassed) was added under N_2. After 8 h of stirring, the solvent was removed by a N_2 stream followed by concentration *in vacuo*. The residue was dissolved in D_2O and then adjusted from its starting pH of 1 to pH 5 using 1 M NaOD in D_2O. The ^{1}H and ^{13}C NMR spectra match the literature.[6] ^{1}H NMR (400 MHz, D_2O, pH 5): δ 3.91-3.81 (m, 2H), 3.73 (q, *J* = 7.1 Hz, 1H), 3.19 (t, *J* = 5.2 Hz, 2H), 1.51 (d, *J* = 7.2 Hz, 3H). ^{13}C NMR (150 MHz): 175.37, 58.37, 57.49, 48.36, 15.60.

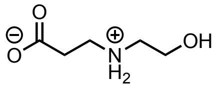

3-(2-hydroxyethylamino)propionic acid (10a). The azacaprolactone monomer **3** (31.0 mg, 0.144 mmol) was stirred in 2 M DCl in D_2O at 70 °C for 12 h. After cooling, a` ^{1}H NMR spectrum of this solution indicated clean, complete reaction. MeCN was added to form an azeotrope, and the solvent was removed *in vacuo* to provide a viscous oil with a slightly green tint. Its ^{1}H NMR spectrum matches the literature.[7] ^{1}H NMR (400 MHz, D_2O): δ 3.85 (m, 2H), 3.25 (t, *J* = 6.9 Hz, 2H), 3.20 (m, 2H), 2.60 (t, *J* = 6.9 Hz, 2H).

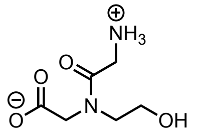

***N*-(2-hydroxyethyl)-*N*-glycylglycine (12a).** The *N*-glycyl monomer **4** (14.3 mg, 0.055 mmol) was stirred in 0.1 M DCl in D_2O for 16 h. ^{1}H NMR indicated full conversion to **12a**. MeCN (~20 mL) was added to form an azeotrope and the solution was concentrated *in vacuo* to give a clear viscous oil. ^{1}H NMR (400 MHz, D_2O, *3*:1 rotamers, pH 1): δ 4.14, *4.07* (s, 2H), *4.02*, 3.81 (s, 2H), *3.60* (t, *J* = 5.1 Hz, 1.5H), 3.57 (t, *J* = 5.5 Hz, 0.5H), 3.43 (t, *J* = 5.4 Hz, 0.5H), *3.37* (t, *J* = 5.1 Hz, 1.5H). HRMS (ESI in Methanol) *m/z*: $[M + H]^+$ Calcd for $C_6H_{13}N_2O_4^+$ 177.0870; Found 177.0865.

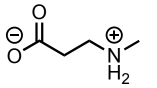

3-(methylamino)propanoic acid. Generated from methyl ester **16** in a hydrolysis study at pH 7.0. Its ^{1}H NMR spectrum matches the literature.[8] ^{1}H NMR (400 MHz, D_2O, pH 7.0 buffered): δ 3.19 (t, *J* = 6.9 Hz, 2H), 2.72 (s, 3H), 2.60 (t, *J* = 6.9 Hz, 2H).

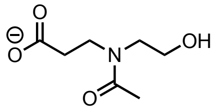

***N*-acetyl-*N*-(2-hydroxyethyl)-3-aminopropanoic acid.** Generated from methyl ester **19** in a hydrolysis study at pH 7.0. This compound is an analog of **9a**. ^{1}H NMR (600 MHz, D_2O, pH 7.0 buffered, rotamers): δ 3.77-3.64 (m, 3H), 3.58-3.47 (m, 3H), 2.48, 2.42 (t, *J* = 7.4 Hz, 2H), 2.16, 2.14 (s, 3H).

Homodimerization experiments to form DKPs

Reaction 1. A solution of methyl ester **7** (5.7 mg, 0.023 mmol, 1 eq) and biphenyl (4.2 mg, 0.027 mmol; NMR internal standard) in dry CD_3OD (0.6 mL) was prepared in an NMR tube and the *t* = 0 spectrum was acquired. Then Et_3N (10.9 µL, 0.078 mmol, 3.4 eq) was added, the tube was inverted multiple times, and the reaction mixture was left to proceed at room temperature. Time points were taken periodically until *t* = 414 h.

Reaction 2. Prepared in the same way as Reaction 1, but at $t = 55$ h the temperature was increased to 50 °C. Time points were taken for another 171 h.

Reaction 3a-b. A solution of glycine methyl ester hydrochloride (**13**, 31.4 mg, 0.25 mmol, 1 eq) and biphenyl (38.6 mg, 0.25 mmol) in dry MeOH (6.5 mL) was prepared in a vial. The $t = 0$ spectrum was acquired after taking a 50 µL of the reaction solution, gently removing the solvent by a N_2 stream, and redissolving it in CD_3OD. The reaction cannot be done in CD_3OD because α-deuteration of the starting material occurs to a large extent over the very long time span of this experiment, which removes the only ^{1}H NMR handle for quantification. Then Et_3N (104.5 µL, 0.075 mmol, 3 eq) was added and the reaction mixture was stirred at room temperature.

At $t = 56$ h, a time point was taken and then the reaction solution was split into two equal parts. Reaction 3a remained at room temperature and time points were taken periodically until $t = 414$ h. Reaction 3b was heated to 50 °C and time points were taken periodically until $t = 227$ h.

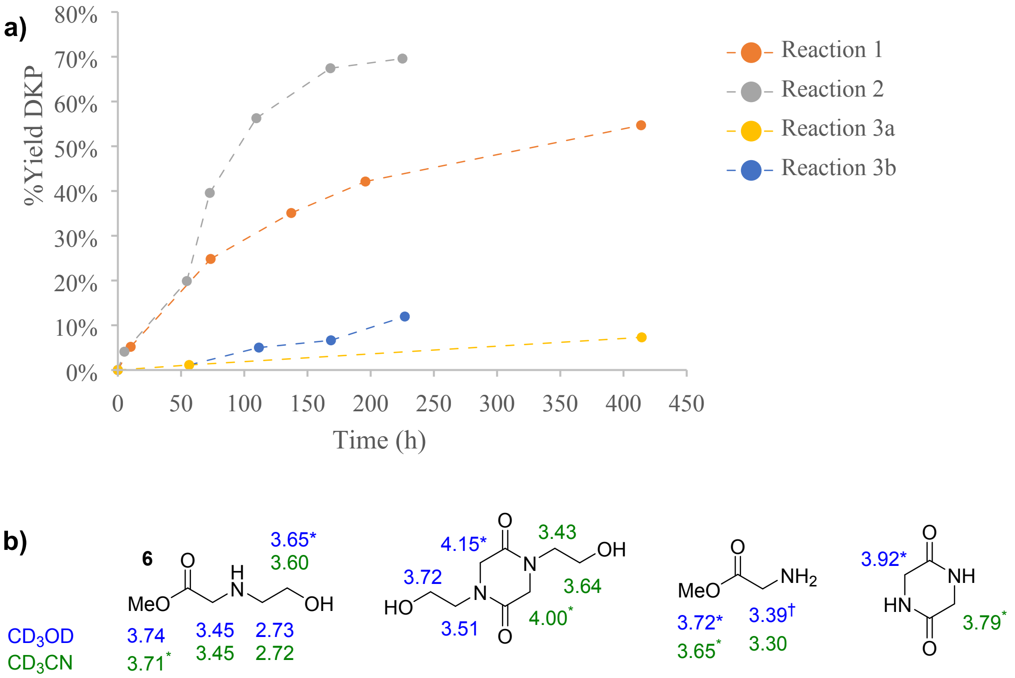

Figure S1. Homodimerization experiments with **7** and **13** in CD_3OD. **a)** DKP yield vs. time. **b)** ^{1}H NMR assignments for the starting materials and DKP products. *Used for quantification. †This chemical shift is variable depending on the pH (zwitterions are drawn as neutral species for clarity).

Degradation kinetics

Measuring buffer pH

The value that is displayed on a pH meter that has been calibrated to pH standards in H_2O when its electrode is dipped into a D_2O solution is called pH*. To associate each D_2O buffer that we prepared with the more familiar pH value, the buffers prepared in D_2O at the concentrations indicated in Table S1 were diluted 20x in distilled H_2O and the pH recorded with a pH meter. Further dilution does not change the reading, suggesting that the effect of D_2O on the reading has been made negligible. All of the buffers remained stable in pH value over time. For the kinetics experiments, the buffers were diluted into an equal volume of D_2O.

Table S1. Buffer compositions in D_2O

pH[a]	Components	Conc. (M)[b]	Conc. (M)[c]	Conc. (M)[d]
5.1	d_4-acetic acid/NaOH	3.2	0.16	1.6
6.5	KH_2PO_4/K_2HPO_4	3.3	0.165	1.65
7.0	KH_2PO_4/K_2HPO_4	3.6	0.18	1.8
7.5	KH_2PO_4/K_2HPO_4	3.6	0.18	1.8
~9[e]	$NaHCO_3$ (satd.)	1.1	0.055	0.55

[a]Measured by pH electrode. [b.] Concentration of buffer, as prepared in D_2O. [c]. Concentration of buffer at which pH measured. [d.] Concentration of buffer for kinetics experiments. [e.] Measured by pH paper.

Representative procedures

The default counteranion is trifluoroacetate. Some of the model systems for hydrolysis were prepared with chloride counteranion, but this does not affect their kinetics. The acquisition time (`at`) and scan delay (`d1`) parameters on the Varian NMR instrument are measured in seconds. `nt` is the number of scans.

Polymer degradation kinetics in D_2O. Deprotected polymer **P1**$^+$ (10.4 mg, 0.048 mmol by repeat units) was dissolved in 0.50 mL D_2O with either 4 µL MeCN (0.077 mmol; internal standard; its methyl peak was set to δ 2.06) or 7 mg NaOTs (0.036 mmol; δ 2.38). The t_0 ^1H NMR spectrum was acquired with `nt = 8`, `at = 8`, and `d1 = 12`, parameters which were confirmed to provide a long enough time between scans to ensure accurate relative integrations between polymers and small molecule products. 0.50 mL of the desired buffer solution in D_2O was then added to the NMR tube, which was rapidly inverted multiple times to ensure thorough mixing and placed back into the spectrometer. The shims were adjusted as quickly as possible (typically <30 s), and then NMR spectra were acquired once every 90 s for 2.5 h using the parameters `nt = 4`, `at = 8`, and `d1 = 12`. The time elapsed between adding the buffer and beginning the acquisition was recorded, which was typically ~2 min. In general, when the polymer degradation rate is or has become slow, the number of scans can be increased for improved signal-to-noise at minimal expense of time resolution. The arrayed spectral data were analyzed using MNova software. The polymer concentration in the total solution in the vast majority of experiments is between 40-60 mM.

Model system hydrolysis studies. The procedure is essentially the same as that used for polymers. A shorter `d1 = 6` can be used here as there are no polymers involved. Some of these reactions are so slow that they are conducted over many days, in which case it is necessary to use NaOTs as the NMR internal standard instead of MeCN for volatility reasons.

Polymer degradation kinetics in CD_3OD. Deprotected **P1**$^+$ polymer (6.3 mg, 0.029 mmol) was dissolved in 0.60 mL CD_3OD with 6 mg biphenyl (0.039 mmol; internal standard; alternatively the solvent residual at δ 3.31 can be used). The t_0 ^1H NMR spectrum was acquired with `nt = 8`, `at = 8`, and `d1 = 12`. Et_3N (8.1 µL, 2.0 eq) was then added to the NMR tube, and the procedure from here onward is the same as that used in the aqueous kinetics.

Details of analysis

The following are representative examples of the ^1H NMR spectra and kinetics data gathered from the aqueous degradations of each of the polymers studied, as well as the detailed peak assignments used to analyze them. Data already presented in the main text typically will not be repeated here.

$P1^+$

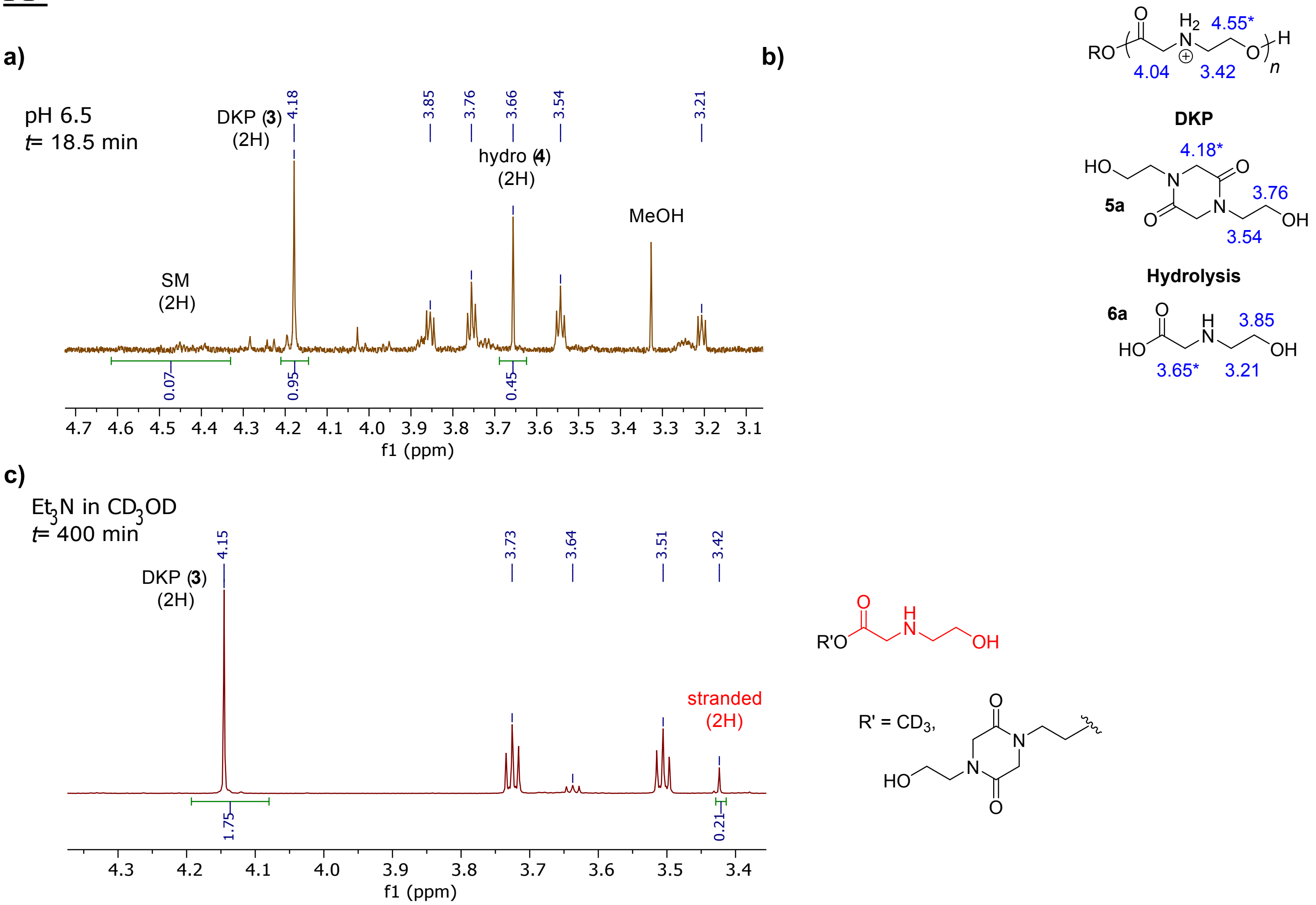

Figure S2. P1⁺ polymer degradation analysis. **a)** ^{1}H NMR spectrum from a degradation at pH 6.5. **b)** ^{1}H NMR peak assignments. *Used for quantification. **c)** ^{1}H NMR spectrum from a degradation using 2.8 eq Et_3N in CD_3OD. The peak assignments in CD_3OD for **5a** and the model compound that mimics stranded repeat units (**7**) were given in Figure S1b (zwitterions are drawn as neutral species for clarity).

The DKP peak at δ 4.18 is treated as a 2H peak (instead of 4H) because two repeat units are consumed to produce each DKP molecule. The excellent mass balance exemplified in Figure S2c (100% conv) demonstrates that the limited DKP yield does not arise from side reactions consuming the starting material.

P2+

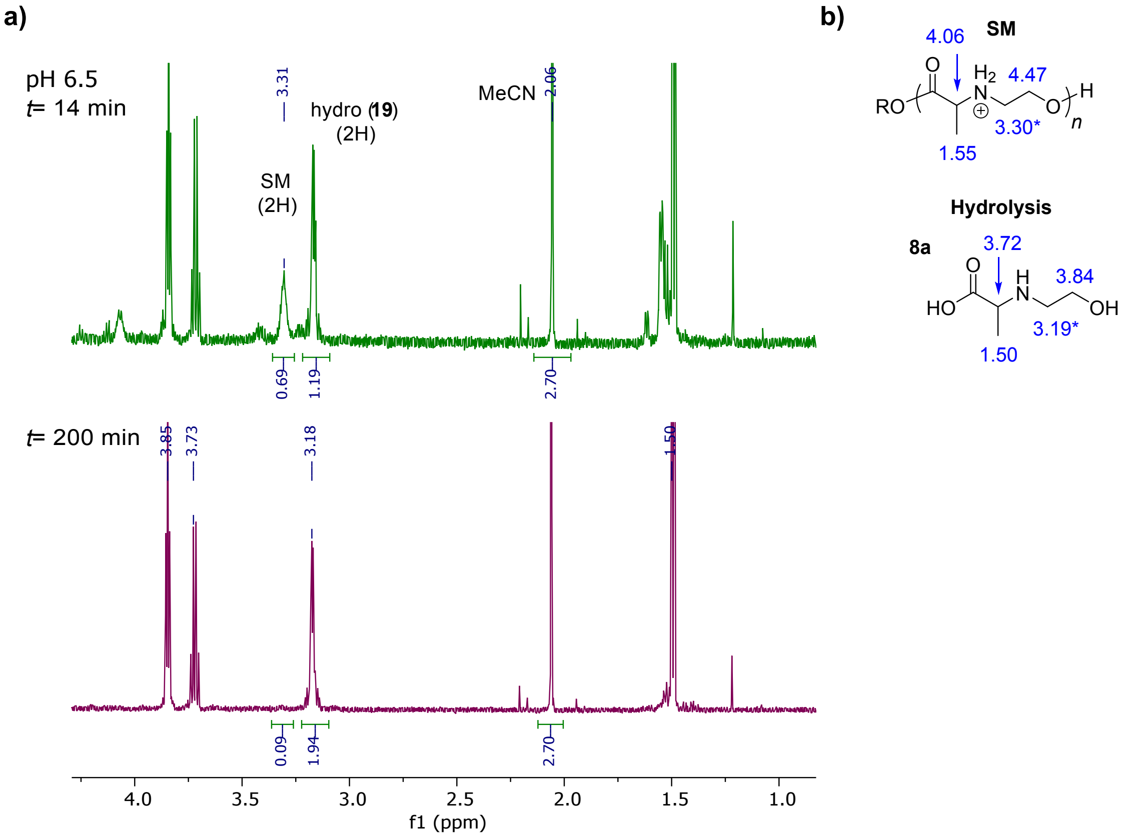

Figure S3. P2+ polymer degradation analysis. **a)** ^{1}H NMR spectra from a degradation at pH 6.5. **b)** ^{1}H NMR peak assignments. *Used for quantification, zwitterions are drawn as neutral species for clarity.

As shown above, the degradation of the α-Me polymer with Et_3N in CD_3OD produces a very complicated ^{1}H NMR spectrum. The two resonances highlighted in the figure stand conspicuously apart and downfield from the others. They have a nearly perfect 3:1 ratio of areas as well. Based on our ^{1}H NMR experience with other DKPs, we have strong reason to believe that these resonances arise from a diketopiperazine. DKP formation is not likely to be relevant under physiological conditions, however, due to hydrolysis being far more facile.

P3+

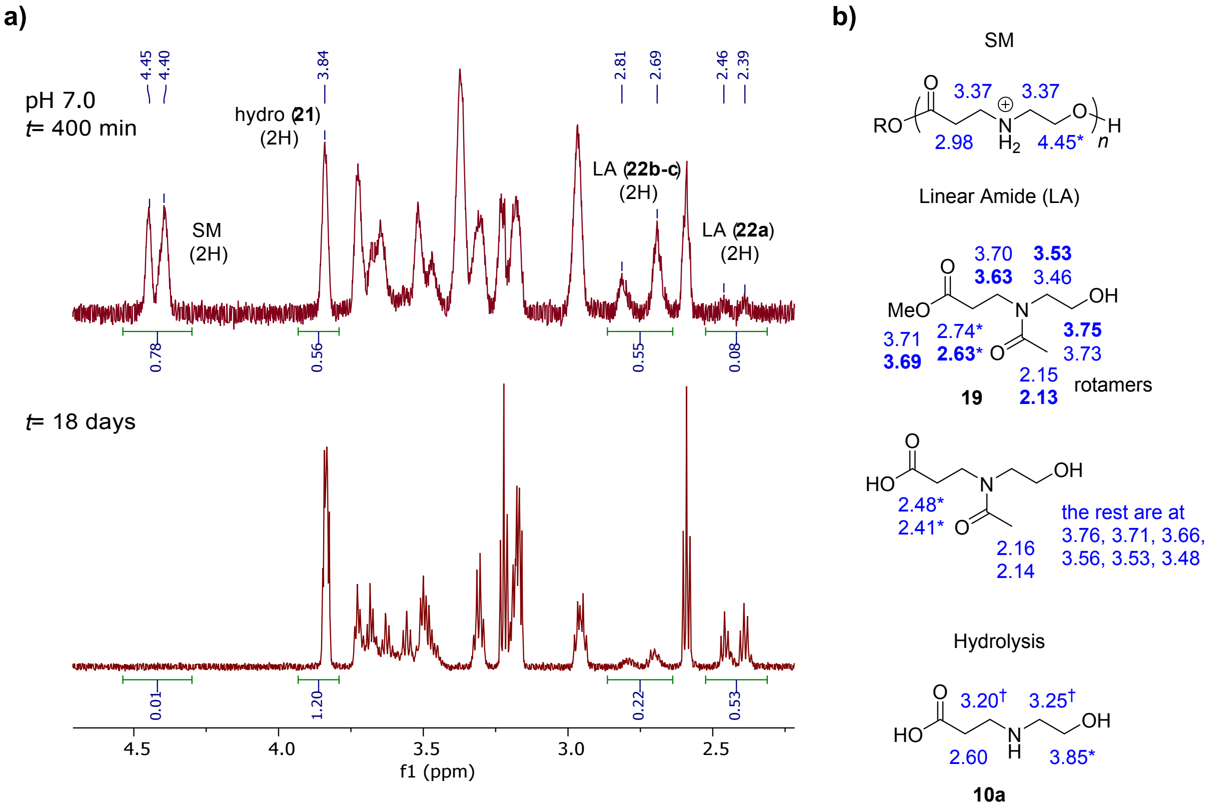

Figure S4. P3⁺ polymer degradation analysis. **a)** ^{1}H NMR spectra from a degradation at pH 6.5. **b)** ^{1}H NMR peak assignments. *Used for quantification. †These assignments are tentative, zwitterions are drawn as neutral species for clarity.

Ester- and/or amide-adjacent linear amides **22b-c** are initially formed in greater amount than carboxylic acid-adjacent linear amide **22a**, but then over many days the area appears to transfer from **22b-c** to **22a**. This makes sense because the ester in **22b** can slowly hydrolyze to give **22a**, but the reverse is not possible. That such a transfer of area is observed supports the validity of our assignments. When degraded in saturated aqueous $NaHCO_3$, the poly(β-aminoester) **P3⁺** undergoes minimal hydrolysis and polyamide **22c** appears to be the nearly exclusive product (see Figure S7).

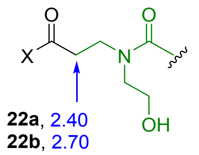

a (X = OH)
b (X = OR)
c (X = NR_2)

R ≠ H

P4⁺

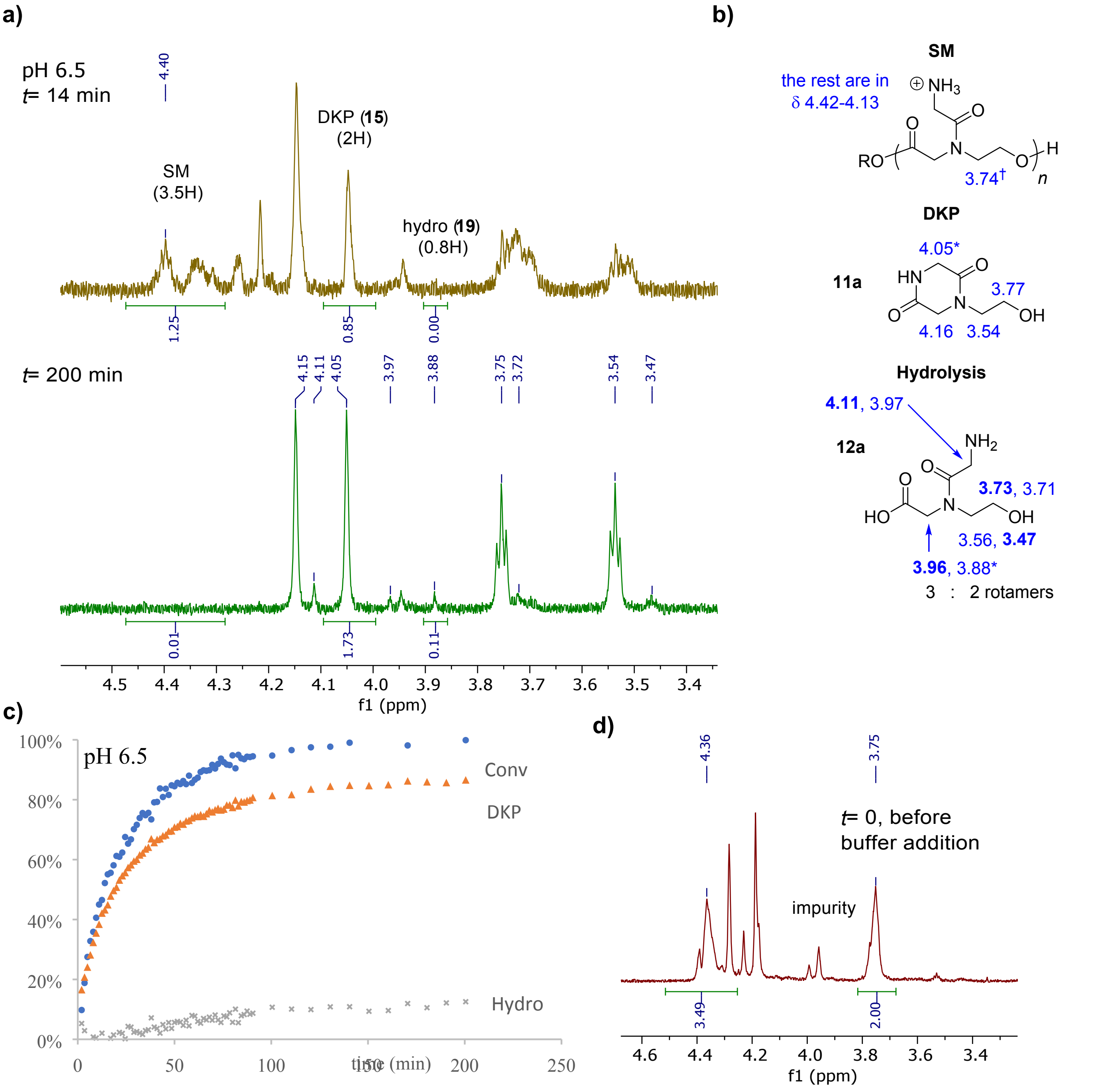

Figure S5. P4⁺ polymer degradation analysis. **a)** ^{1}H NMR spectra from a degradation at pH 6.5. **b)** ^{1}H NMR peak assignments. *Used for quantification. †Used for quantifying the starting amount of polymer, (zwitterions are drawn as neutral species for clarity) but not to keep track of its disappearance over time. **c)** Kinetics graph for this reaction. **d)** Magnified view of the starting material peaks.

The starting material peaks for this polymer are particularly difficult to quantify, in part because the rotamers add complexity to the spectrum. The δ 3.75 (2H) peak that is well resolved at $t = 0$ unfortunately cannot be used as it severely overlaps with one of the DKP resonances. After trying several different integration regions (including sums over two intervals), we believe the above integration choice is not only the best that can be done using simple integration (i.e.,

without peak fitting), but also an accurate measure of conversion. The mass balance is decent, and once the peaks in this region disappear, no further changes are seen in the spectrum.

Copolymers of **1** and **2**

Only conversion was tracked for the degradations of these copolymers since the product distribution was complex, especially considering the possibility of a mixed DKP forming from one repeat unit each of the **1** and **2** types. The total starting material was quantified via the broad peak at δ 3.50-3.38, which corresponds to the same CH_2 position in the polymer backbone for either type.

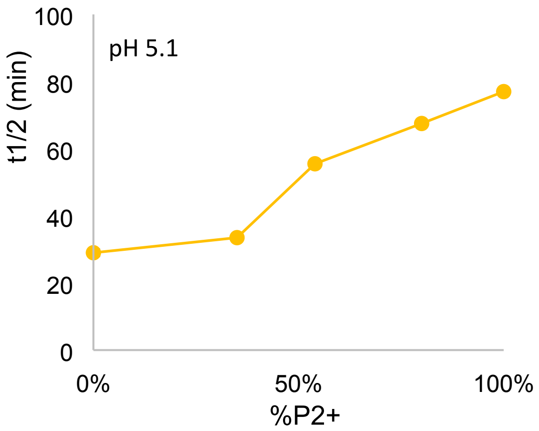

Figure S6. Empirical half-lives for the conversion of monomer repeat units of $P1^+/P2^+$ copolymers at pH 5.1 as a function of mol% of *N*-hydroxyethyl alanine repeat units ($P2^+$) in the chain.

Characterization of the $P3^+$ polymer rearrangement product

Sample isolation

32 mg of **P3**$^+$ was stirred for 30 min in 0.32 mL of saturated aqueous $NaHCO_3$. The solvent was removed by air stream and the sample was placed under high vacuum for 10 min. The sample was redissolved in 0.6 mL methanol and then syringe-filtered. The sample was dried under high vacuum for 48 h, yielding 17 mg solid (99% yield). The sample was then analyzed by ^{1}H NMR, IR spectroscopy, and diffusion spectroscopy (DOSY). ^{1}H NMR (D_2O, 500 MHz): δ 3.8-3.5 (m, 6H), 2.9-2.7 (m, 2H). IR: 1589 (s) cm^{-1}.

^{1}H NMR

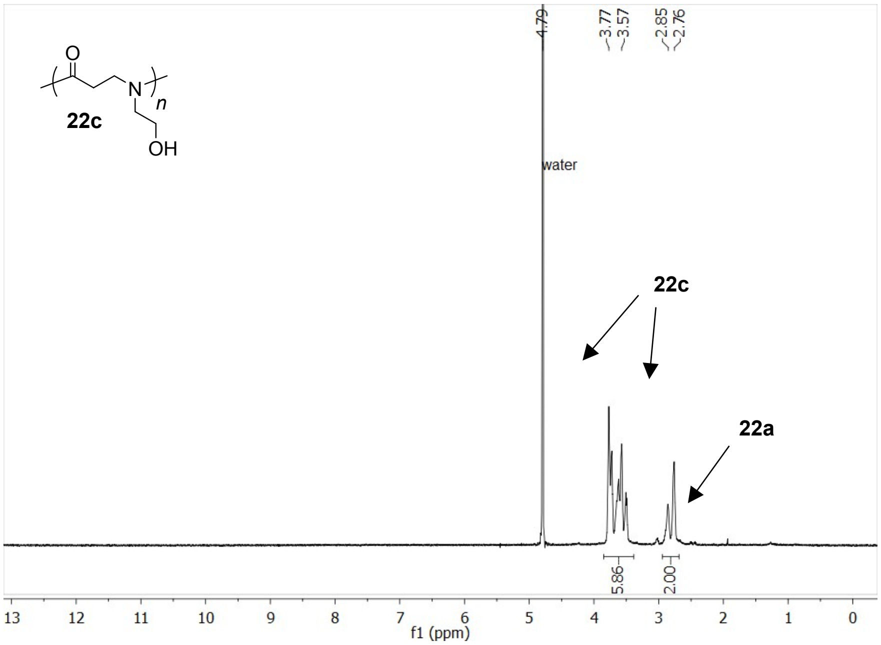

Figure S7. ^{1}H NMR of the degradation products of the **P3⁺** polymer after workup. The integrated peaks correspond to polyamide **22c**. There are no ester-adjacent amides **22b** here as evidenced by the lack of alkoxy peaks.

A similar degradation of **P3**$^+$ was carried out in saturated aqueous $NaHCO_3$ with NaOTs as an internal standard. In that case, the ^{1}H NMR integration over δ 2.90-2.70 gave an 85% yield of the above linear amide rearrangement product. In neither case do we see any of the usual peaks associated with hydrolysis (except for the very small ones corresponding to **22a**) and there are no extra peaks that need to be accounted for.

IR spectroscopy

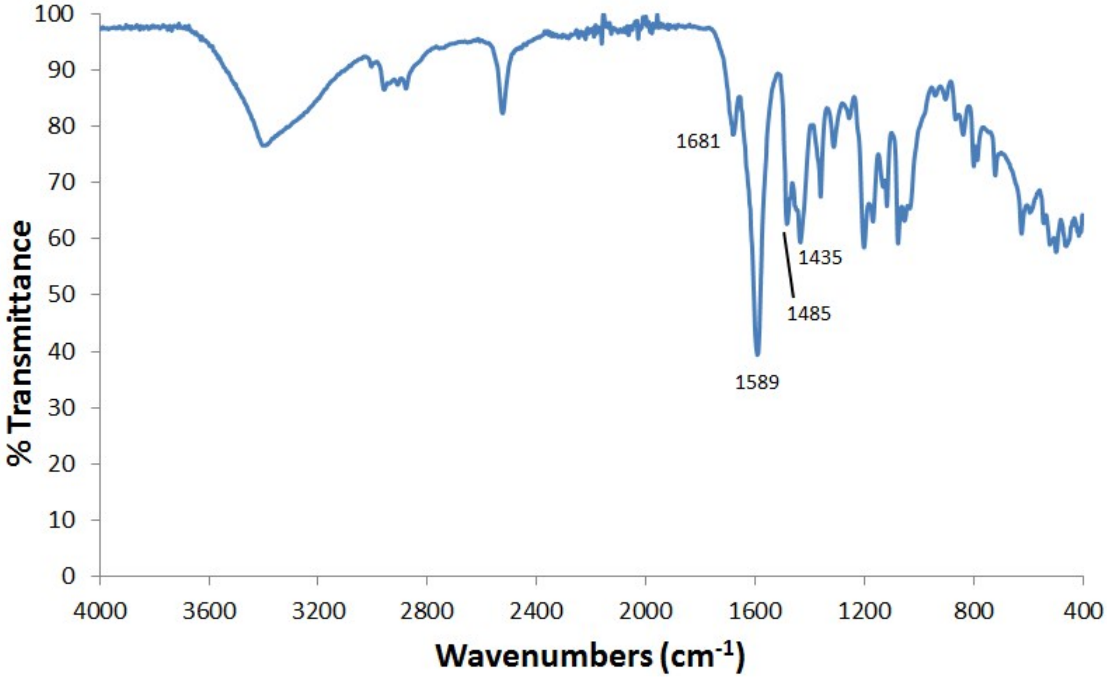

A strong absorption at 1589 cm^{-1} suggests that amide formation has occurred in the polymer backbone.

MALDI-TOF

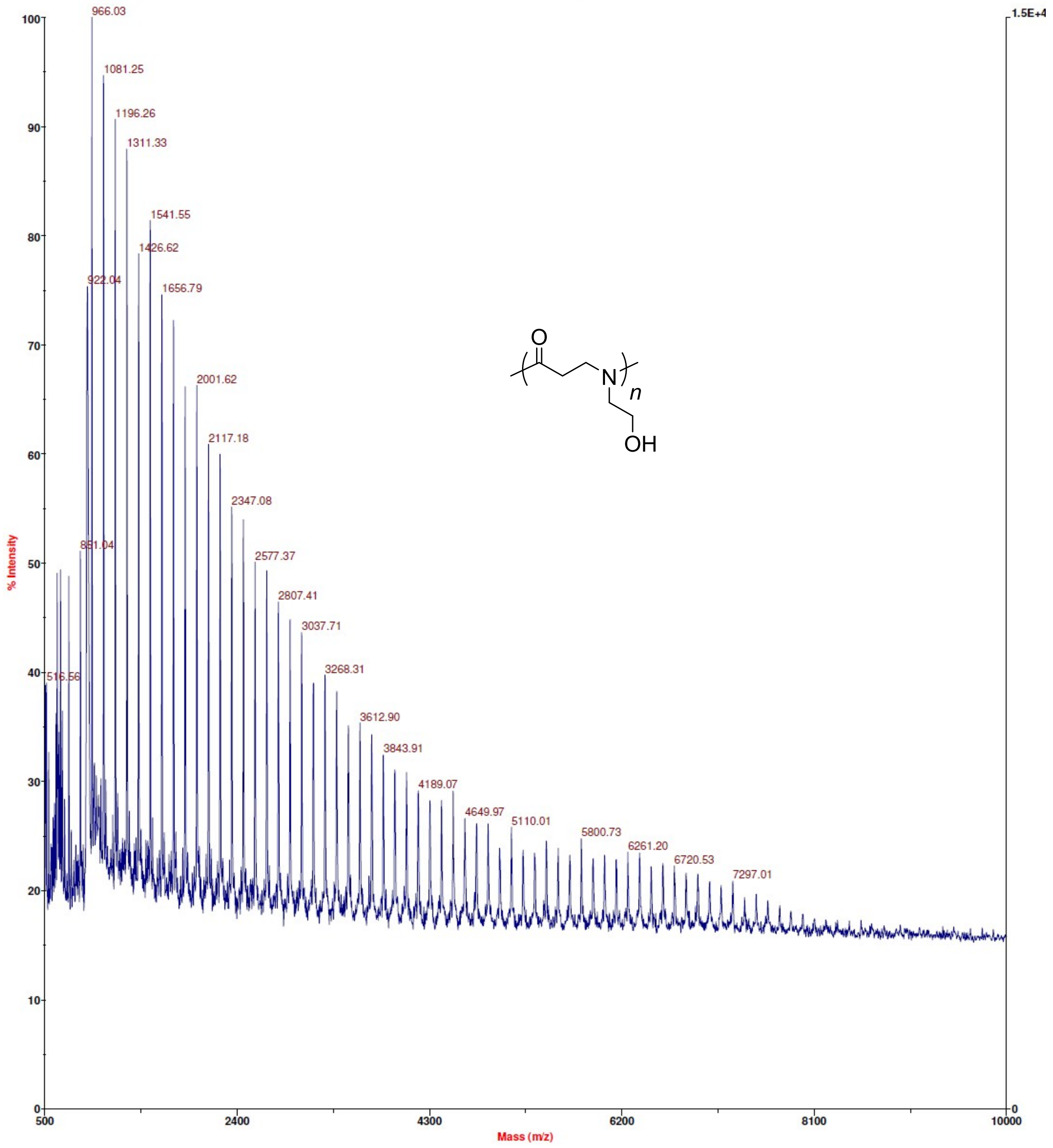

Species observed by MALDI-MS were separated by a mass of 115 Daltons. This mass corresponds to the repeating *N*-hydroxyethylamide unit expected after rearrangement via a 1,5 O→N acyl shift. If no hydrolysis occurred to fragment the polymer chain, then the expected weight of the polyamide product would be ~10 kDa. Species were detected with molecular weights greater than 7 kDa, but most fragments had molecular weights between 1-4 kDa.

Stochastic modeling

General information

All stochastic modeling simulations were carried out in MATLAB (version R2018b). In these simulations, a polymer molecule is represented as a 1D-array of states corresponding to the repeat units of the polymer. Reactions (i.e., O→N acyl shifts and ester hydrolysis) are carried out sequentially on the polymer until no further reactions can take place. This simulation is then repeated (typically 1000 times) with a new polymer molecule each time and the results are averaged. Every reaction is treated as an independent Poisson process and is characterized by a rate k, which has dimensions of time^{-1}. The simulation keeps track of breaks that are formed in the polymer chain (which necessarily creates new chains), but does not include any reactions that occur between different chains.

As noted in the main text, this simulation uses the Gillespie method. Briefly, to perform the next reaction on the polymer, the simulation first makes a list of all possible reactions that could occur; suppose there are m possibilities and $k_1, \ldots, k_m$ are their associated rates. Since they are independent Poisson processes, the process that counts all reaction events of any type is a Poisson process with rate $k_{tot} = k_1 + k_2 + \ldots + k_m$. The waiting time for the first event of any type to occur follows an exponential distribution with rate parameter k_{tot}. A random time increment Δt is generated from this distribution (using MATLAB's `rand` function). Which reaction occurs is then selected according to the fact that the probability of reaction i being that first event is k_i / k_{tot}. The polymer state array is then appropriately modified to reflect that reaction. This process is then repeated until no further reactions can occur. Each iteration of this process is considered a "step" in the simulation.

The following scheme depicts one step occurring on a partially degraded chain of **P3**$^+$ according to the β-amino activation model. Consider the R group to be inert (e.g., 1-pyrenebutyl). The reaction in the dotted green box is the one that has been randomly selected to occur, and the probability of that specific reaction being selected was k_{5a} / ($2k_{5a} + k_{5u} + 3k_{ha} + k_{hu}$).

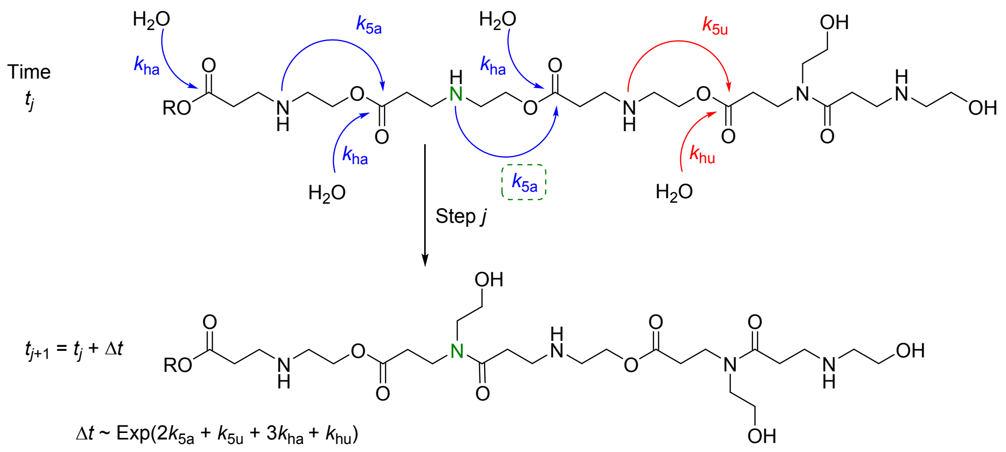

Scheme S1. Illustration of a single step of the stochastic modeling. Exp(λ) is the exponential distribution with rate parameter λ.

Fitting to experimental data

The optimization of k values to fit the experimental data was carried out using MATLAB's `patternsearch` function. `patternsearch` can perform the numerical optimization of a multi-input objective function whose output contains stochastic noise. The objective function quantifies the total error between the experimental and simulated data as the sum of absolute residuals. It is possible to fit multiple curves simultaneously, such as those of conversion and hydrolysis.

The time-axis of the simulated data is scaled such that a particular curve intersects its experimental counterpart at a given value; typically this is set to 50% conversion. The optimizations are always carried out with one of the k values held constant at 1 (arbitrary units) since the time-axis will be multiplicatively scaled in this manner.

In the models where there is only one adjustable k value (Models 1, 2, and 4 below), only time-axis scaling was performed so this is considered a 1-point fitting (to, e.g., the point at 50% conversion); optimization using `patternsearch` is never carried out. In the models with multiple adjustable k values (Models 3, 5, and 6), time-scaling and the `patternsearch` function are employed. This is considered a multi-point fitting because the absolute residual from every experimental data point is taken into account.

Mechanisms modeled

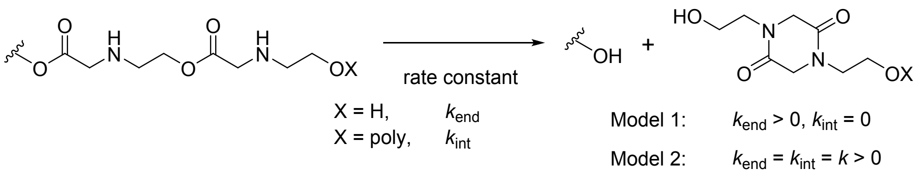

1) **P1**$^+$ polymer with perfectly alternating 1,5-1,6-O→N acyl shifts that occur only at the chain-end (CD_3OD)

2) **P1**$^+$ polymer with perfectly alternating 1,5-1,6-O→N acyl shifts that occur at random (CD_3OD)

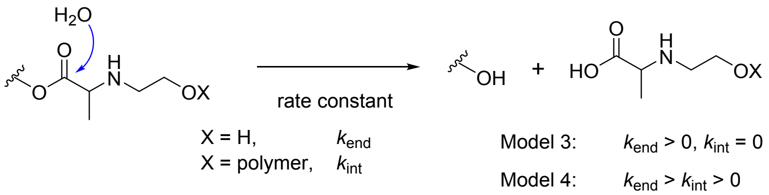

3) **P2**$^+$ polymer with degradation only at chain-ends (D_2O)
4) **P2**$^+$ polymer with chain-end acceleration (D_2O)

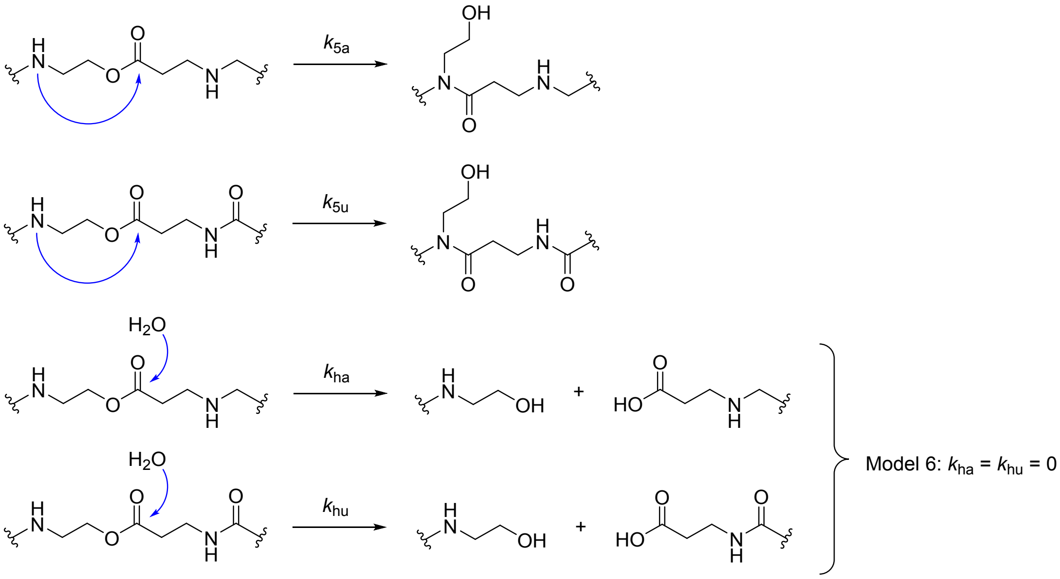

5) **P3**$^+$ polymer with the β-amino activation effect with hydrolysis (D_2O)
6) **P3**$^+$ polymer with the β-amino activation effect without hydrolysis (CD_3OD)

NMR spectra of selected compounds

Monomers and their synthetic intermediates

N-Boc-3-methyl-2-morpholinone (2)

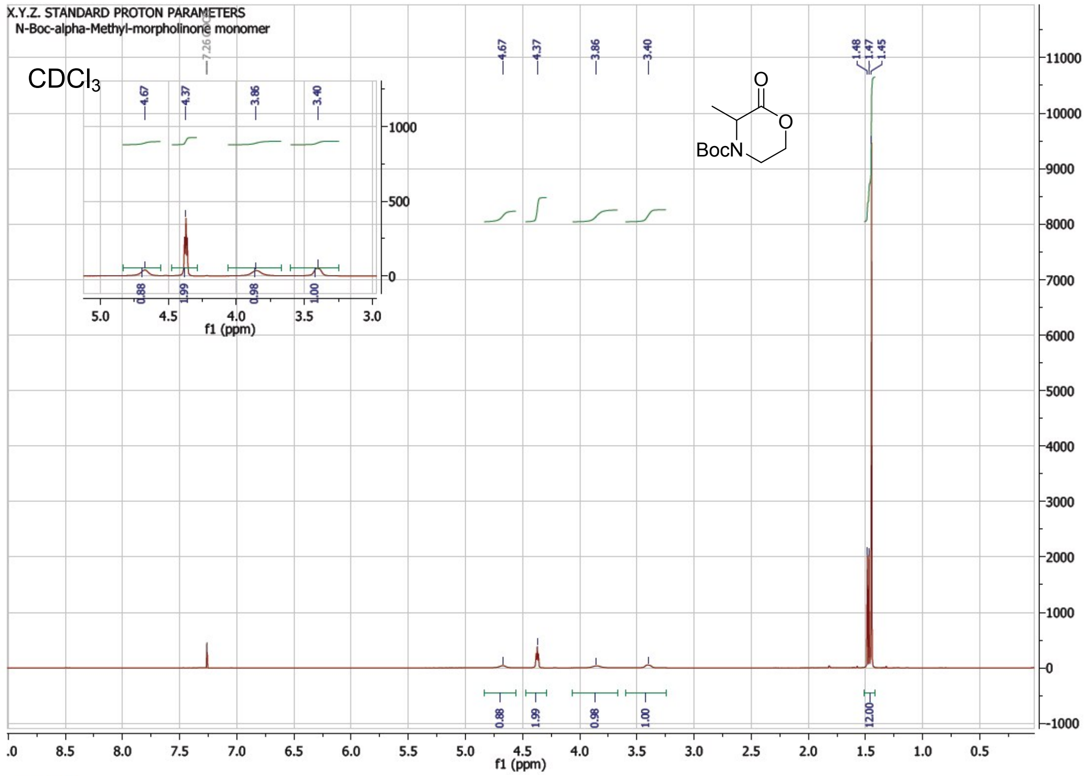

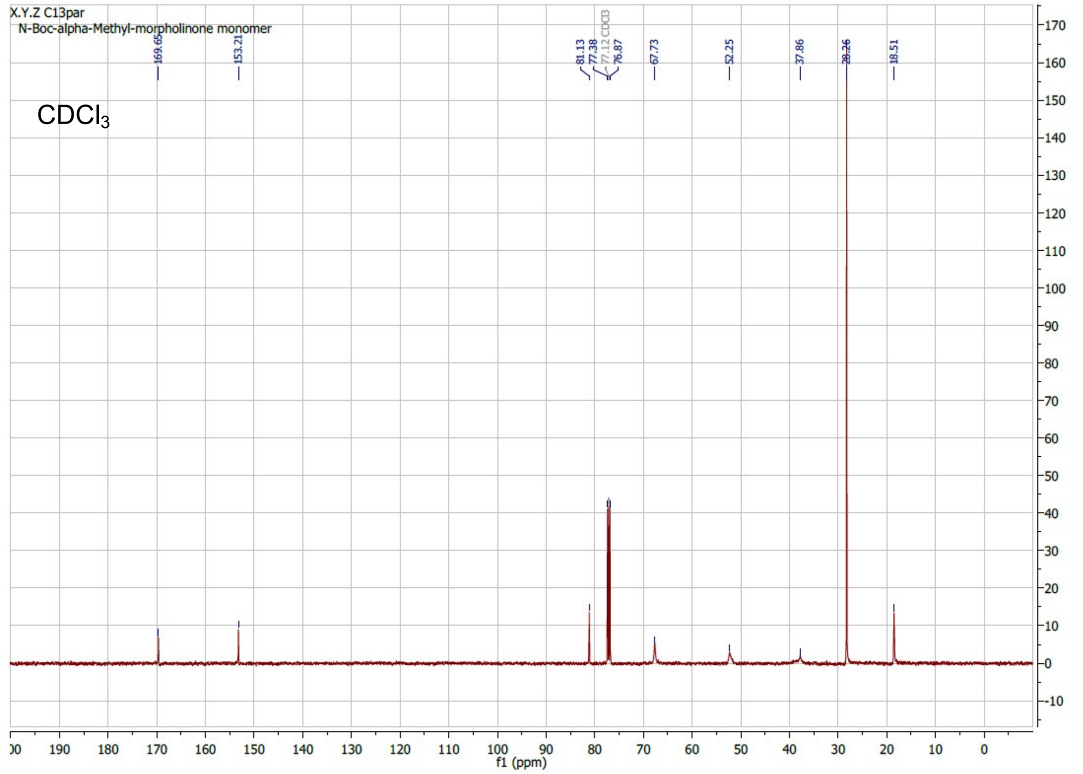

***N*-Boc-4-azacaprolactone (3)**

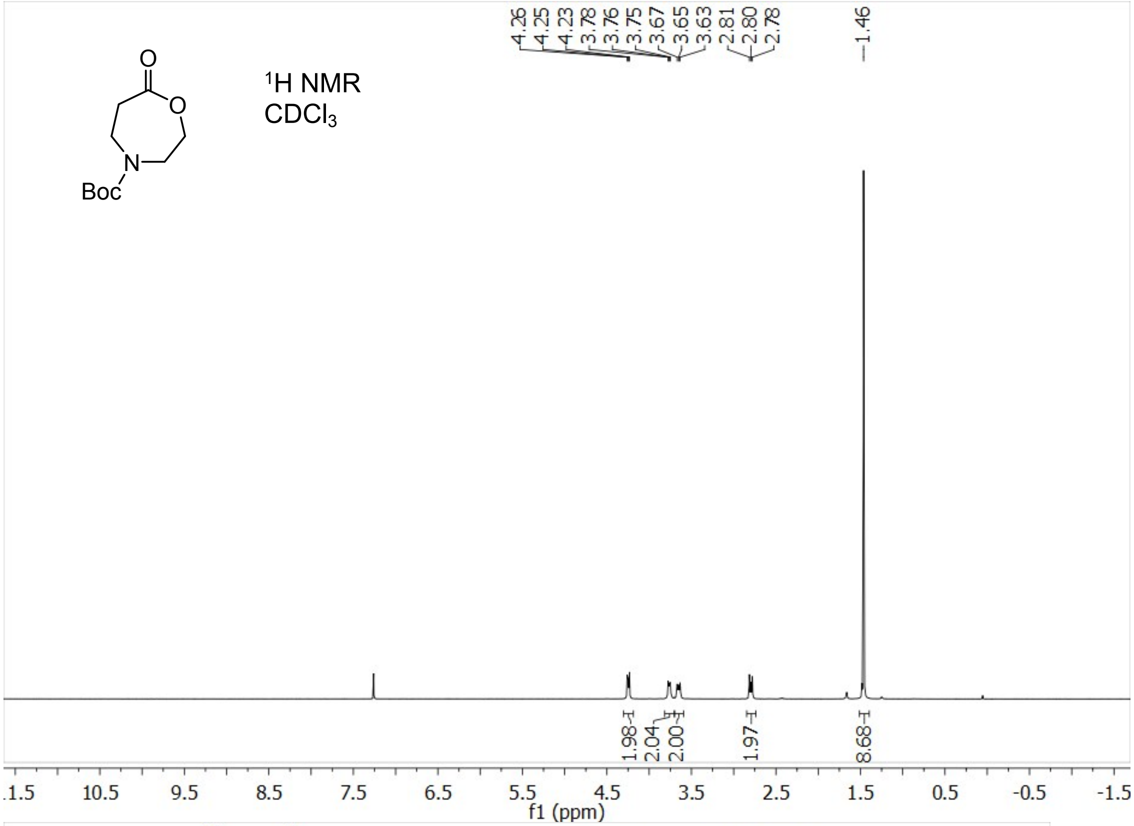

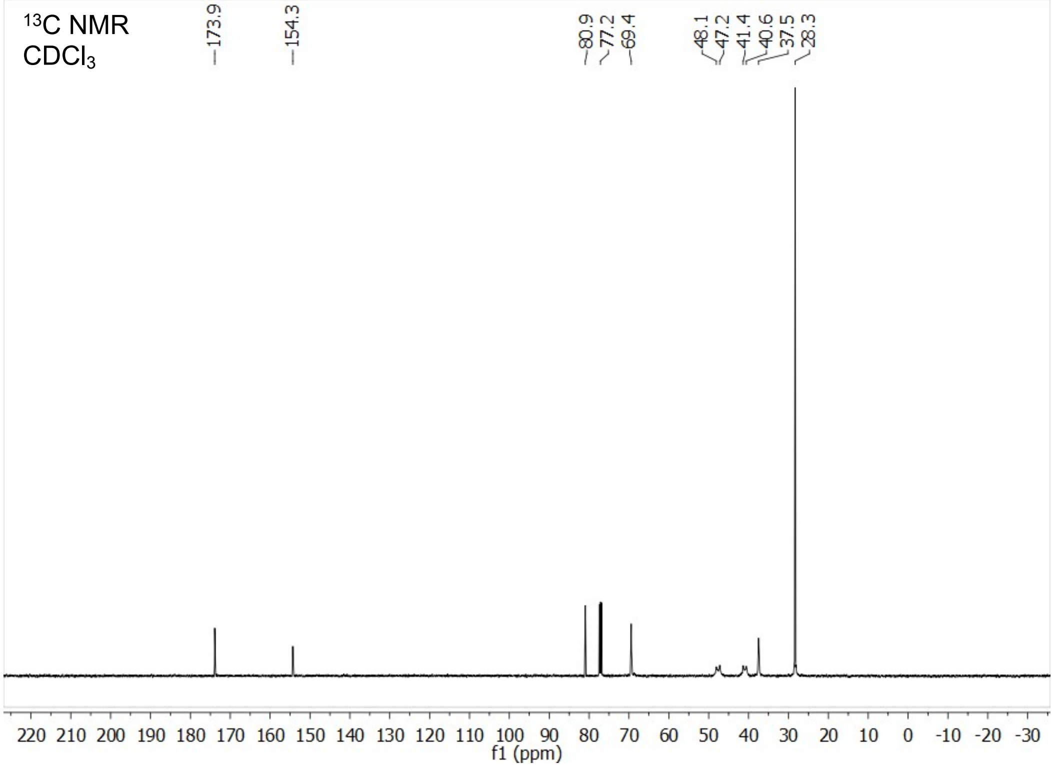

***N*-(*N*-Boc-glycyl) morpholin-2-one (4)**

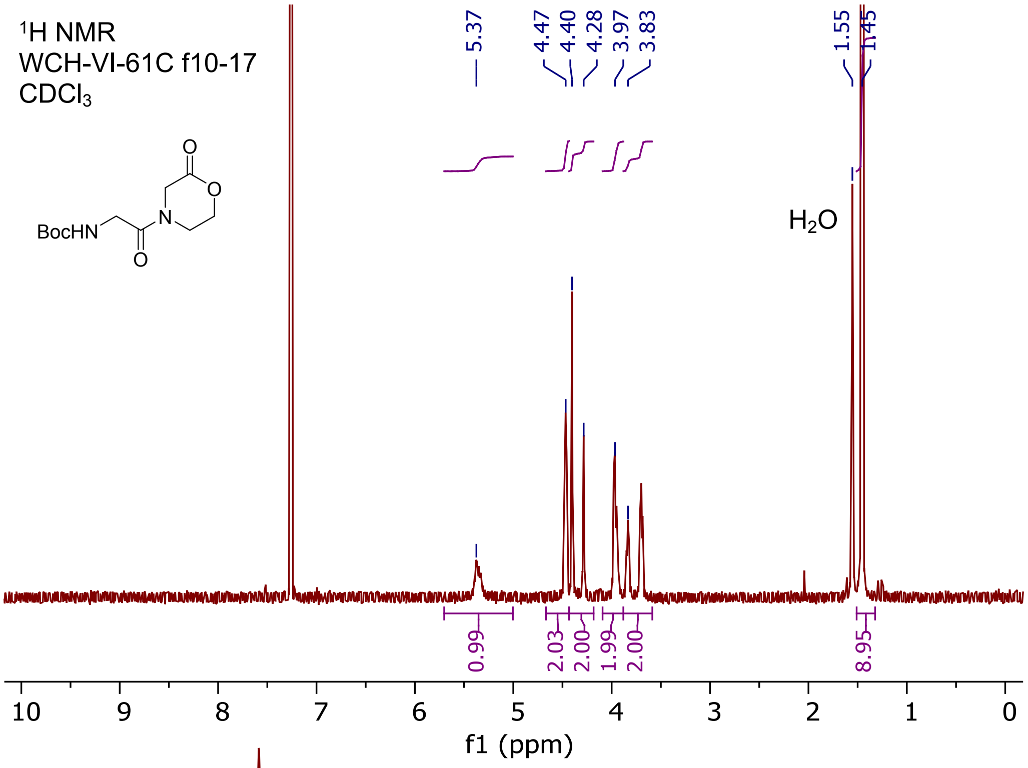

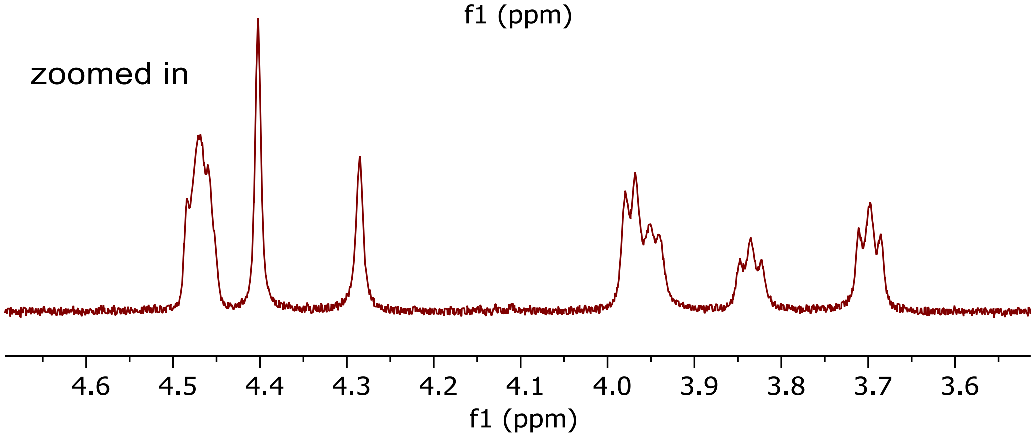

Homopolymers

$P1_{72}$

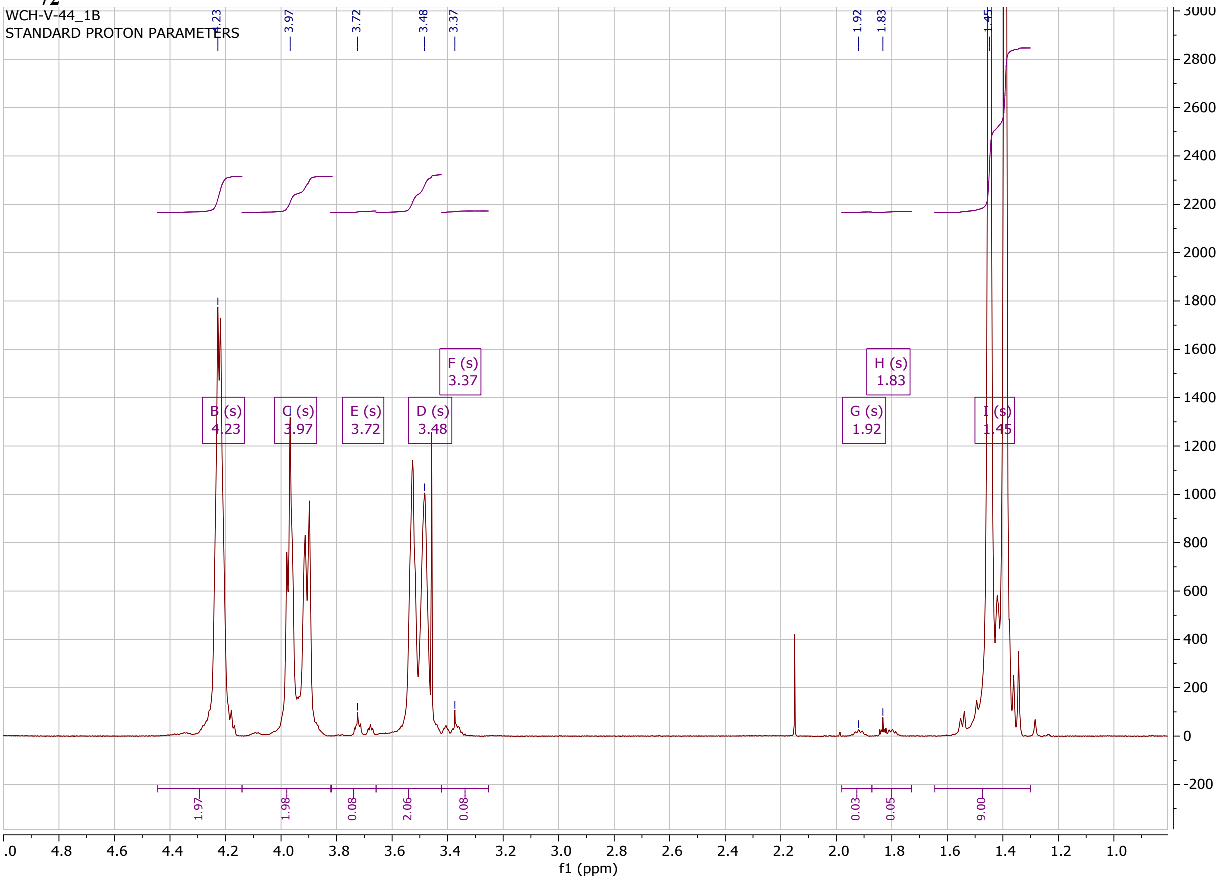

$P2_{49}$

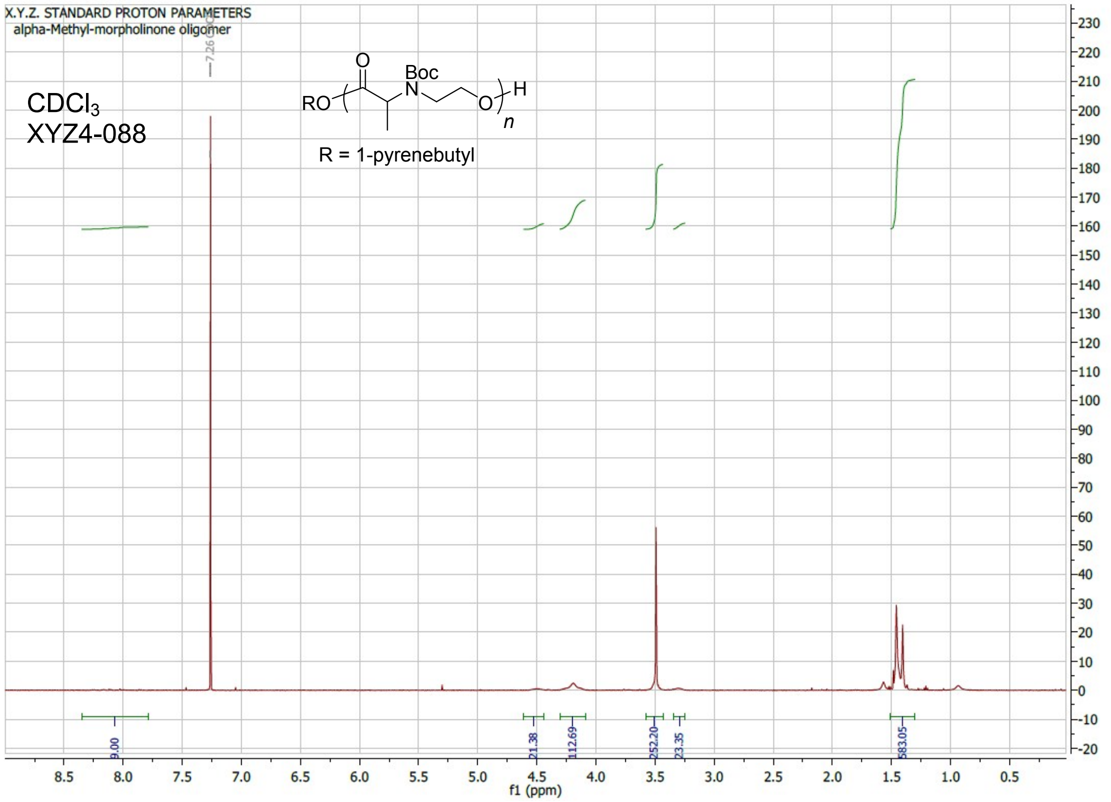

X.Y.Z. STANDARD PROTON PARAMETERS
alpha-Methyl-morpholinone oligomer
7.26
CDCl3
XYZ4-088
O
Boc
N
RO
O
H
n
R = 1-pyrenebutyl
9.00
21.38
112.69
252.20
23.35
583.05
f1 (ppm)

$P(2^{+}\ CF_3COO^{-})_{49}$

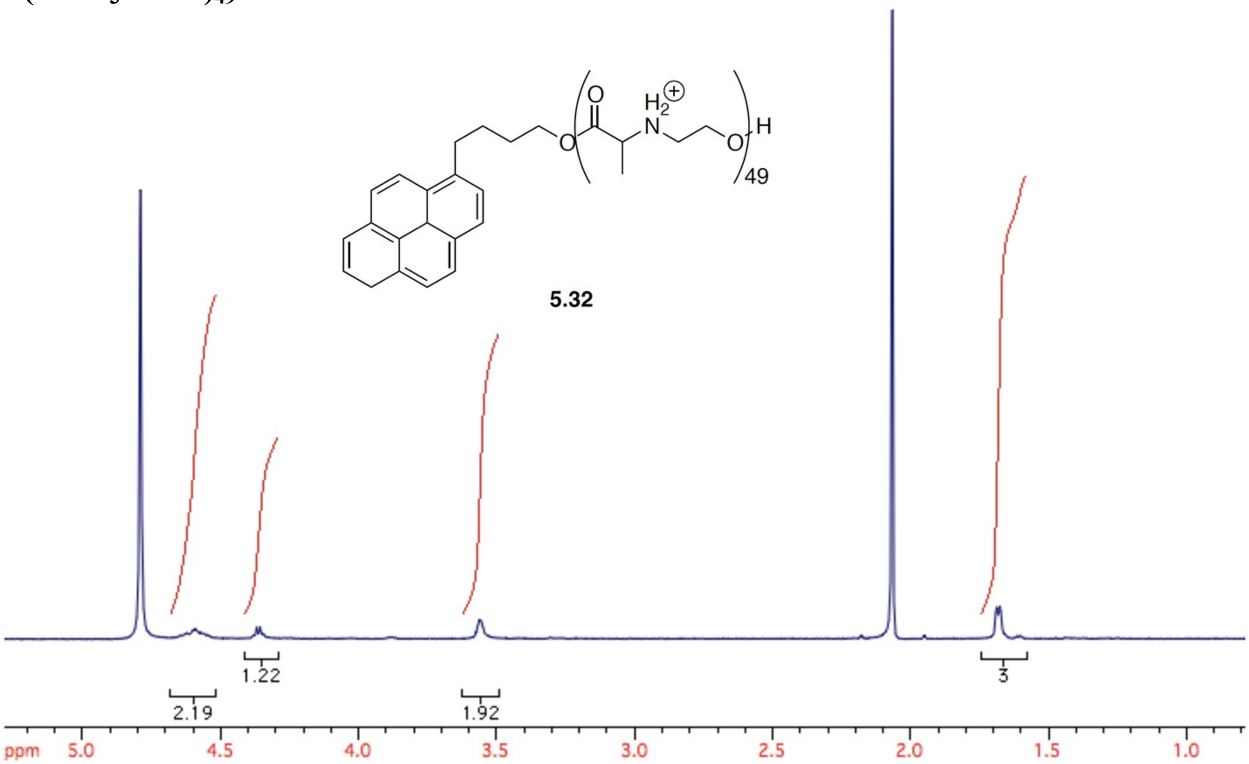

$P3_{75}$

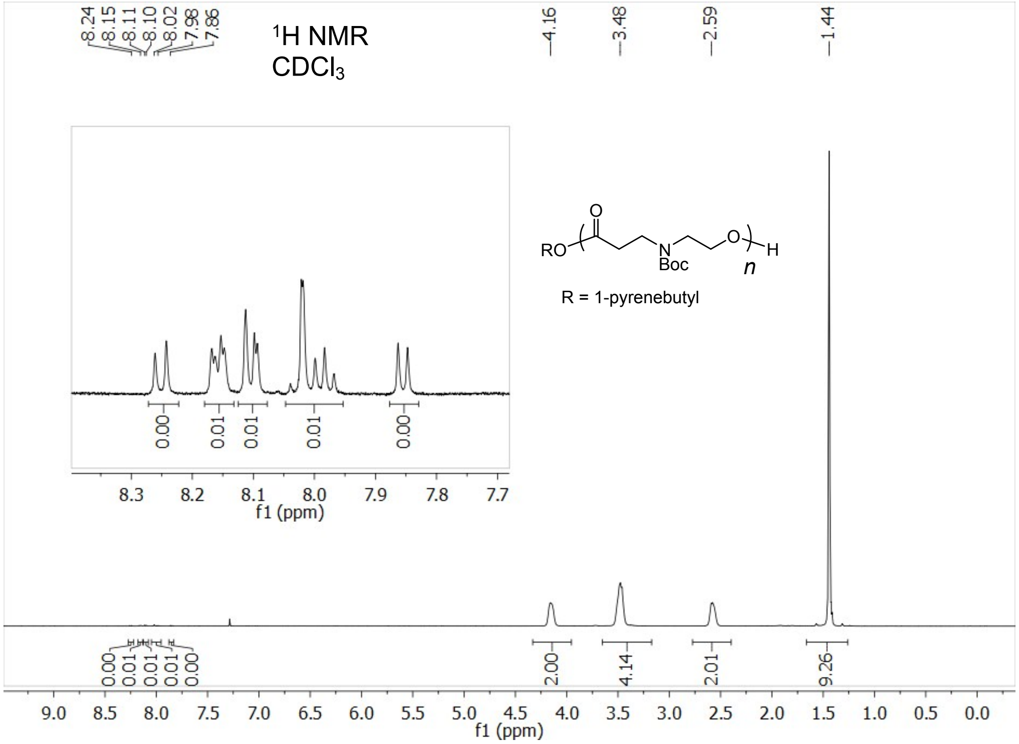

The aromatic resonances of the initiator 1-pyrenebutanol are shown in the inset.

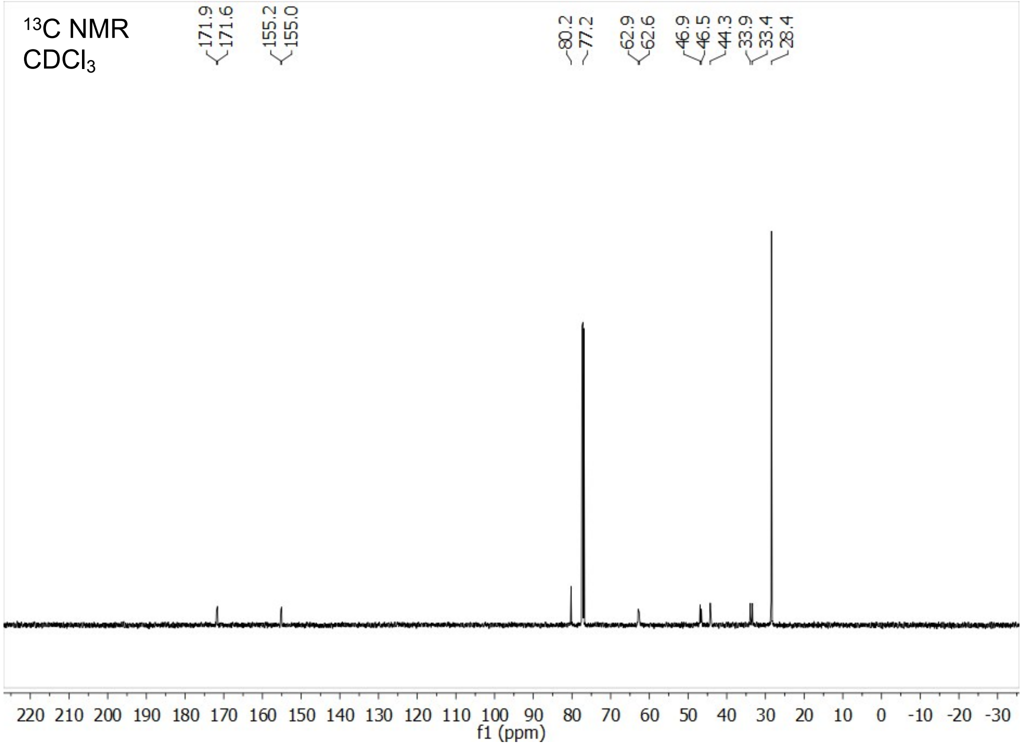

P(3⁺ CF₃COO⁻)₇₅

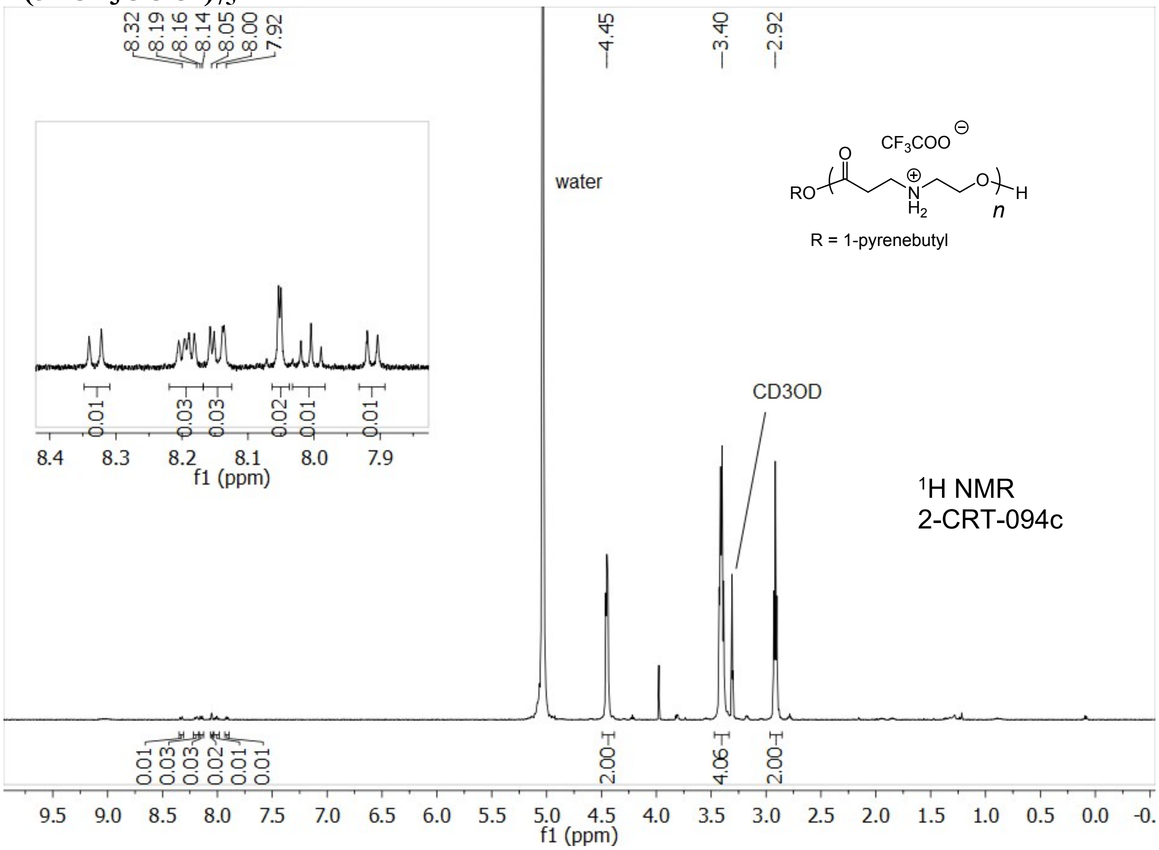

8.32
8.19
8.16
8.14
8.05
8.00
7.92
4.45
3.40
2.92
water
CD3OD
CF3COO
O
RO
N
H2
O
H
n
R = 1-pyrenebutyl
1H NMR
2-CRT-094c
0.01
0.03
0.03
0.02
0.01
0.01
8.4
8.3
8.2
8.1
8.0
7.9
f1 (ppm)
2.00
4.06
2.00
9.5
9.0
8.5
8.0
7.5
7.0
6.5
6.0
5.5
5.0
4.5
4.0
3.5
3.0
2.5
2.0
1.5
1.0
0.5
0.0
-0.
f1 (ppm)

P4$_{\mathit{40}}$

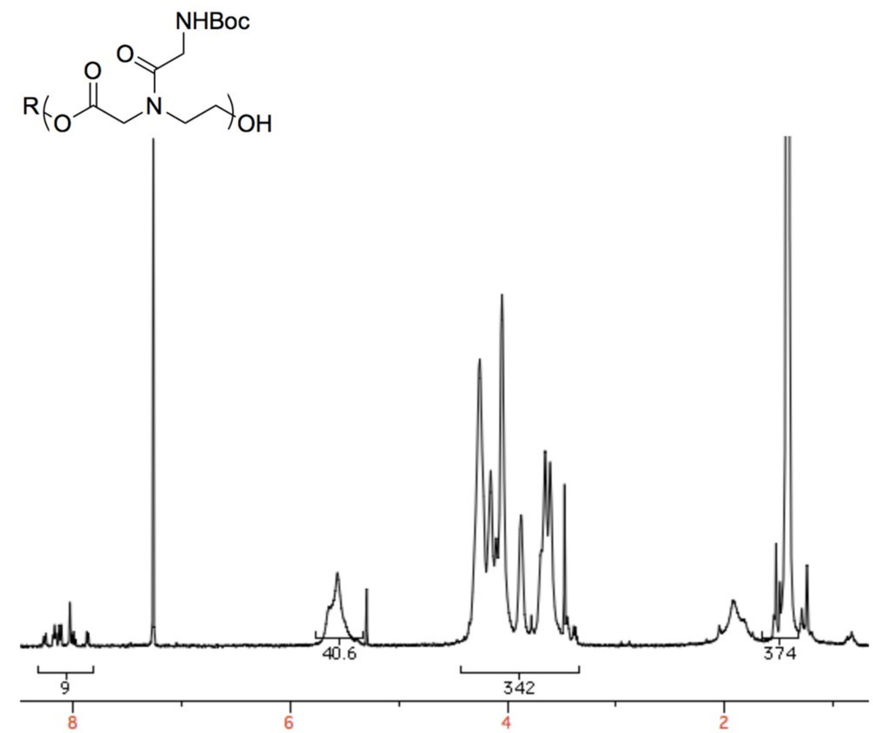

P4$^{+}_{\mathit{40}}$

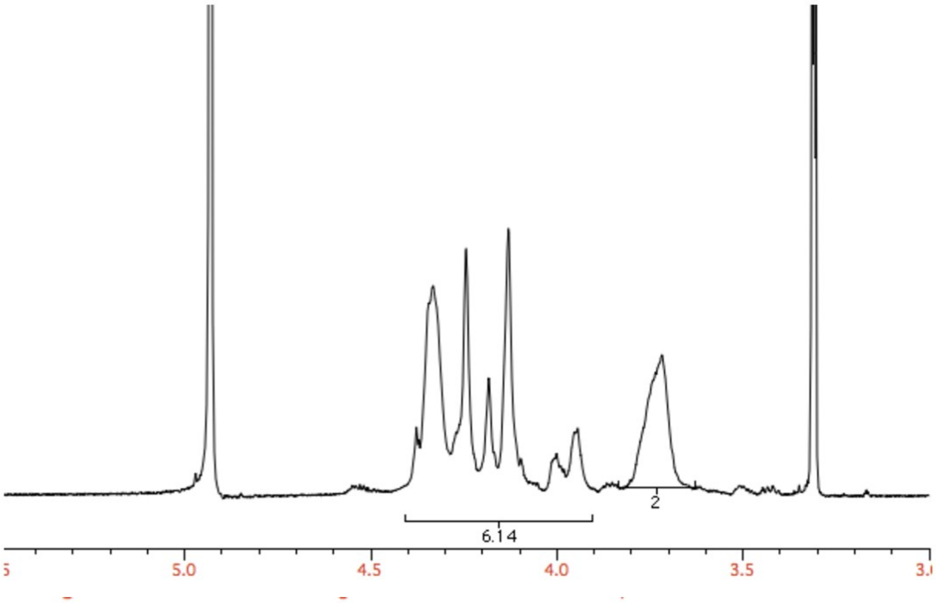

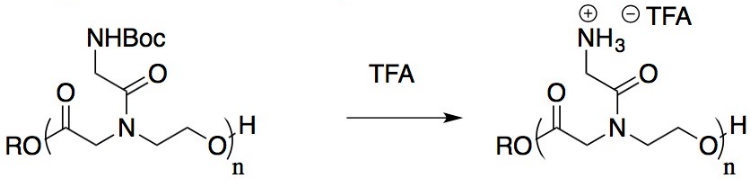

Copolymers

P(VL$_{49}$-*b*-1$_{74}$)

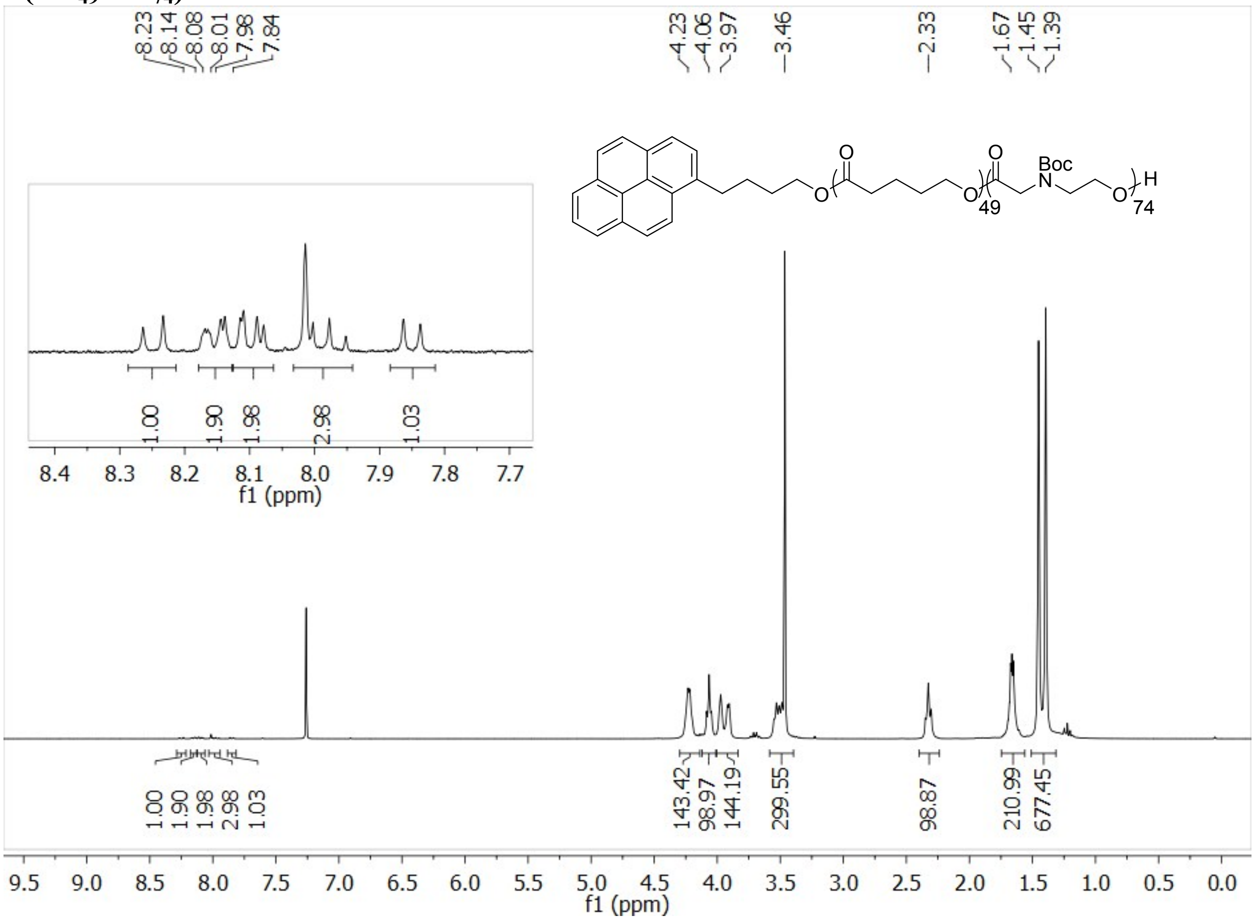

^{1}H NMR spectrum in chloroform-*d*. The aromatic resonances of the initiator 1-pyrenebutanol are shown in the inset.

P(1_9-*r*-2_{36}), 80% α-Me

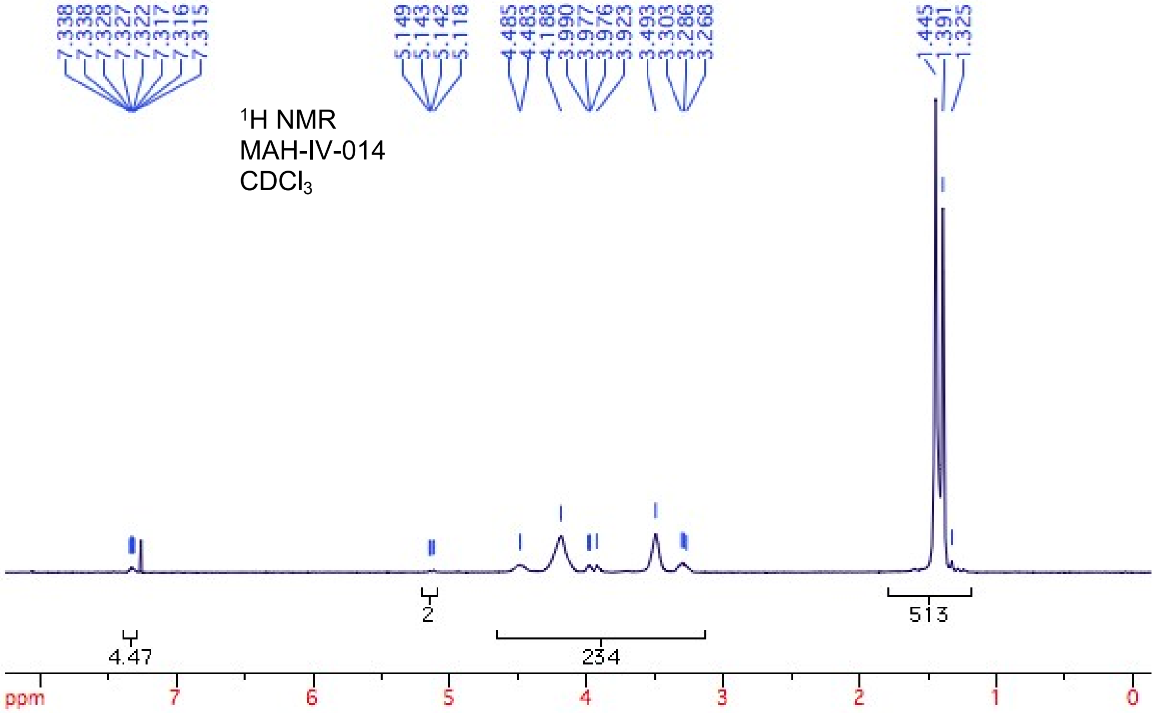

P(1_{51}-*r*-2_{60}), 54% α-Me

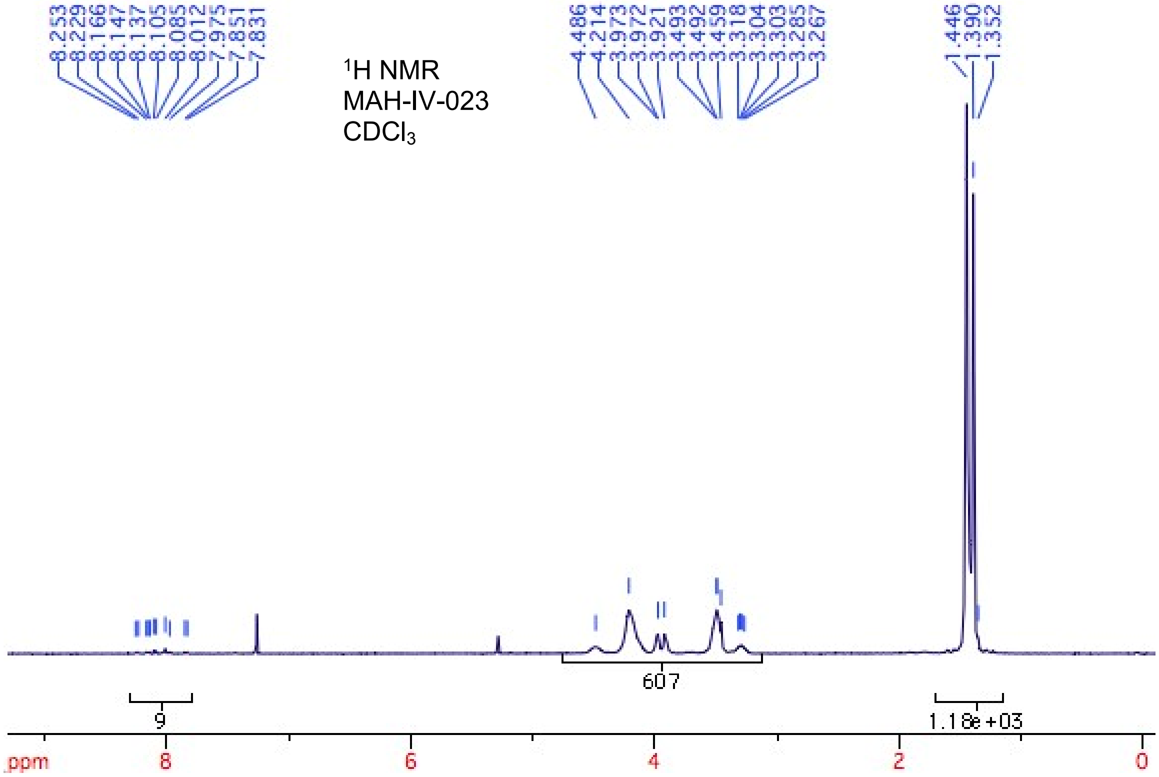

P(1_{44}-*r*-2_{23}), 35% α-Me

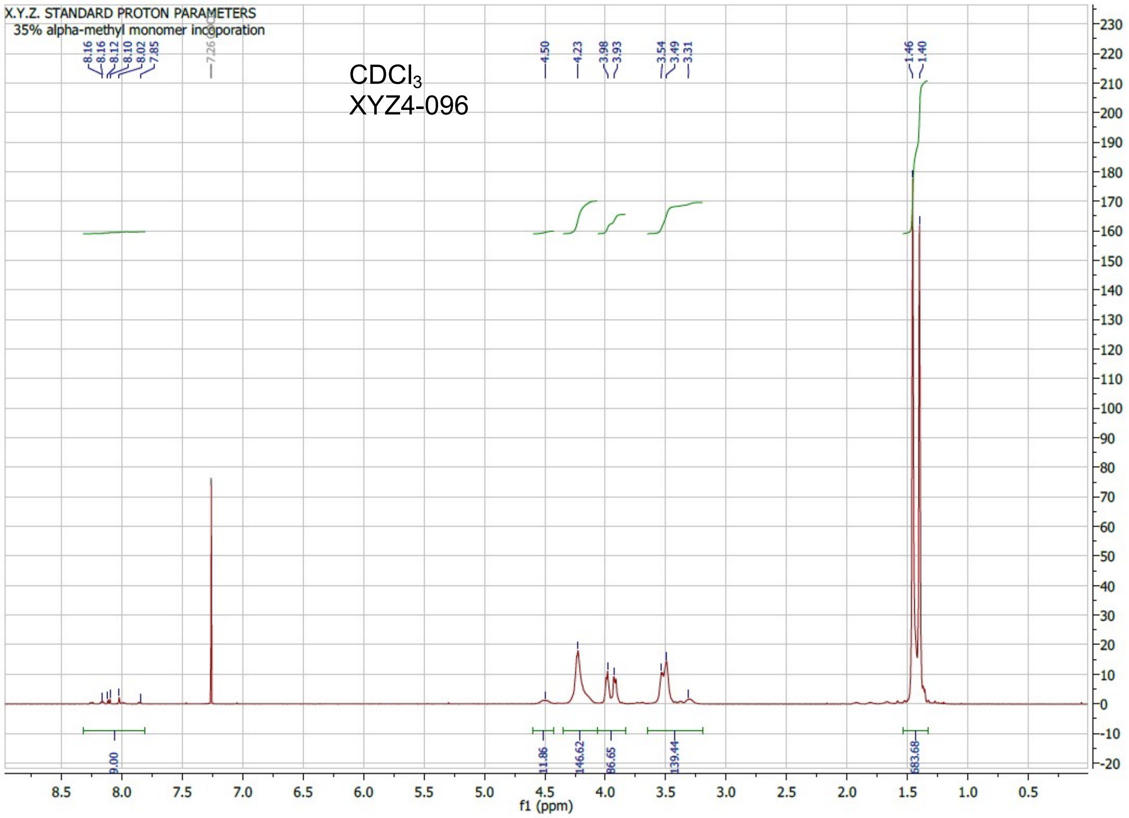

P((1$^+$ CF_3COO^-)$_9$-*r*-(2$^+$ CF_3COO^-)$_{36}$), the deprotected 80% α-Me copolymer

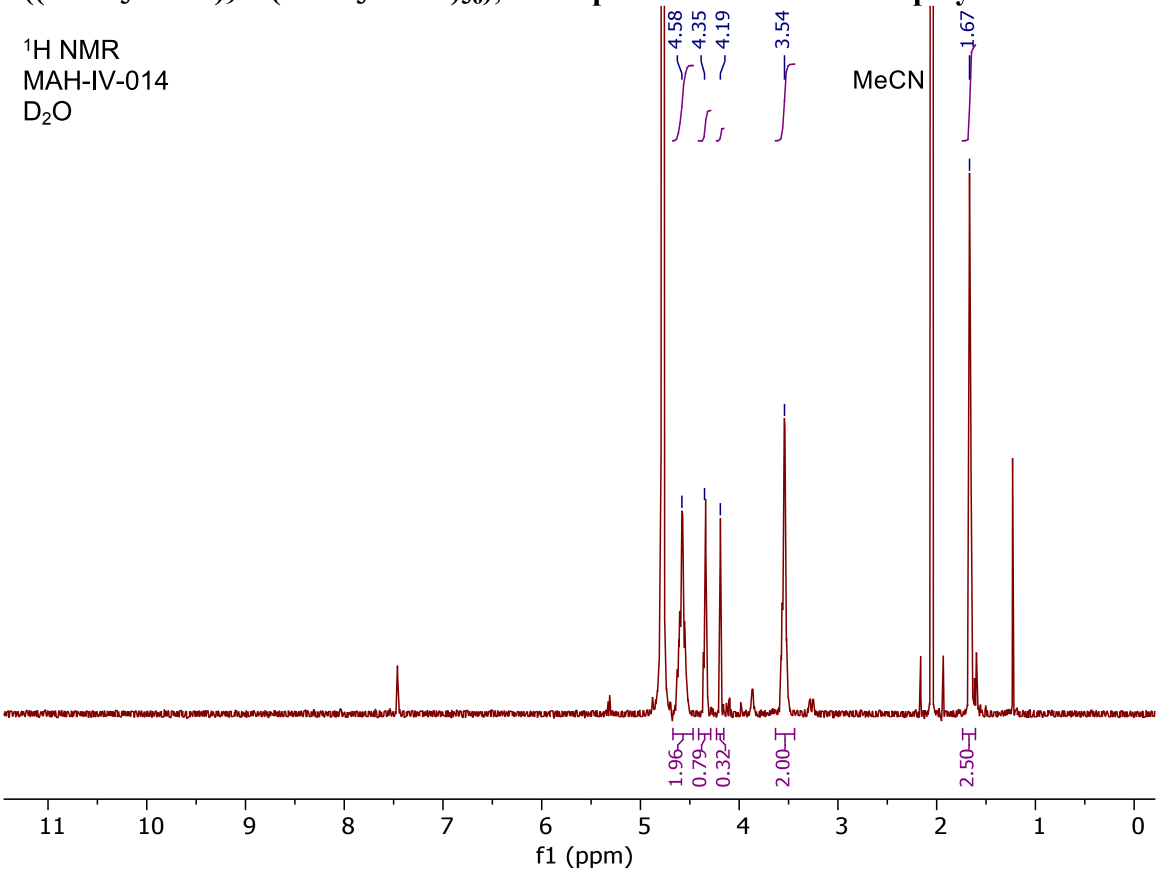

P((1^{+} CF_3COO^{-})$_{44}$-*r*-(2^{+} CF_3COO^{-})$_{23}$), the deprotected 35% α-Me copolymer

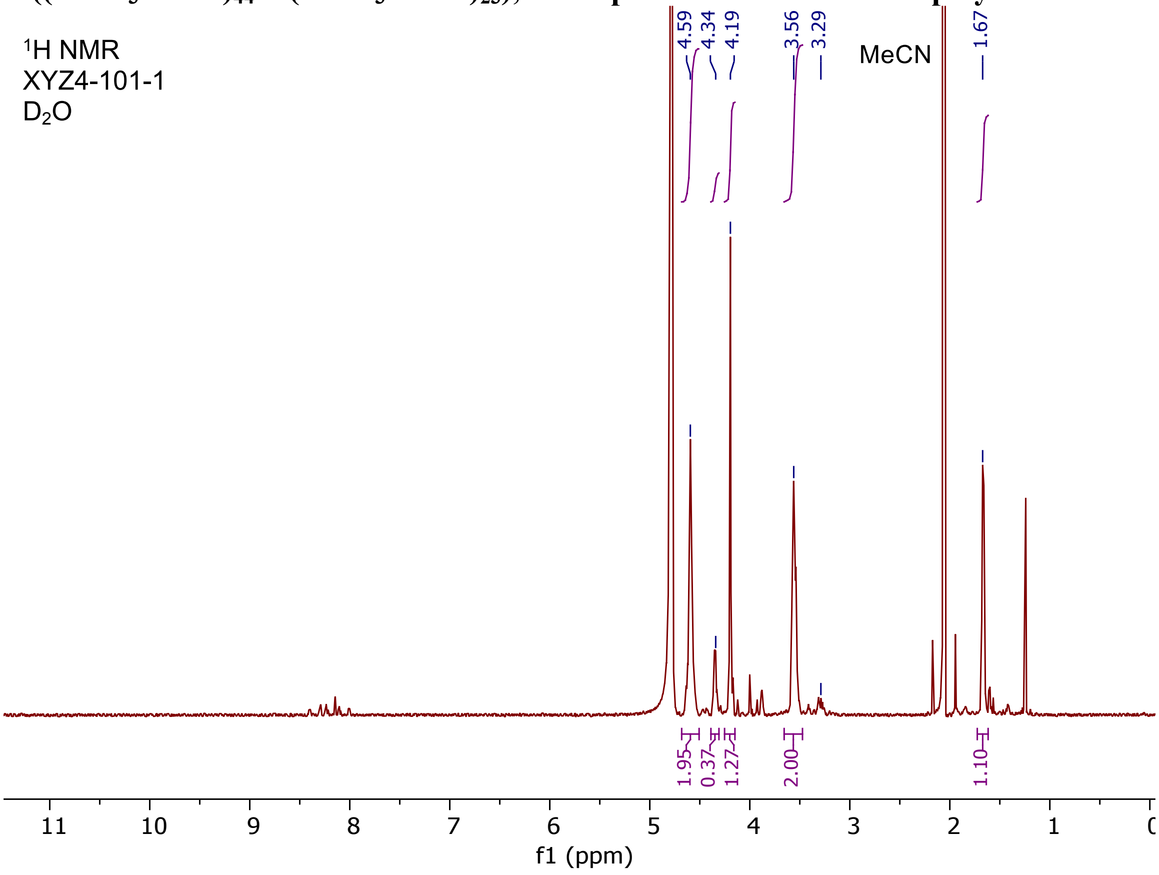

Other compounds

N-(2-hydroxyethyl)glycine (6a)

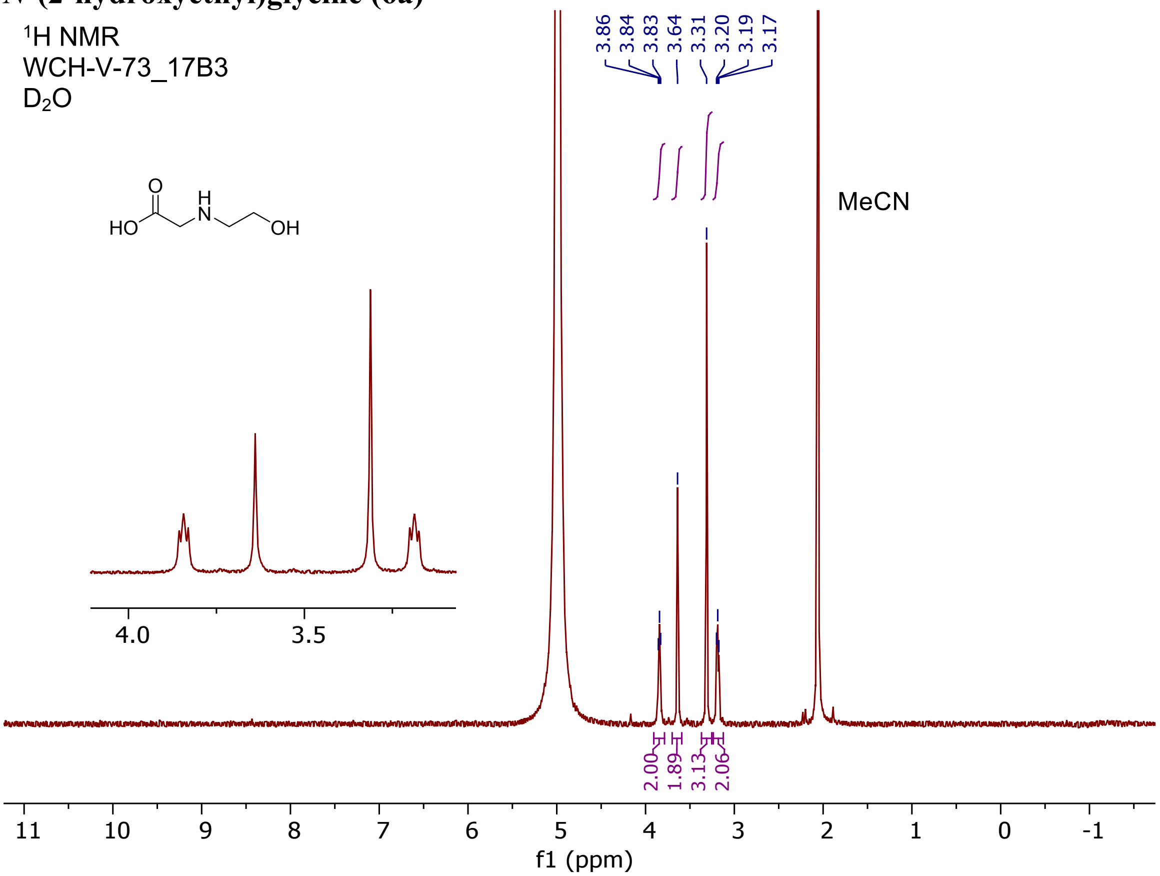

***N*-(2-hydroxyethyl)glycine methyl ester, trifluoracetic acid adduct (7)**

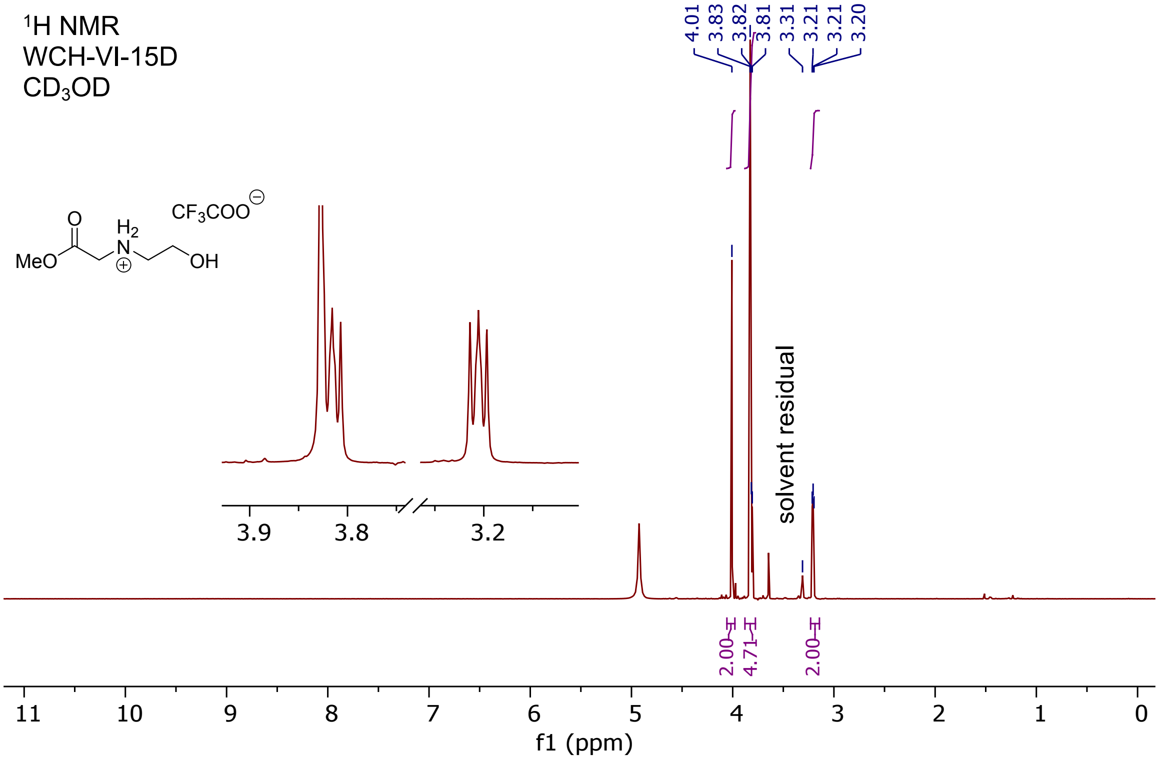

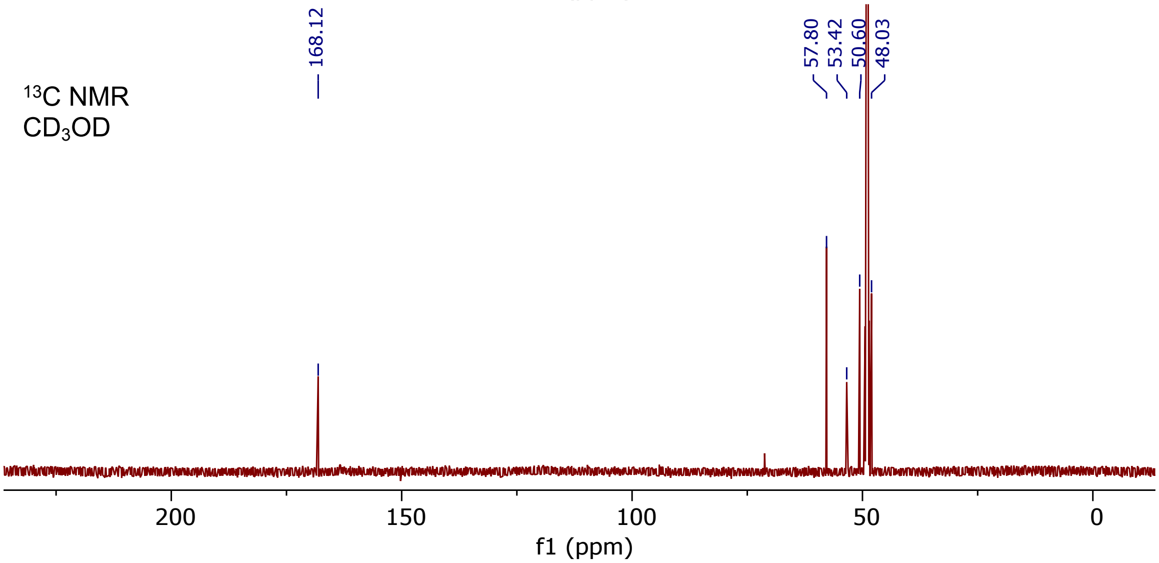

***N*-acetyl-*N*-(2-hydroxyethyl)glycine methyl ester (17)**

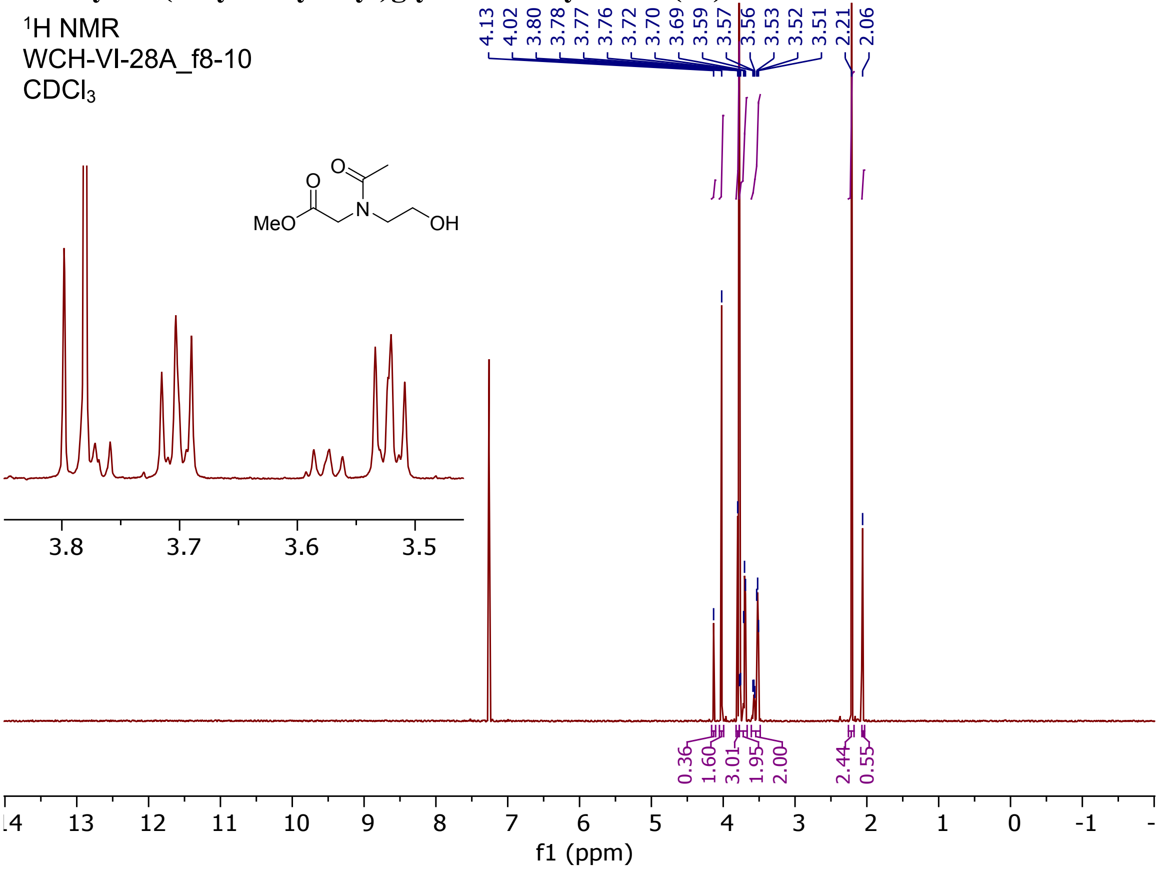

1-(2-hydroxyethyl)piperazine-2,5-dione (11a)

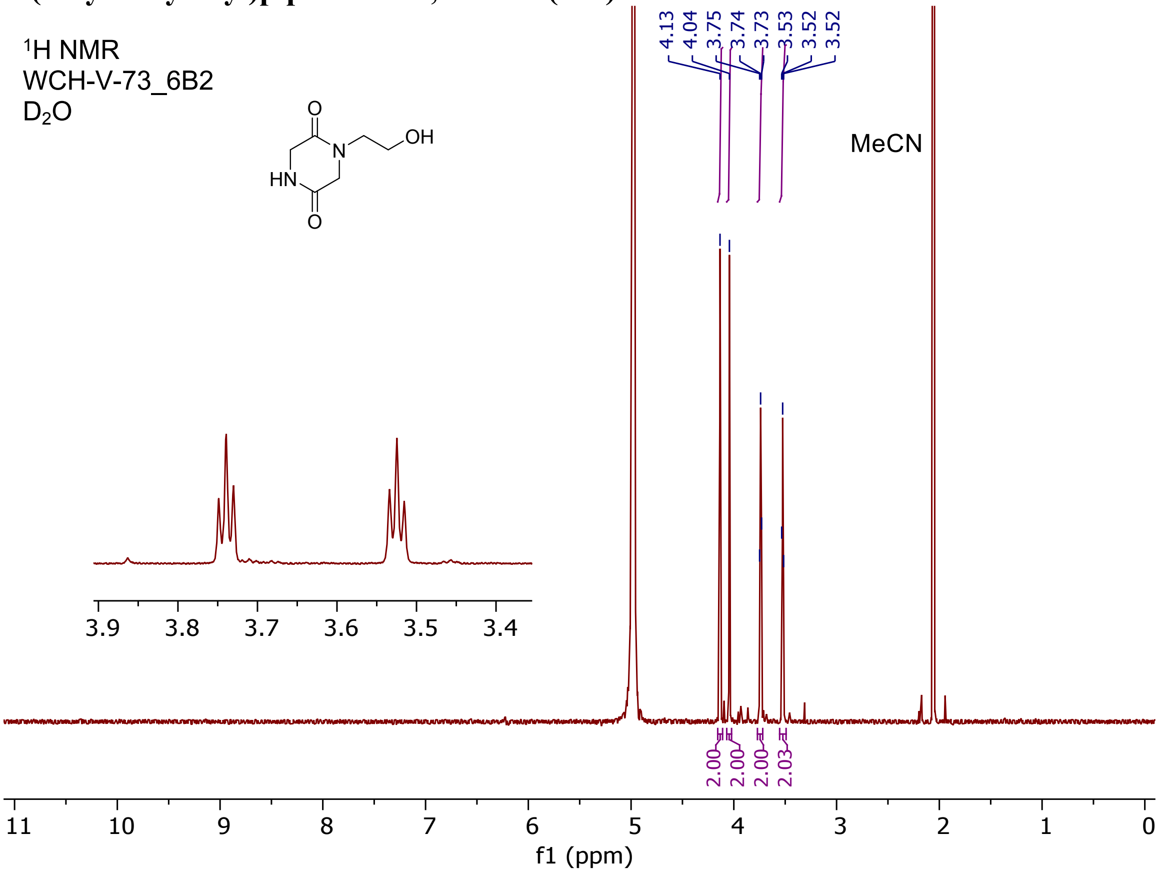

_N_-(2-hydroxyethyl)-_N_-glycylglycine (12a)

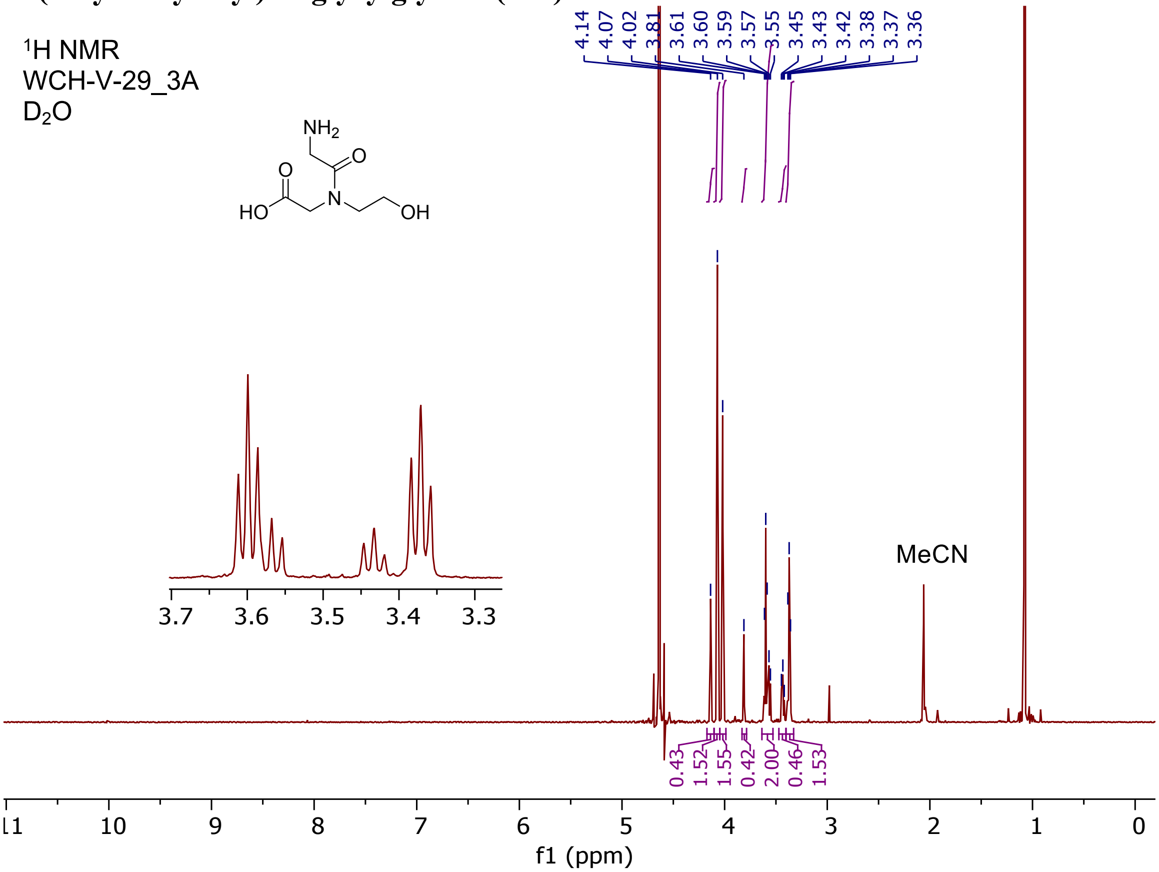

The δ 1.08 peak arises from the byproducts of Boc-deprotection.

N-(2-hydroxyethyl)-DL-alanine (8a)

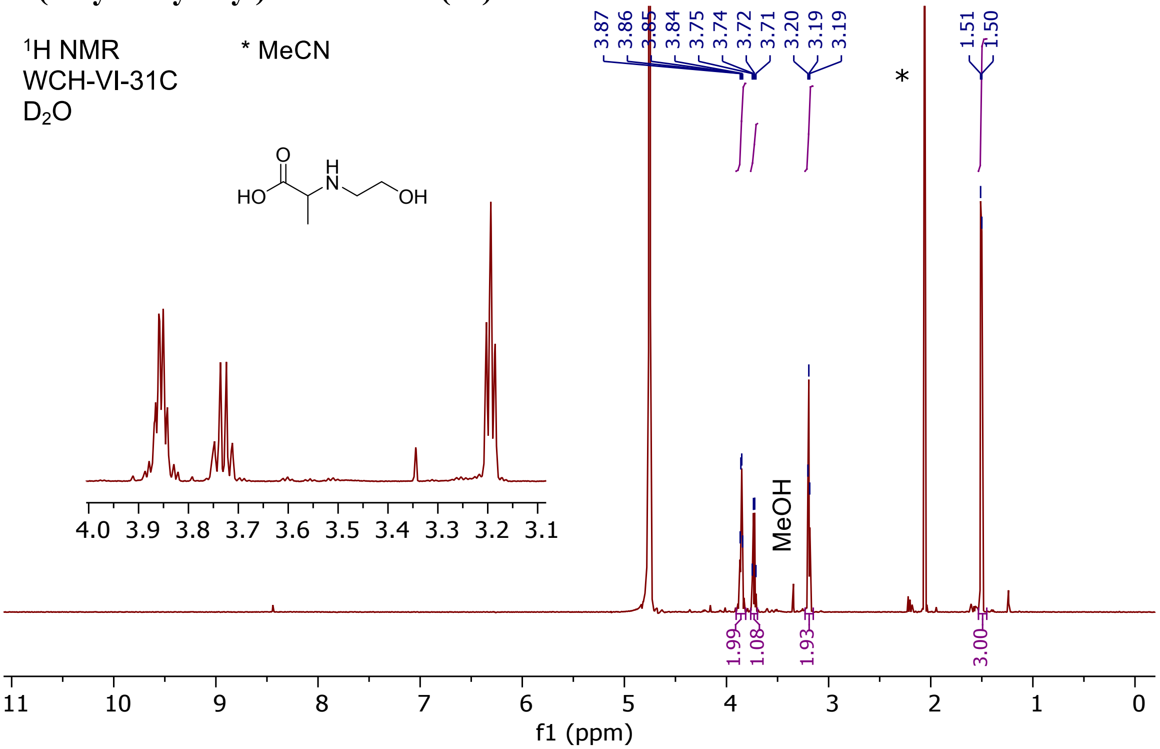

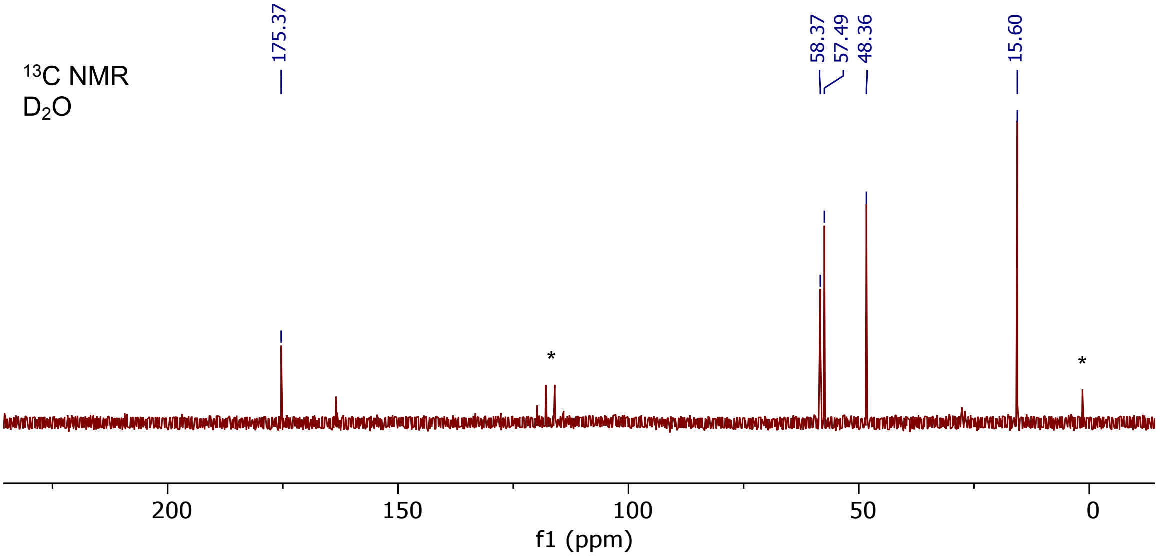

3-(2-hydroxyethylamino)propionic acid (10a)

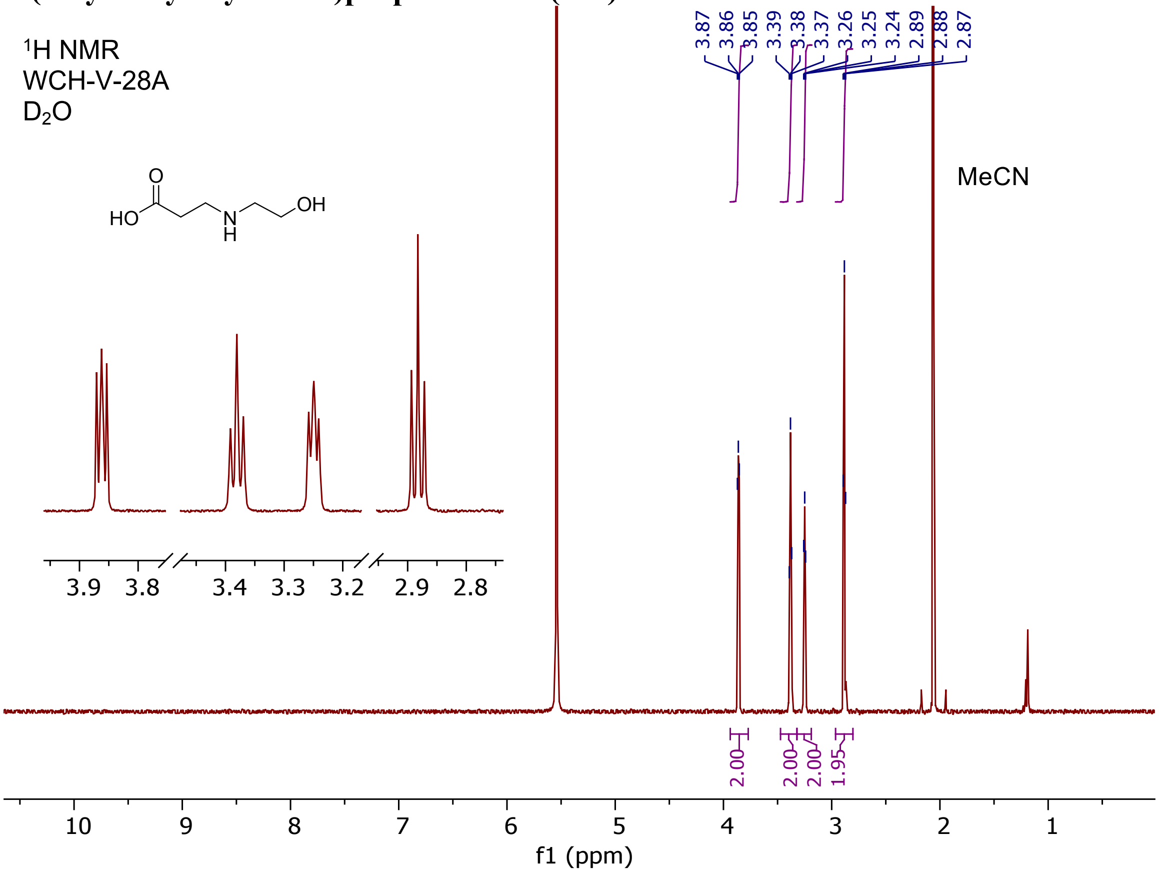

methyl 3-(2-hydroxyethylamino)propanoate, trifluoracetic acid adduct (18)

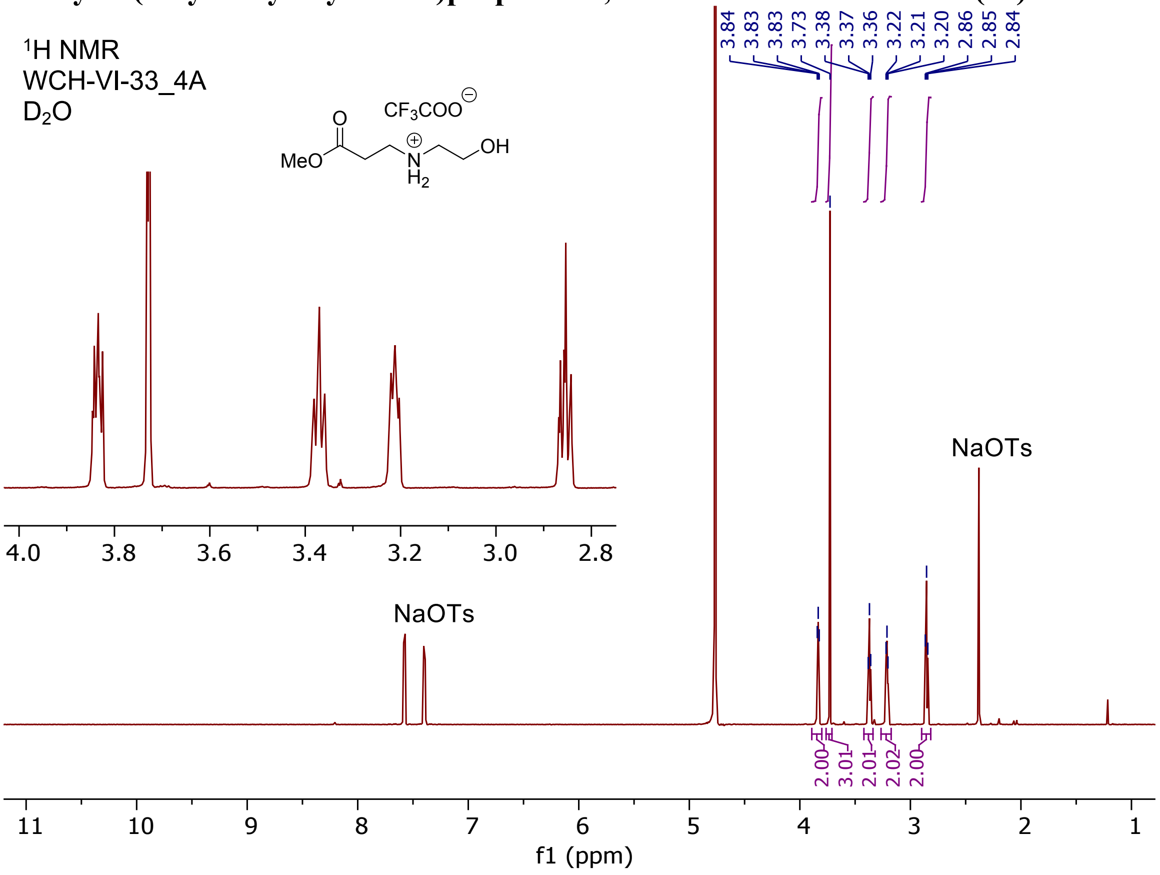

methyl *N*-acetyl-3-(2-hydroxyethylamino)propanoate (19)

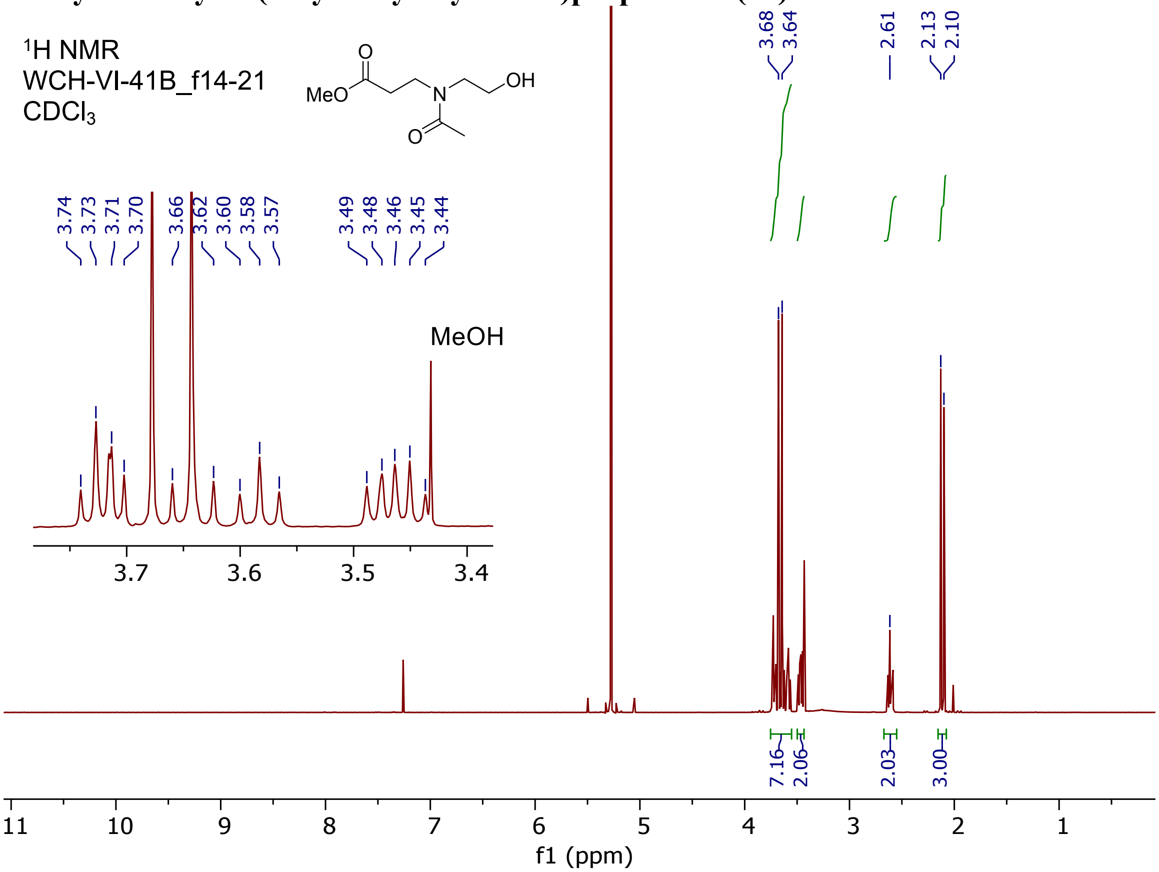

N-acetyl-*N*-(2-hydroxyethyl)glycine

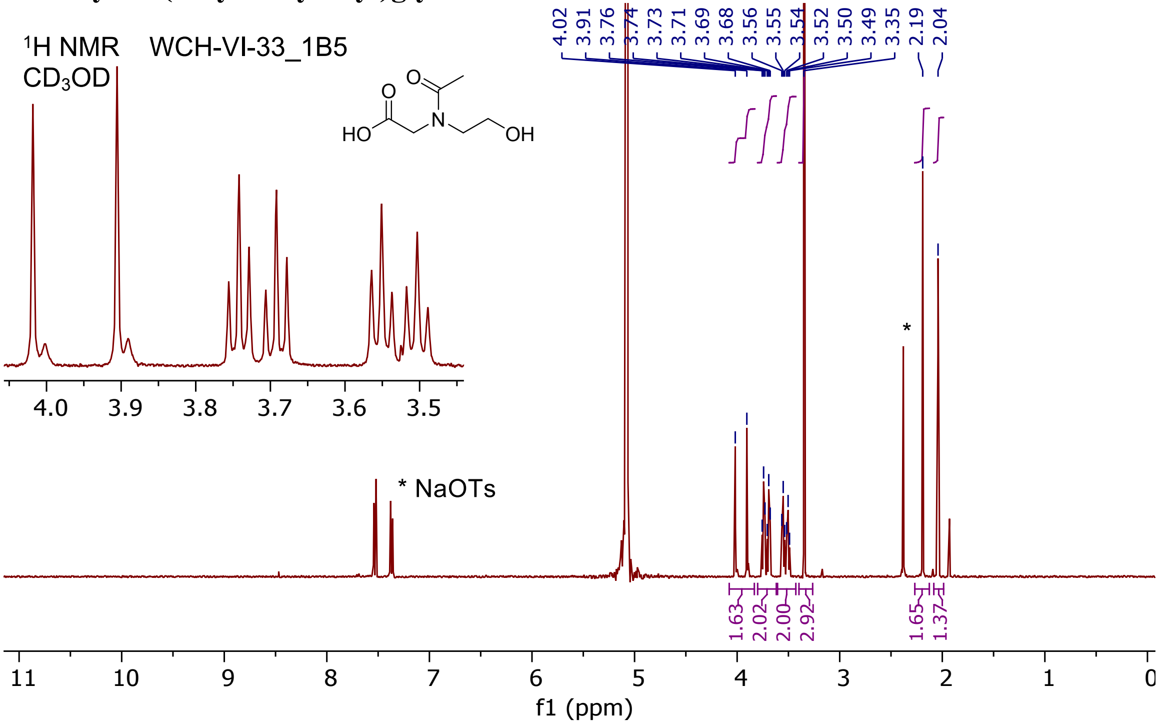

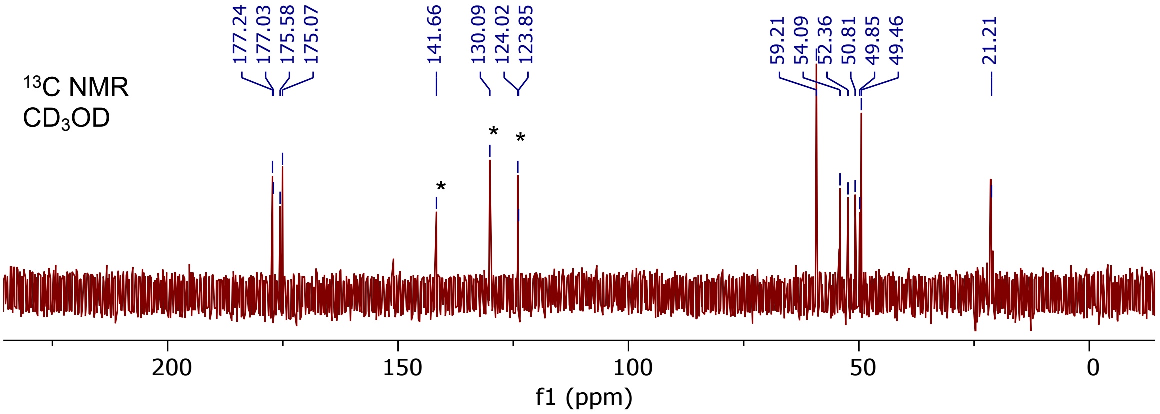

The peaks at δ 4.02 and 3.91 have slightly less area than expected (should be 2H in total) and have small upfield shoulders. This is caused by D_2O-mediated deuteration α to the carbonyl in methyl ester **17**, from which this compound was synthesized over a long period of hydrolysis.

N-acetyl-*N*-(2-hydroxyethyl)-3-aminopropanoic acid

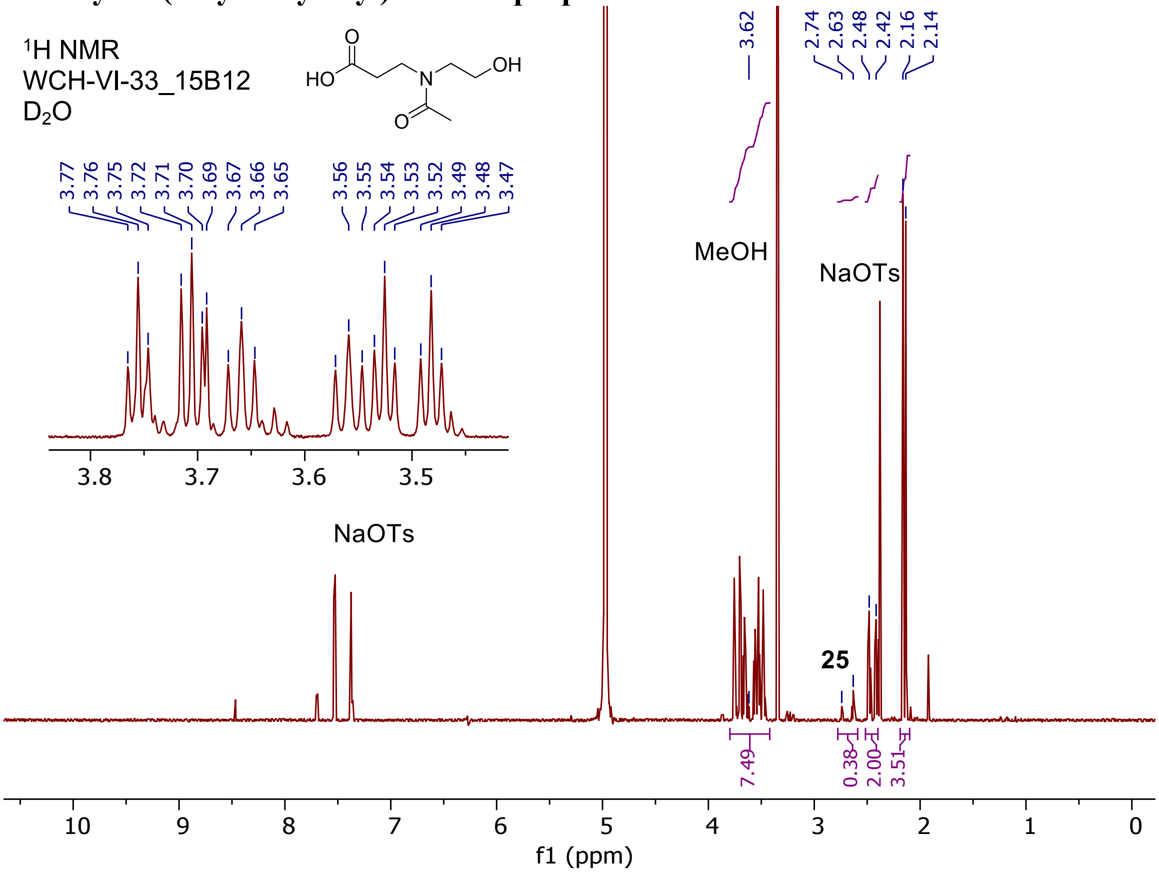

There is extra area under the δ 3.61 and 2.15 regions due to unreacted methyl ester **19**.

DKP XRD data

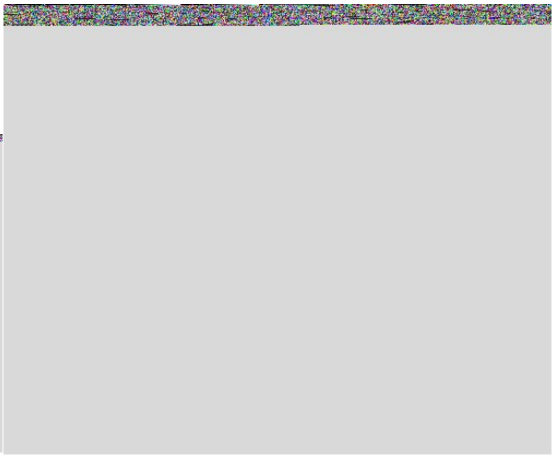

Crystal data

$C_8H_{14}N_2O_4$	
M_r = 202.21	D_x = 1.474 Mg m^{-3}
Monoclinic, $P2_1/c$	Melting point: ? K
	Mo *K*α radiation, λ = 0.71073 Å
a = 5.6483 (5) Å	Cell parameters from 3792 reflections
b = 9.0345 (7) Å	θ = 3.2–27.1°
c = 9.1851 (9) Å	μ = 0.12 mm^{-1}
β = 103.599 (4)°	*T* = 100 K
V = 455.57 (7) Å^3	Needle, colorless
Z = 2	0.09 × 0.09 × 0.07 mm
F(000) = 216	

Data collection

Bruker D8 Venture Diffractometer	902 reflections with $I > 2\sigma(I)$
Radiation source: sealed X-ray tube	R_{int} = 0.024
	θ_{max} = 27.1°, θ_{min} = 3.2°

OMEGA–PHI scans	$h = -7$ 7
Absorption correction: multi-scan *SADABS* (Sheldick, 1996)	$k = -11$ 11
T_{min} = ?, T_{max} = ?	$l = -11$ 11
8400 measured reflections	
1012 independent reflections	

Refinement

Refinement on F^2	
Least-squares matrix: full	Hydrogen site location: inferred from neighbouring sites
$R[F^2 > 2\sigma(F^2)] = 0.033$	H-atom parameters constrained
$wR(F^2) = 0.094$	$w = 1/[\sigma^2(F_o^2) + (0.0523P)^2 + 0.1576P]$ where $P = (F_o^2 + 2F_c^2)/3$
$S = 1.07$	$(\Delta/\sigma)_{max} < 0.001$
1012 reflections	$\Delta\rho_{max} = 0.33$ e Å^{-3}
65 parameters	$\Delta\rho_{min} = -0.16$ e Å^{-3}
0 restraints	Extinction correction: none

Fractional atomic coordinates and isotropic or equivalent isotropic displacement parameters (Å^2)

	x	*y*	*z*	U_{iso}*/U_{eq}
O4	0.79005 (14)	0.66043 (9)	0.86837 (9)	0.0187 (2)
N1	0.46137 (16)	0.51279 (10)	0.84336 (10)	0.0139 (2)
C6	0.65242 (19)	0.58518 (11)	0.92547 (12)	0.0142 (3)
C9	0.29250 (19)	0.42572 (12)	0.90625 (12)	0.0154 (3)
H9A	0.2874	0.3242	0.8651	0.019*
H9B	0.1277	0.4687	0.8711	0.019*
C5	0.40039 (19)	0.52948 (12)	0.67957 (11)	0.0161 (3)
H5A	0.3215	0.4379	0.6325	0.019*
H5B	0.5514	0.5451	0.6446	0.019*
C4	0.2295 (2)	0.66031 (13)	0.63255 (13)	0.0202 (3)

H4A	0.0657	0.6370	0.6477	0.024*
H4B	0.2926	0.7487	0.6931	0.024*
O2	0.21653 (17)	0.68771 (11)	0.47856 (10)	0.0295 (3)
H2	0.0889	0.7353	0.4413	0.044*

Atomic displacement parameters (Å^2)

	U^{11}	U^{22}	U^{33}	U^{12}	U^{13}	U^{23}
O4	0.0184 (4)	0.0216 (4)	0.0166 (4)	−0.0041 (3)	0.0054 (3)	0.0018 (3)
N1	0.0158 (5)	0.0151 (4)	0.0109 (4)	0.0000 (3)	0.0033 (3)	0.0007 (3)
C6	0.0154 (5)	0.0128 (5)	0.0145 (5)	0.0025 (4)	0.0040 (4)	0.0006 (4)
C9	0.0151 (5)	0.0171 (5)	0.0140 (5)	−0.0022 (4)	0.0030 (4)	0.0004 (4)
C5	0.0183 (5)	0.0185 (5)	0.0111 (5)	0.0007 (4)	0.0030 (4)	0.0007 (4)
C4	0.0213 (6)	0.0250 (6)	0.0150 (5)	0.0050 (4)	0.0060 (4)	0.0056 (4)
O2	0.0283 (5)	0.0442 (6)	0.0176 (5)	0.0147 (4)	0.0083 (4)	0.0129 (4)

Geometric parameters (Å, °)

O4—C6	1.2383 (13)	C5—C4	1.5231 (15)
N1—C6	1.3335 (14)	C5—H5A	0.9900
N1—C9	1.4565 (14)	C5—H5B	0.9900
N1—C5	1.4701 (13)	C4—O2	1.4208 (13)
C6—C9^{i}	1.5065 (14)	C4—H4A	0.9900
C9—C6^{i}	1.5064 (14)	C4—H4B	0.9900
C9—H9A	0.9900	O2—H2	0.8400
C9—H9B	0.9900		
C6—N1—C9	123.98 (9)	N1—C5—H5A	109.6
C6—N1—C5	119.49 (9)	C4—C5—H5A	109.6
C9—N1—C5	116.33 (9)	N1—C5—H5B	109.6
O4—C6—N1	122.34 (10)	C4—C5—H5B	109.6
O4—C6—C9^{i}	118.14 (9)	H5A—C5—H5B	108.1
N1—C6—C9^{i}	119.52 (9)	O2—C4—C5	107.35 (9)
N1—C9—C6^{i}	116.47 (9)	O2—C4—H4A	110.2

N1—C9—H9A	108.2	C5—C4—H4A	110.2
$C6^i$—C9—H9A	108.2	O2—C4—H4B	110.2
N1—C9—H9B	108.2	C5—C4—H4B	110.2
$C6^i$—C9—H9B	108.2	H4A—C4—H4B	108.5
H9A—C9—H9B	107.3	C4—O2—H2	109.5
N1—C5—C4	110.49 (8)		

Symmetry code: (i) $-x+1, -y+1, -z+2$.

All esds (except the esd in the dihedral angle between two l.s. planes) are estimated using the full covariance matrix. The cell esds are taken into account individually in the estimation of esds in distances, angles and torsion angles; correlations between esds in cell parameters are only used when they are defined by crystal symmetry. An approximate (isotropic) treatment of cell esds is used for estimating esds involving l.s. planes.

References

1. Ho, W. C.; Chung, K.; Ingram, A. J.; Waymouth, R. M., Pd-Catalyzed Aerobic Oxidation Reactions: Strategies To Increase Catalyst Lifetimes. *Journal of the American Chemical Society* **2018,** *140* (2), 748-757.
2. Pratt, R. C.; Lohmeijer, B. G. G.; Long, D. A.; Lundberg, P. N. P.; Dove, A. P.; Li, H.; Wade, C. G.; Waymouth, R. M.; Hedrick, J. L., Exploration, Optimization, and Application of Supramolecular Thiourea−Amine Catalysts for the Synthesis of Lactide (Co)polymers. *Macromolecules* **2006,** *39* (23), 7863-7871.
3. Blake, T. R.; Waymouth, R. M., Organocatalytic Ring-Opening Polymerization of Morpholinones: New Strategies to Functionalized Polyesters. *Journal of the American Chemical Society* **2014,** *136* (26), 9252-9255.
4. Rosenberg, S. H.; Rapoport, H., Intramolecular Michael reactions. Addition to the α-carbon of ynamides. *The Journal of Organic Chemistry* **1985,** *50* (21), 3979-3982.
5. Greiner, B.; Breipohl, G.; Uhlmann, E., Influence of the Type of Junction in DNA-3′-Peptide Nucleic Acid (PNA) Chimeras on Their Binding Affinity to DNA and RNA. *Helvetica Chimica Acta* **1999,** *82* (12), 2151-2159.
6. Schwieger Jochen, F.; Unterhalt, B., *N*-Phosphorylierte Aminosäuren als potentielle Zytostatika. *Archiv der Pharmazie* **1992,** *325* (11), 709-715.
7. Pestov, A. V.; Peresypkina, E. V.; Virovets, A. V.; Podberezskaya, N. V.; Yatluk, Y. G.; Skorik, Y. A., Bis[*N*-(2-hydroxyethyl)-β-alaninato]copper(II). *Acta Crystallographica Section C* **2005,** *61* (Pt 12), m510-2.
8. Akita, Tetsuya; Suzuki, Makoto. Kawaken Fine Chem Co. Ltd. Humectant, and hair cosmetic, skin cosmetic and bathing agent containing the same. Patent JP2010285411A, 2010-12-24. We assume that their assignment of 2H to the methyl peak at 2.68 ppm is a typo.
9. Erickson, Harold P. Size and Shape of Protein Molecules at the Nanometer Level Determined by Sedimentation, Gel Filtration, and Electron Microscopy. *Biological Procedures Online*, **2009**, Vol. 11, pp 32-51.
